# Reversible Monoacylglycerol Lipase Inhibitors: Discovery of a New Class of Benzylpiperidine Derivatives

**DOI:** 10.1021/acs.jmedchem.1c01806

**Published:** 2022-05-06

**Authors:** Giulia Bononi, Miriana Di Stefano, Giulio Poli, Gabriella Ortore, Philip Meier, Francesca Masetto, Isabella Caligiuri, Flavio Rizzolio, Marco Macchia, Andrea Chicca, Amir Avan, Elisa Giovannetti, Chiara Vagaggini, Annalaura Brai, Elena Dreassi, Massimo Valoti, Filippo Minutolo, Carlotta Granchi, Jürg Gertsch, Tiziano Tuccinardi

**Affiliations:** † Department of Pharmacy, University of Pisa, Via Bonanno 6, 56126 Pisa, Italy; ‡ Department of Life Sciences, University of Siena, Via Aldo Moro, 2, 53100 Siena, Italy; § Institute of Biochemistry and Molecular Medicine, NCCR TransCure, University of Bern, CH-3012 Bern, Switzerland; ∥ Department of Medical Oncology, VU University Medical Center, Cancer Center Amsterdam, DeBoelelaan 1117, 1081HV Amsterdam, The Netherlands; ⊥ Pathology Unit, Centro di Riferimento Oncologico di Aviano (CRO) IRCCS, 33081 Aviano, Italy; # Department of Molecular Sciences and Nanosystems, Ca’ Foscari University, 30123 Venezia, Italy; ∇ Metabolic Syndrome Research Center, Mashhad University of Medical Science, Mashhad 91886-17871, Iran; ○ Cancer Pharmacology Lab, Fondazione Pisana per la Scienza, via Giovannini 13, 56017 San Giuliano Terme, Pisa, Italy; ◆ Department of Biotechnology, Chemistry and Pharmacy, University of Siena, Via Aldo Moro, 2, 53100 Siena, Italy; ¶ Center for Instrument Sharing of the University of Pisa (CISUP), Lungarno Pacinotti 43, 56126 Pisa, Italy

## Abstract

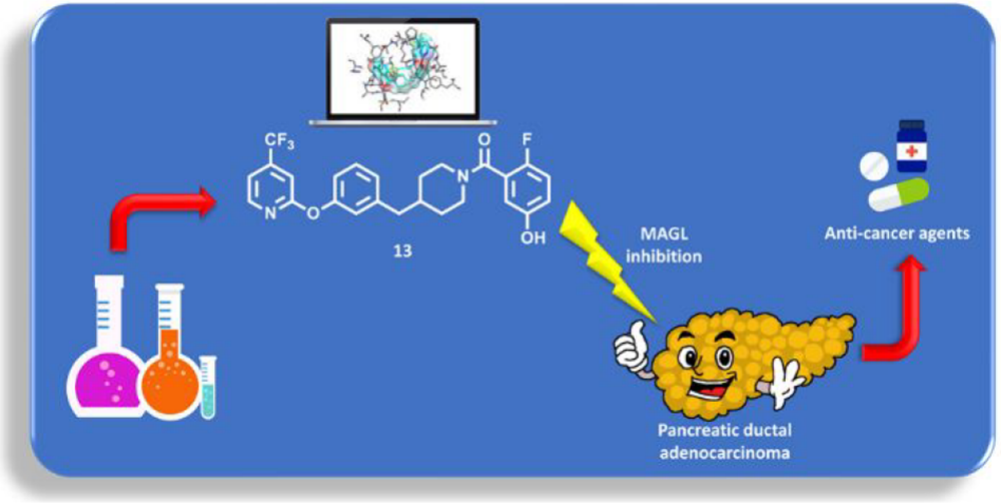

Monoacylglycerol lipase (MAGL) is the enzyme responsible for the metabolism of 2-arachidonoylglycerol in the brain and the hydrolysis of peripheral monoacylglycerols. Many studies demonstrated beneficial effects deriving from MAGL inhibition for neurodegenerative diseases, inflammatory pathologies, and cancer. MAGL expression is increased in invasive tumors, furnishing free fatty acids as pro-tumorigenic signals and for tumor cell growth. Here, a new class of benzylpiperidine-based MAGL inhibitors was synthesized, leading to the identification of **13**, which showed potent reversible and selective MAGL inhibition. Associated with MAGL overexpression and the prognostic role in pancreatic cancer, derivative **13** showed antiproliferative activity and apoptosis induction, as well as the ability to reduce cell migration in primary pancreatic cancer cultures, and displayed a synergistic interaction with the chemotherapeutic drug gemcitabine. These results suggest that the class of benzylpiperidine-based MAGL inhibitors have potential as a new class of therapeutic agents and MAGL could play a role in pancreatic cancer.

## 1 Introduction

Endocannabinoids (eCBs) are signaling lipophilic molecules that exert their biological actions by interacting with cannabinoid receptors (CB) CB1 and CB2. The most important endocannabinoid neuromodulators are represented by anandamide (AEA) and 2-arachidonoylglycerol (2-AG), which regulate several physiological and pathological processes including neuroinflammation, pain, neurodegeneration, and tumor progression.^1−3^ These arachidonic acid-derived lipids are synthesized on demand from membrane phospholipid precursors and are quickly metabolized after their release into the extracellular space and reuptake into the cytoplasm.^4^ Anandamide degradation is operated by fatty acid amide hydrolase (FAAH), a serine hydrolase localized in postsynaptic neurons, which metabolizes AEA into arachidonic acid (AA) and ethanolamine.^5^ On the other hand, 2-arachidonoylglycerol is hydrolyzed by serine hydrolases monoacylglycerol lipase (MAGL) and α/β hydrolase-6 and -12 (ABHD6 and ABHD12). MAGL is the main contributor to 2-AG degradation by hydrolyzing about 85% of 2-AG into AA and glycerol; the remaining 15% is hydrolyzed by ABHD6 and ABDH12.^6,7^ MAGL belongs to the α/β hydrolase superfamily and it is highly expressed in presynaptic neurons, but also in peripheral tissues such as kidney, ovaries, testis, adrenal glands, adipose tissue, and heart.^8^ This enzyme plays an essential role in the modulation of eCBs and the eicosanoid signaling pathway, as demonstrated in several pharmacological and genetic studies.^9^ Increased levels of AA, determined by MAGL hydrolyzing activity, promote the production of thromboxanes, prostaglandins, and other eicosanoids with pro-inflammatory activity.^10^ Furthermore, MAGL is overexpressed in aggressive cancer cells and primary tumors, where it modulates an oncogenic signaling network to generate protumorigenic lipids, which favor cancer invasiveness, migration, and growth.^11^ eCBs AEA and 2-AG also act as antinociceptive agents and may participate in the control of pain initiation.^2,12^ The level of eCBs increases in CNS under inflammatory and neuropathic pain conditions, and this may be due to the response of an endogenous neuroprotective mechanism to a pathological condition. In particular, 2-AG exerted its analgesic effect by stimulating CB2 receptors; therefore, MAGL modulation may influence pain.^13^ MAGL inhibition has been shown to alleviate allodynia in some neuropathic pain models.^14−16^ Thus, MAGL represents a feasible and promising therapeutic target for the treatment of neurodegenerative diseases, inflammation, pain, and cancer.^17^

**Figure 1:**
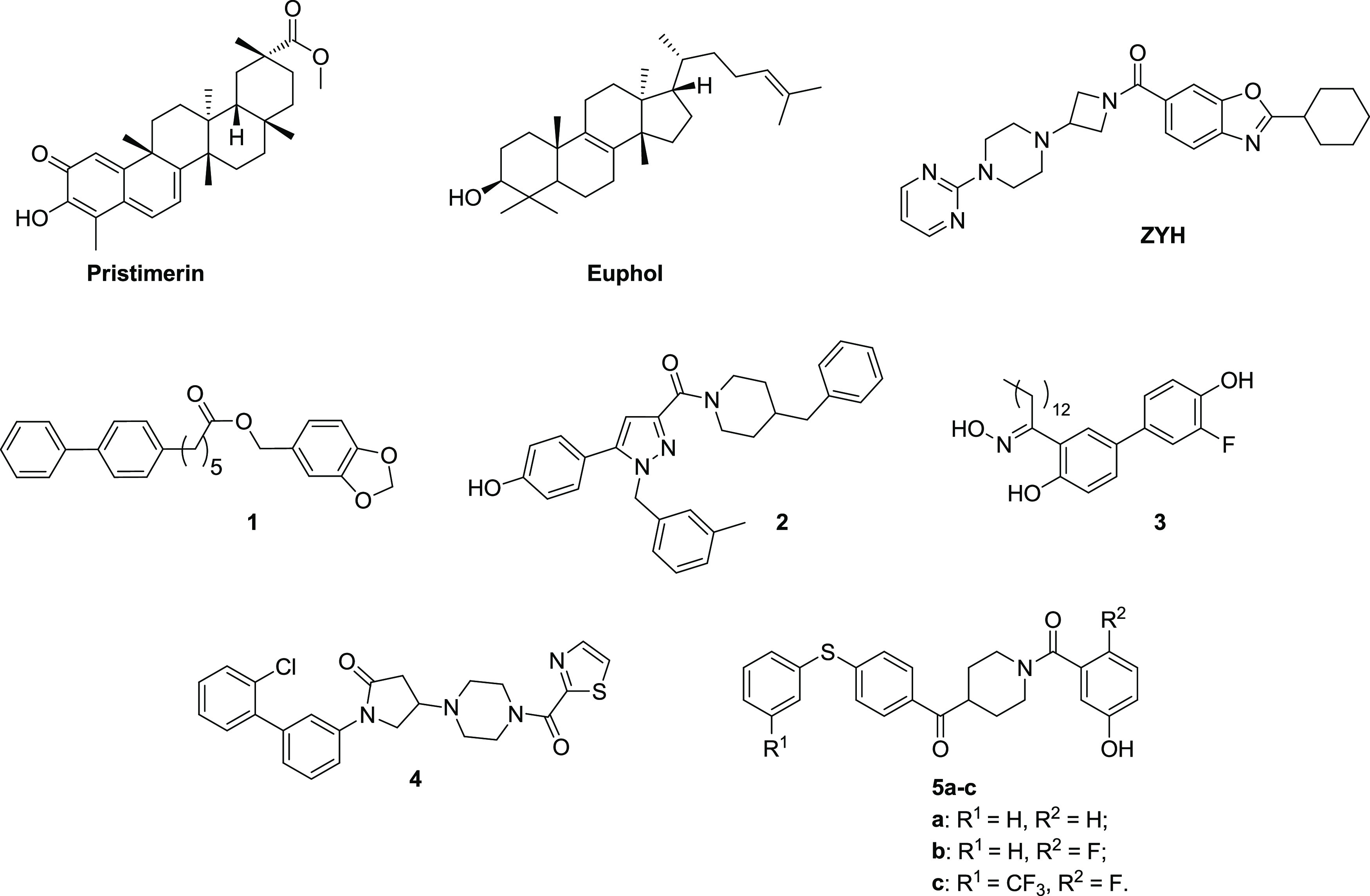
Structures of some representative synthetic reversible MAGL inhibitors.

The research field aimed at developing new MAGL inhibitors is increasing in the last years, mainly focusing on reversible inhibitors as potential therapeutic agents.^8^ Reversible inhibitors (i.e., those compounds that bind temporarily to MAGL, disabling its catalytic activity for a limited period of time) allow maintaining physiological levels of MAGL after its time-limited inhibition, thus avoiding a chronic MAGL blockade (typical of irreversible inhibitors), which leads to desensitization of CB1 receptors provoked by excessive concentrations of 2-AG. In addition, it was found that genetic deletion of MAGL as well as prolonged MAGL inhibition by small molecules determines an impaired CB1-dependent synaptic plasticity, cross-tolerance to exogenous CB1 agonists, and physical dependence in mice,^18−22^ thus strongly limiting the potential clinical development of irreversible inhibitors. Presently, some potent irreversible inhibitors are important milestones in the development history of MAGL inhibitors, for example, carbamate derivatives CAY10499,^23^ JZL-184,^9^ and ABX-1431.^24^ Some of the most representative reversible MAGL inhibitors are reported in Figure 1. The naturally occurring terpenoids pristimerin (Figure 1) and euphol (Figure 1) were discovered in 2009 as reversible MAGL inhibitors, although they are characterized by a scarce selectivity for MAGL.^25^ The piperazinyl azetidinyl amide ZYH (Figure 1) was patented by Janssen Pharmaceutica in 2010 and published in 2011 as a reversible MAGL inhibitor. The high-resolution X-ray crystal structure of the complex ZYH–human MAGL was reported, thus elucidating conformational changes of MAGL upon ligand binding.^26^ Benzo[*d*][1,3]dioxol-5-ylmethyl 6-phenylhexanoate **1** (Figure 1) is among the early discovered synthetic reversible MAGL inhibitors: compound **1** proved to be efficacious in an experimental autoimmune encephalomyelitis mouse model by reducing the symptoms of multiple sclerosis and delaying the clinical progression of the disease.^27^ Compounds 1,5-diphenylpyrazole-3-carboxamide **2** (Figure 1) and salicylketoxime derivative **3** (Figure 1) were developed by our research group: inhibitor **2** mitigated the neuropathic hypersensitivity induced *in vivo* by oxaliplatin,^28^ and compound **3** showed antiproliferative activity in a series of cancer cells.^29^ Piperazinyl pyrrolidin-2-one **4** (Figure 1) discovered by Takeda Pharmaceuticals was effective on the isolated enzyme, with inhibition values in the subnanomolar range, and also *in vivo*, where it decreased arachidonic acid concentration, thus increasing 2-AG levels in the mouse brain.^30^ Since 2014, the class of benzoylpiperidine-based MAGL inhibitors has been developed by our group and a series of hit-to-lead optimization processes have enabled the identification of nanomolar inhibitors **5a**–**c** (Figure 1),^31−35^ whose phenolic moiety is deeply located in the glycerol cavity of the enzyme, while their diaryl-sulfide extremity fits in the wide lipophilic channel of the enzyme normally hosting the unsaturated 2-AG chain.

## 2 Results and Discussion

### 2.1 Design of Benzylpiperidine Derivatives

**Figure 2:**
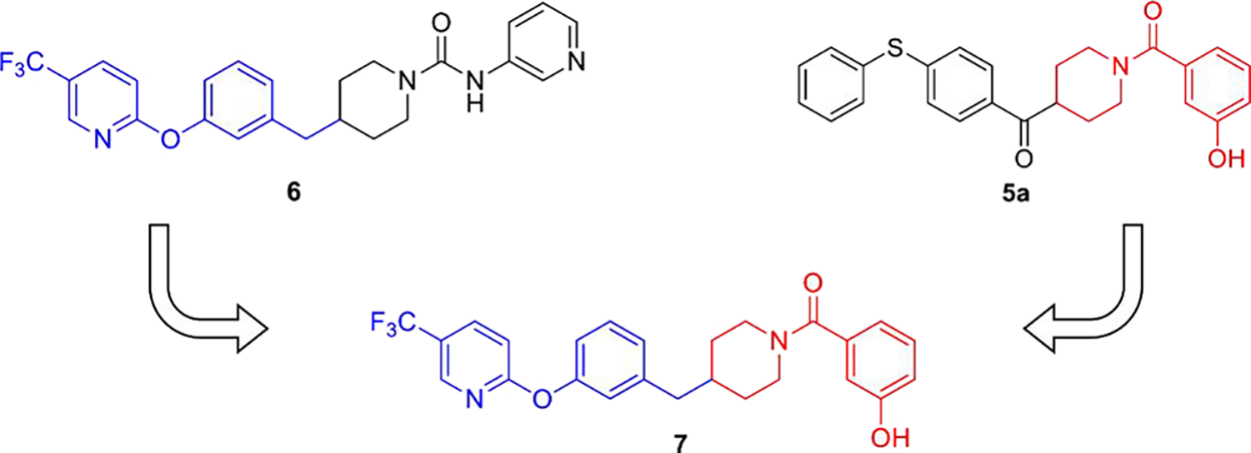
Design of the new benzylpiperidine derivative **7**. The moiety deriving from FAAH inhibitor **6** is highlighted in blue, and the moiety deriving from our MAGL inhibitor **5a** is highlighted in red.

Our starting point for the design of benzylpiperidine derivatives was the search for chemical moieties potentially able to fit in the MAGL active site through examination of the structures of different serine hydrolase inhibitors reported in the literature. Among them, our attention was attracted by the 2-(3-(piperidin-4-ylmethyl)phenoxy)-5-(trifluoromethyl)pyridine moiety of FAAH inhibitor PF-3845 (compound **6**, Figure 2) discovered in the Cravatt lab in 2009.^36^ This moiety could be considered to be somewhat similar to the benzoylpiperidine scaffold of our inhibitors (exemplified by compound **5a**, Figures 1 and 2) due to (a) the piperidine ring and (b) the presence of two aromatic rings (two phenyl rings in compound **5a** or phenyl and pyridine rings in compound **6**) connected by means of a linker (sulfur for compound **5a** or oxygen for compound **6**). Therefore, we envisioned to create the novel hybrid compound **7** (Figure 2) possessing on one side the 2-(3-(piperidin-4-ylmethyl)phenoxy)-5-(trifluoromethyl)pyridine moiety of compound **6** (in blue, Figure 2) and on the other side the amide phenolic moiety of compound **5a**, which proved to be fundamental for the MAGL inhibition activity, thus establishing a strategic hydrogen bond network with two active site residues, E53 and H272.^31^

**Figure 3:**
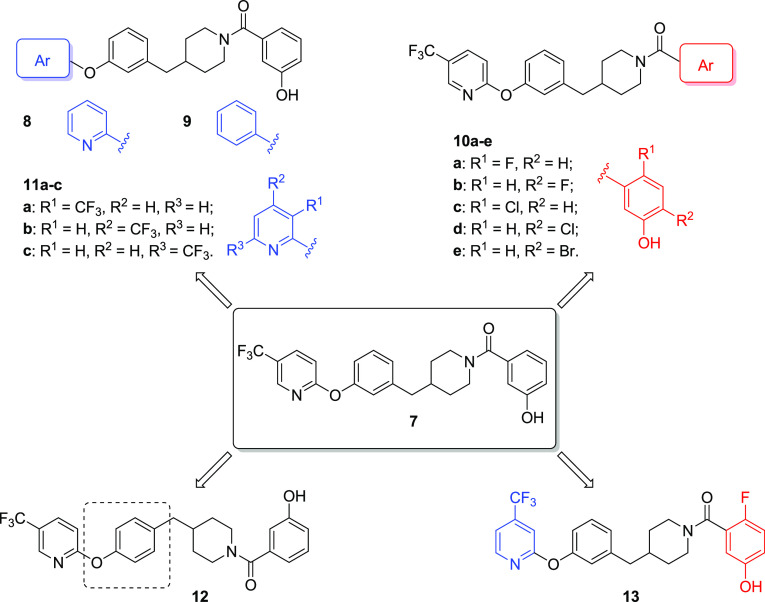
Newly synthesized benzylpiperidine derivatives **8**, **9**, **10a**–**e**, **11a**–**c**, **12**, and **13**. The modified moieties compared to parent compound **7** are highlighted in blue, in red, or with a dashed square.

As the second step of chemical exploration, compound **7** was modified to assess the importance of the different portions of this new scaffold. First, the trifluoromethyl group in position 5 of the terminal pyridine ring was removed, thus obtaining compound **8** (Figure 3), or shifted to different positions of the pyridine ring, such as in positions 3, 4, or 6, corresponding to compounds **11a**, **11b**, and **11c**, respectively (Figure 3). A further structural simplification was the conversion of the pyridine to a simple unsubstituted phenyl ring in compound **9** (Figure 3). Additionally, we inserted different halogen atoms on the phenolic ring of compound **7**; in particular, we based our decision on previously published halogenated benzoylpiperidine derivatives.^32^ Indeed, the presence of halogen atoms in specific positions of the benzoylpiperidine-based MAGL inhibitors published in 2019 allowed reaching IC_50_ values below 1 μM in enzymatic assays (compounds **11c**,**d** and **13b**–**d** of reference (32). In analogy with those MAGL inhibitors, we inserted: (a) the presence of fluorine or chlorine in the *para* position to the phenolic hydroxyl group (compound **10a** and **10c**, respectively, Figure 3); (b) the presence of fluorine or chlorine in the *para* position to the amide carbonyl group (compound **10b** and **10d**, respectively, Figure 3); and (c) the presence of a bromine atom in the *para* position to the amide carbonyl group (compound **10e**, Figure 3). The last modification consisted of connecting the pyridine ring to the rest of the molecule by a 1,4-disubstituted phenyl ring as in compound **12** (Figure 3), which replaced the 3-(piperidin-4-ylmethyl)phenoxy portion of compound **7**. Finally, compound **13** (Figure 3) simultaneously possesses a trifluoromethyl group in position 4 of the pyridine ring (as compound **11b**) and a 2-fluoro-5-hydroxyphenyl amide portion (as compound **10a**).

### 2.2 Chemistry

**Scheme 1:**
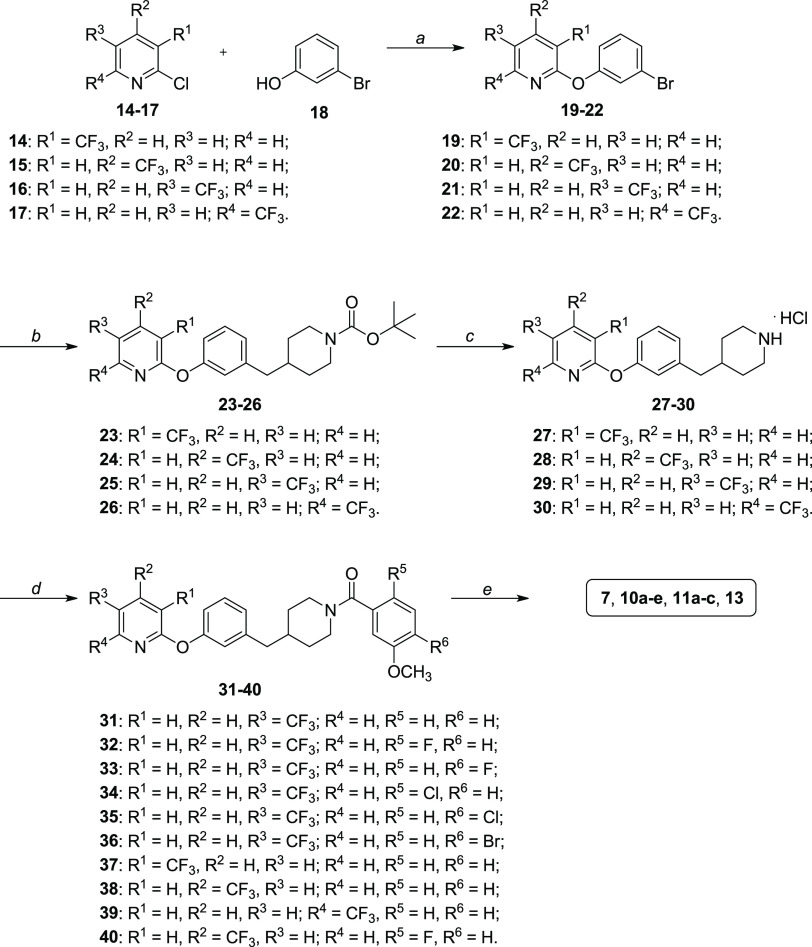
Synthesis of Compounds **7**, **10a**–**e**, **11a**–**c**, and **13** Reagents and conditions: (a) anhydrous K_2_CO_3_, anhydrous DMF, 110 °C, overnight [77–99%]; (b) i. *tert*-butyl 4-methylenepiperidine-1-carboxylate, 9-BBN 0.5 M solution in THF, anhydrous toluene, 115 °C, 1 h; ii. aq. 3.2 M NaOH, Pd(PPh_3_)_4_, TBAI, anhydrous toluene, 115 °C, 18 h [46–99%]; (c) HCl 4.0 M solution in dioxane, anhydrous MeOH, anhydrous CH_2_Cl_2_, RT, 1 h [99%]; (d) properly substituted benzoic acid, HATU, DIPEA, anhydrous DMF, RT, 3–12 h [41–75%]; (e) BBr_3_ 1 M solution in CH_2_Cl_2_, anhydrous CH_2_Cl_2_, −10 to 0 °C, then RT, 1–3 h [46–66%].

The synthesis of compounds **7**, **10a**–**e**, **11a**–**c**, and **13** follows a common synthetic procedure starting from the reaction of trifluoromethyl-substituted 2-chloropyridine **14–17** with 3-bromophenol **18** in the presence of potassium carbonate as the base and dry *N*,*N*-dimethylformamide (DMF) as the solvent (Scheme 1). Compounds **19–22** were subjected to a two-step reaction, which consisted first of a hydroboration of the alkene moiety of 1-Boc-4-methylenepiperidine by the hydroborating reagent 9-borabicyclo[3.3.1]nonane (9-BBN) followed by a cross coupling reaction with the brominated derivatives **19–22** with NaOH as the base, Pd(PPh_3_)_4_ as the catalytic system, and tetrabutylammonium iodide as the phase-transfer catalyst in toluene to assemble the central benzylpiperidine scaffold of these compounds (Scheme 1). *N*-Boc-protected intermediates **23–26** were deprotected by using a solution of HCl in dioxane. The corresponding piperidine hydrochlorides **27–30** were reacted with the properly substituted benzoic acids, which are 3-methoxybenzoic acid for compounds **31** and **37–39**, 2-fluoro-5-methoxybenzoic acid for compounds **32** and **40**, 4-fluoro-3-methoxybenzoic acid for compound **33**, 2-chloro-5-methoxybenzoic acid for compound **34**, 4-chloro-3-methoxybenzoic acid for compound **35**, and 4-bromo-5-methoxybenzoic acid for compound **36**, in the presence of 1-[bis(dimethylamino)methylene]-1*H*-1,2,3-triazolo[4,5-*b*]pyridinium 3-oxide hexafluorophosphate (HATU) as the condensing agent, *N*,*N*-diisopropylethylamine (DIPEA) as the base, and dry DMF as the solvent, as previously reported (Scheme 1).^32^ Deprotection of the methoxy group by boron tribromide in dichloromethane furnished the final hydroxy-substituted compounds.

**Scheme 2:**
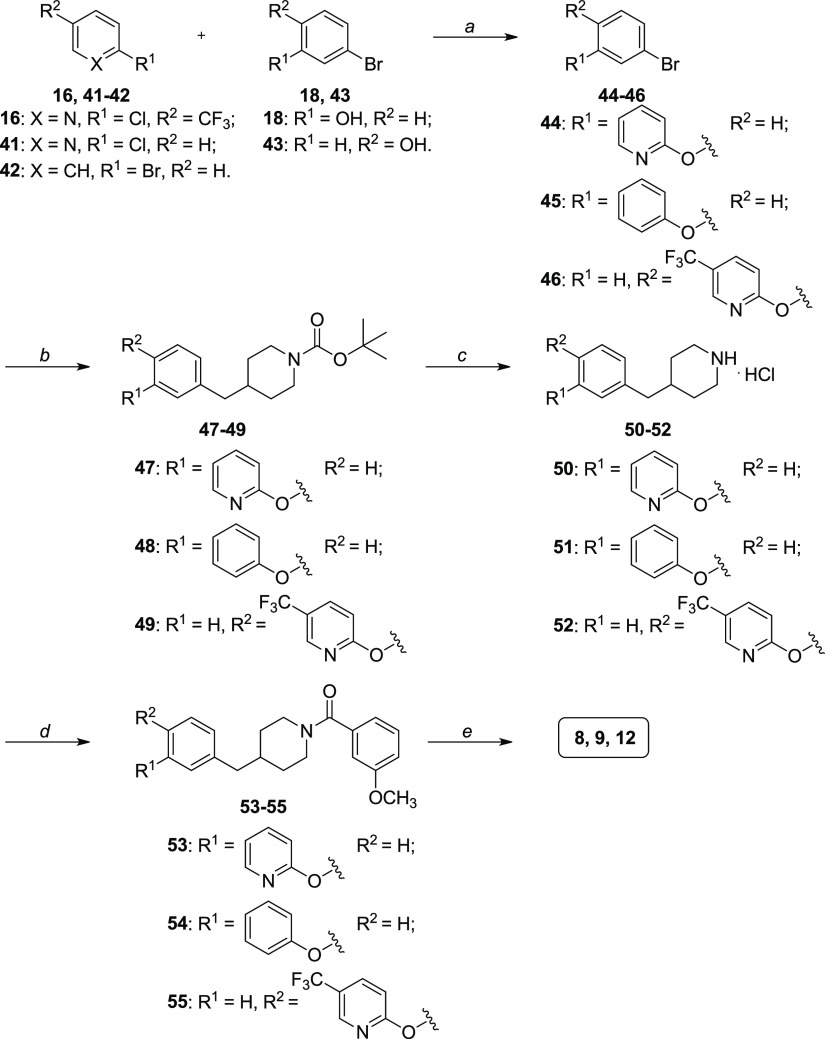
Synthesis of Compounds **8**, **9**, and **12** Reagents and conditions: (a) for compounds **41** and **42**: K_3_PO_4_, CuI, anhydrous DMSO, 130 °C, 24 h [11–32%]; for compound **16**: anhydrous K_2_CO_3_, anhydrous DMF, 110 °C, overnight [84%]; (b) i. *tert*-butyl 4-methylenepiperidine-1-carboxylate, 9-BBN 0.5 M solution in THF, anhydrous toluene, 115 °C, 1 h; ii. aq. 3.2 M NaOH, Pd(PPh_3_)_4_, TBAI, anhydrous toluene, 115 °C, 18 h [30–78%]; (c) HCl 4.0 M solution in dioxane, anhydrous MeOH, anhydrous CH_2_Cl_2_, RT, 1 h [90–99%]; (d) 3-methoxybenzoic acid, HATU, DIPEA, anhydrous DMF, RT, 3–12 h [53–72%]; (e) BBr_3_ 1 M solution in CH_2_Cl_2_, anhydrous CH_2_Cl_2_, −10 to 0 °C, then RT, 1–3 h [29–62%].

A nearly identical synthetic strategy was adopted for the preparation of compounds **8**, **9**, and **12** (Scheme 2). The only exception is the formation of compounds **44** and **45** by an “Ullmann-type″ reaction that was a copper-catalyzed nucleophilic aromatic substitution between 2-chloropyridine **41** or bromobenzene **42** and 3-bromophenol **18**, in the presence of potassium phosphate as the base and anhydrous DMSO as the solvent. This reaction afforded compounds **44** and **45** in low to moderate yields.

The use of 4-bromophenol **43** instead of 3-bromophenol **18** in the first step (step a, Scheme 2) allowed the formation of the different central scaffold bearing the 1,4-disubstituted phenyl ring of the final compound **12** (Scheme 2).

### 2.3 Enzymatic Assays

The herein reported compounds were tested for their inhibition activity on human MAGL by adopting a spectrophotometric method, which uses 4-nitrophenylacetate as the substrate (Table 1).^33^ All the compounds were also evaluated for their inhibition activity on human FAAH, to determine their selectivity, since they all derive from a structural optimization of a fragment belonging to a FAAH inhibitor (Table 1).^36^ The enzymatic method used for FAAH assays was similar to that used for MAGL, differing in the used substrate, which is in this case 7-amino-4-methyl coumarin-arachidonamide.^31^ The inhibition potencies of the newly synthesized derivatives were compared to the previously published benzoylpiperidine MAGL inhibitors **5a** and **5b**.^34^

**Table 1:**
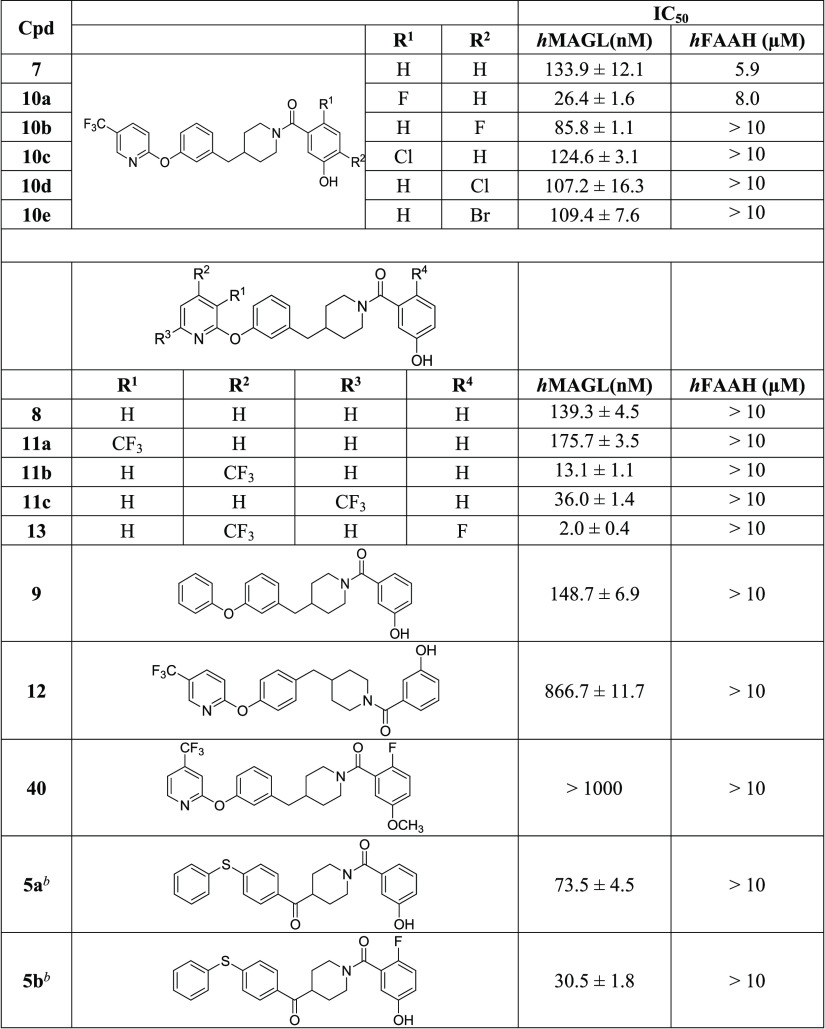
*In Vitro* Inhibitory Activity on Human MAGL and FAAH (*h*MAGL and *h*FAAH, IC_50_, nM)^*a*^ of Derivatives **7–9**, **10a**–**e**, **11a**–**c**, **12**, **13**, and **40**

a Enzymatic values are the mean of three or more independent experiments, performed in duplicate.

b Ref (34).

Compound **7**, which represents the first synthesized benzylpiperidine MAGL inhibitor, showed encouraging results, possessing an IC_50_ value on MAGL of 133.9 nM and a good selectivity over FAAH (IC_50_ = 5.9 μM). The insertion of chlorine atoms on the phenolic ring, as in compounds **10c** and **10d**, approximately maintains or slightly improves the inhibition activity (IC_50_ values of 124.6 and 107.2 nM for **10c** and **10d**, respectively); likewise, the presence of the bromine in compound **10e** did not significantly modify the inhibition potency (IC_50_ = 109.4 nM). On the contrary, when a fluorine atom was introduced in the phenolic ring (compounds **10a** and **10b**), the inhibition activity improves 1.6-fold in the case of **10b** or 5-fold in the case of **10a**, compared to initial compound **7**; thus, compound **10a** reaches an excellent IC_50_ value of 26.4 nM while still maintaining a good selectivity over FAAH (IC_50_ = 8.0 μM). Scarce results were provided by the drastic change of the scaffold: the removal of the trifluoromethyl group in compound **8** leads to a maintenance of the inhibition activity shown by derivative **7** (for compound **8**: IC_50_ = 139.3 nM), the substitution of the 5-trifluoromethylpyirdine with a simple benzene ring in compound **9** leads to a slight increase of the IC_50_ value (148.7 nM), and the worst result concerns compound **12**, which dramatically loses potency, showing an IC_50_ value of 866.7 nM. The shift of the trifluoromethyl group to position 3 of pyridine was detrimental to the activity since a decrease of potency is evident for compound **11a**, with an IC_50_ value of 175.7 nM. Differently, when the CF_3_ moiety was moved to position 6, the activity improves: compound **11c** displays an IC_50_ value of 36.0 nM. The best result was achieved when CF_3_ is in position 4 of the pyridine ring in compound **11b**, which determines a 10-fold improvement in the IC_50_ value (IC_50_ = 13.1 nM) for MAGL and an enhanced selectivity over FAAH (IC_50_ greater than 10 μM). The combination of the best structural modifications of compound **7**, i.e., a fluorine atom in the *para* position to the phenolic hydroxyl group (as in compound **10a**) and the change of position of the trifluoromethyl group from 5 to 4 of the pyridine ring (as in compound **11b**), gave rise to compound **13** in which both the modifications exert a synergistic effect on the inhibition activity: **13** shows an IC_50_ value of 2.0 nM; therefore, it represents the most active MAGL inhibitor of this class but still was endowed with a high degree of selectivity over FAAH (IC_50_ > 10 μM). Moreover, **13** overtakes benzoylpiperidine **5b** in terms of inhibition potency (IC_50_ = 30.5 nM), which is used as the reference compound.^34^ With the aim of confirming the importance of the phenolic OH moiety for the interaction with MAGL, the methoxylated counterpart of compound **13** (compound **40**) was tested, and as expected it proved to be inactive (IC_50_ greater than 1 μM).

**Figure 4:**
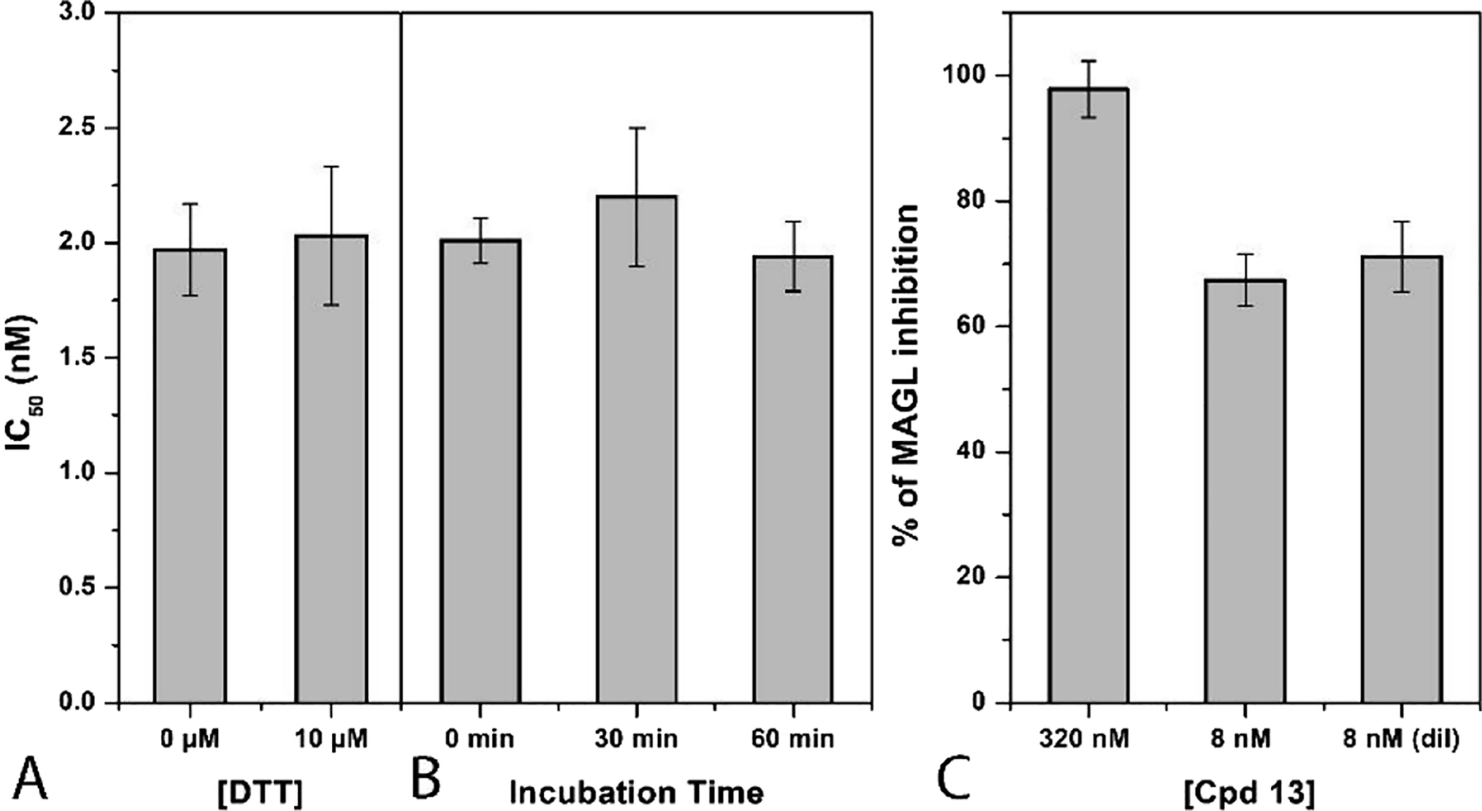
Analysis of the mechanism of MAGL inhibition of compound **13**. (A) Effect of DTT on MAGL inhibition activity. (B) IC_50_ (nM) values at different preincubation times with MAGL (0, 30, and 60 min). (C) Dilution assay: the first two columns indicate the inhibition percentage of the compound at concentrations of 320 and 8 nM. The third column indicates the inhibition percentage of the compound after dilution (final concentration = 8 nM).

To verify whether these compounds could interact with cysteine residues of MAGL, the activity of **13** was also tested in the presence of the thiol-containing agent 1,4-dithio-dl-threitol (DTT). As shown in Figure 4A, the IC_50_ values of the compound were not significantly influenced by the presence of DTT, thus excluding any significant interaction of compound **13** with MAGL cysteine residues. Furthermore, to confirm the reversible inhibition mechanism, compounds **13** was also subjected to pre-incubation and dilution assays. As shown in Figure 4B, the test suggests a reversible binding mode, as the compound showed very similar activities at all the three different incubation times. As a second test, we investigated the effect of dilution on the inhibition activity. The inhibition produced by incubation with a concentration of 320 nM compound **13** was compared with the inhibition produced by a 40× dilution, and as shown in Figure 4C, the inhibition produced at a concentration of 320 nM was significantly higher compared with that observed at a 40× dilution, comparable to the effect produced by an 8 nM concentration of the compound, thus clearly supporting a reversible mechanism of inhibition. Reference compound JZL-184 was also subjected to the same experimental assays, and as shown in Figure S44, the results confirmed its irreversible mechanism of action with the absence of interactions with DTT.

The mode of inhibition of compound **13** was then evaluated by measuring the Michaelis–Menten kinetics at various inhibitor concentrations. The datasets were plotted as substrate concentration versus enzyme activity and analyzed by applying the mixed model inhibition fit. Kinetic studies indicate for compound **13** an α value greater than 10,000, thus supporting the competitive behavior for this compound, and a measured *K*_i_ value of 1.42 ± 0.16 nM (see Figure S45).

### 2.4 Selectivity Assays

Beyond enzymatic assays on FAAH (Section 2.3.), compound **13** was also profiled for its selectivity toward CB1 and CB2 and it did not significantly bind to any receptor, resulting in IC_50_ values greater than 10 μM (see Table S4). Then, with the aim to assess the selectivity of **13** in a broader context of the serine hydrolase family, we performed competitive activity-based protein profiling (ABPP) experiments using mouse brain membrane preparations. ABPP is a functional proteomic technology that exploits chemical probes that react with mechanistically related classes of enzymes.^37^ TAMRA-fluorophosphonate (TAMRA-FP) is used to visualize serine hydrolases, which include the major eCBs degrading enzymes.^38^ An important advantage of ABPP relative to other approaches is that it can detect changes in the activity of very low-abundance enzymes in highly complex samples and can simultaneously assess the potency and selectivity of an inhibitor toward the entire family of serine hydrolases in a specific tissue.

**Figure 5:**
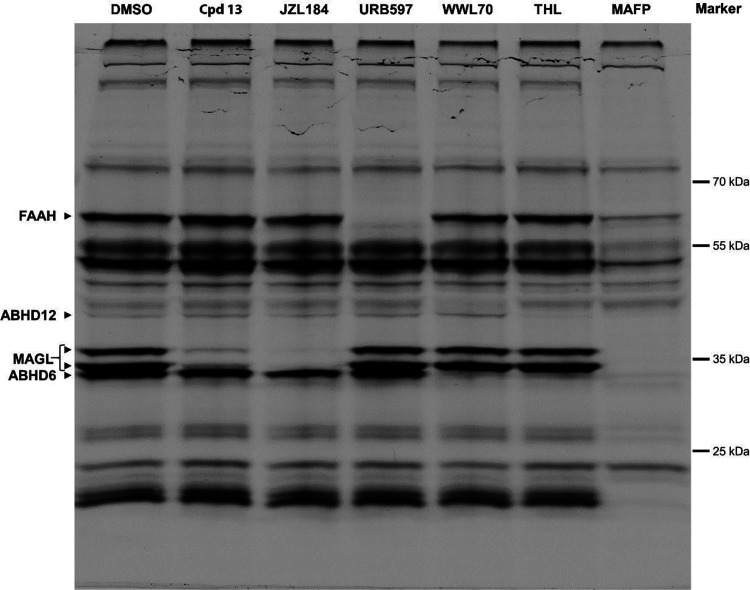
ABPP with fluorescent labeling of serine hydrolases in mouse brain membrane homogenates using a TAMRA-FP serine hydrolase probe and different inhibitors as controls. The mouse brain membranes (4 mg/mL) were pre-incubated for 25 min with either DMSO, **13** (10 μM, MAGL inhibitor), JZL-184 (10 μM, MAGL inhibitor),^9^ URB597 (4 μM, FAAH inhibitor),^39^ WWL70 (10 μM, ABHD6 inhibitor),^40^ THL (30 μM, ABHD6 and ABHD12 inhibitor),^41^ or MAFP (5 μM, unselective serine hydrolase inhibitor).^42^ After additional incubation with TAMRA-FP (125 nM) for 25 min, the samples were separated in SDS-PAGE. A representative image of the TAMRA-FP signal after SDS-PAGE is shown. The presented results could be observed in three independent experiments.

Mouse brain membranes were pre-incubated with control (DMSO), compound **13**, and other known inhibitors of serine hydrolases such as JZL-184 (MAGL inhibitor),^9^ URB597 (FAAH inhibitor),^39^ WWL70 (ABHD6 inhibitor),^40^ THL (ABHD6 and ABHD12 inhibitor),^41^ and MAFP (unselective serine hydrolase inhibitor)^42^ as controls. The TAMRA-FP signal after SDS-PAGE highlighted that compound **13** at a concentration of 10 μM selectively inhibited MAGL (see the two bands associated with MAGL^43^ in Figure 5), without affecting other serine hydrolases such as FAAH, ABHD6, and ABHD12, similar to reference MAGL inhibitor JZL-184. Since TAMRA-FP is a highly potent covalent irreversible probe, in this ABPP assay, a reversible inhibitor cannot completely compete with it, despite the high inhibition potency. On the other hand, the covalent irreversible inhibitor JZL-184 can fully compete with the probe. The serine hydrolase bands associated with other enzymes disappeared according to the tested control inhibitor (Figure 5): the FAAH band for URB597, the ABHD6 band for WWL70 and THL, and the ABHD12 band for THL. In the case of pre-treatment with MAFP, all the bands relative to FAAH, MAGL, ABHD6, and ABHD12 disappeared (Figure 5).

### 2.5 Molecular Modeling Studies

**Figure 6:**
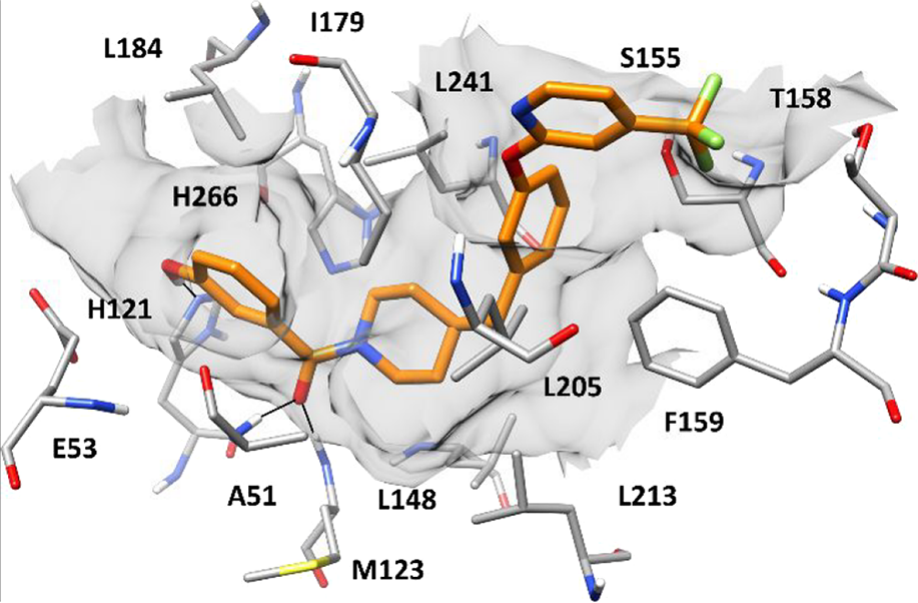
Minimized average structure of *h*MAGL in complex with compound **11b** in the predicted binding pose. The protein residues surrounding the ligand are shown. Ligand–protein hydrogen bonds are highlighted with black lines. The inner surface of the protein binding site is shown in gray (PDB code 5ZUN).

To rationalize how this series of inhibitors could interact with MAGL into its binding site, molecular modeling studies were carried out using compound **11b** as a reference. The ligand was docked into the crystal structure of MAGL (PDB code 5ZUN) using a robust protocol based on AUTODOCK4 software. The eight potential MAGL-**11b** complexes predicted by the protocol were then subjected to 1.05 μs of molecular dynamics (MD) simulations and analyzed in terms of RMSD of the ligand disposition during the MD as well as ligand–protein binding free energy evaluations based on the molecular mechanics Poisson–Boltzmann surface area (MM-PBSA) method (see the Experimental Section for details). The results highlighted binding pose 3 as the most reliable, being the only one associated with an average ligand RMSD below 2.0 Å (Table S1) and corresponding to an interaction energy (ΔPBSA = −8.9 kcal/mol) at least 3.6 kcal/mol higher than those estimated for all other binding poses (Table S2). Figure 6 shows the energy-minimized average structure of MAGL complexed with compound **11b**, in the proposed binding mode, obtained from the last 500 ns of MD simulation. The ligand presents a sort of L-shaped binding conformation bent at the level of the methylene linker connecting the two main structural portions of the molecule, in which the benzoylpiperidine moiety occupies the central region of MAGL catalytic site, while the phenoxypyridine fragment is placed at the entrance of the binding cavity, thus closing its access. Due to the absence of a central carbonyl group in the ligand able to interact with the oxyanion hole residues A51 and M123, as observed in the parent MAGL inhibitor **5b**,^35^ the disposition of compound **11b** is shifted toward the entrance of the binding site (Figure S46). Nevertheless, the ligand still establishes the key H-bond interactions with A51 and M123, maintained for more than 90% of the MD simulation, through its benzoyl oxygen. Moreover, the phenolic OH group forms a strong H-bond with H121 that is observed for the whole simulation and thus contributes to firmly anchoring the ligand to the enzyme binding site. Finally, the phenol moiety shows hydrophobic contacts with A51, L184, and H266, while the adjacent piperidine fragment forms lipophilic interactions with the side chains of L148, I179, L213, and L241. The phenyl ring belonging to the phenoxypyridine moiety of **11b** fits well the rather narrow entrance of the MAGL catalytic site delimited by S155, F159, I179, L205, and L241; in fact, the phenyl ring shows extensive hydrophobic interactions with I179, L205, and L241 as well as a partial T-shaped stacking with F159. Finally, the trifluoropyridine moiety of **11b** is placed in a solvent-exposed area adjacent to the entrance of the binding pocket and MAGL lid domain. In particular, the trifluoromethyl group is placed in a small amphiphilic pocket delimited by S155, T158, and F159, forming van der Waals interactions with these residues, while the pyridine ring shows hydrophobic contacts predominantly with I179. These interactions significantly stabilize the orientation of the trifluoromethyl fragment and thus the whole binding mode of the inhibitor, also limiting its exposure to the solvent.

**Figure 7:**
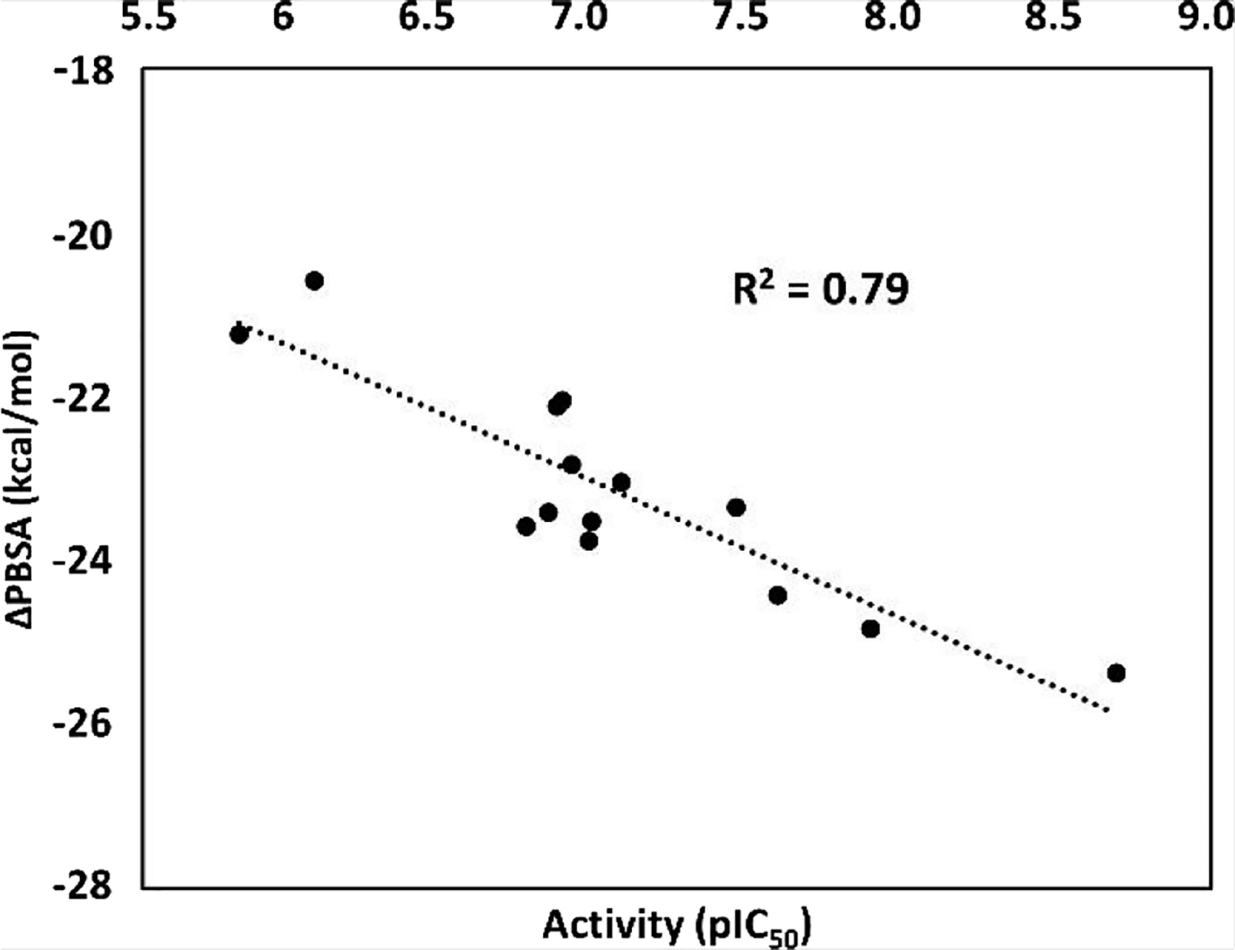
Correlation between the compound’s activities expressed as pIC_50_ values and the binding energies estimated using the best MM-PBSA protocol (ε_int_ = 4) expressed in kcal/mol.

The minimized average structure of MAGL bound to **11b** was then used as a reference for predicting the binding mode of the other compounds of the series, namely, **7–9**, **10a**–**e**, **11a**, **11c**, **12**, **13**, and **40**, which were subjected to an analogous docking/MD protocol. In addition, ligand–protein binding free energy evaluations were performed using the MM-PBSA approach based on the results of the MD simulations obtained for each MAGL–ligand complex, looking for a correlation between the binding energies estimated for the ligands and their corresponding enzymatic activity that could confirm the reliability of the computational protocol and help interpret the SAR data derived from the MAGL inhibition assays. Considering the significant level of polarizability of the key phenolic moiety of the analyzed ligands, as well as of MAGL binding site, due to the presence of charged residues such as E53 within the inner portion of the catalytic site, various MM-PBSA protocols differing for the internal dielectric constant (ε_int_) value were tested with the aim of identifying the most suitable one for correlating binding energies with activities.^44^Figure 7 shows the correlation obtained between the compounds activities and the binding energies estimated using the best MM-PBSA protocol evaluated, obtained using ε_int_ = 4, which showed a squared correlation coefficient of 0.79 (see also Tables S3 and S4).

The results obtained confirmed the reliability of the binding modes predicted for the series of ligands and helped us decipher the SAR data obtained from the experimental assays. Compound **7**, bearing the trifluoromethyl group in position 5 of the pyridine ring, showed a reduced activity with respect to compound **11b** and was associated with a 2.7 kcal/mol lower binding free energy. As shown in Figure S1A, the ligand assumes a binding mode very similar to that predicted for **11b**, but the trifluoromethyl group is placed outside the amphiphilic pocket formed by S155, F159, I179, L205, and L241, thus losing the interaction with these residues and remaining fully exposed to the solvent. These features may explain the reduced activity of compound **7** compared to **11b**. The same considerations are also valid for compound **8**, which does not show any interactions with the residues of the amphiphilic pocket because it lacks the terminal trifluoromethyl group. In fact, the ligand shows a MAGL inhibitory activity comparable to **7** and was also associated with the same binding free energy (Table S4). In line with these considerations, the significant drop of activity with respect to **11b** observed in compound **12**, for which one of the lowest binding energies was estimated (Table S4), could be determined by the complete loss of interactions of its trifluoromethylpyridine moiety, which is connected differently to the rest of the molecule with respect to the other derivatives. Due to this structural change, the trifluoromethylpyridine fragment of the compound is placed in the middle of the solvent-exposed region of the binding site entrance, completely outside the catalytic pocket (Figure S1B); therefore, the ligand loses not only the interactions of the trifluoromethyl group with the residues of the amphiphilic pocket but also the lipophilic contacts between the pyridine ring and I179 as well as the partial T-shaped stacking formed by the adjacent ligand phenyl ring with F159. The destabilizing effect of these features in the binding mode of compound **12** is highlighted by the RMSD of the ligand during the MD, which shows an average value around 3 Å due to the high mobility of the trifluoromethylpyridine moiety. Finally, the highest binding free energy was correctly estimated for compound **13** (Table S4), bearing a fluorine in the *para* position to the OH group of the phenol ring, for which a binding mode fully comparable with that of **11b** was predicted (Figure S47). The fluorine atom is supposed to increase the polarization of the ligand hydroxyl group, thus boosting the strength of the H-bond formed with H121, similarly to what was observed for the series of benzoylpiperidines to which the parent compound **5b** belongs. In agreement with this hypothesis, the increase in binding free energy observed for compound **13** with respect to **11b** seems to be due to more favorable polar energetic terms and, in particular, to stronger ligand–protein electrostatic interactions (Table S6). On the contrary, the methylation of the OH group prevents the formation of such H-bonds, thus determining the dramatic drop of activity of compound **40**, for which one of the lowest binding energies was calculated.

### 2.6 Biological Studies in Pancreatic Cancer Cells

#### 2.6.1 MAGL Expression in Pancreatic Cancer Cells

**Figure 8:**
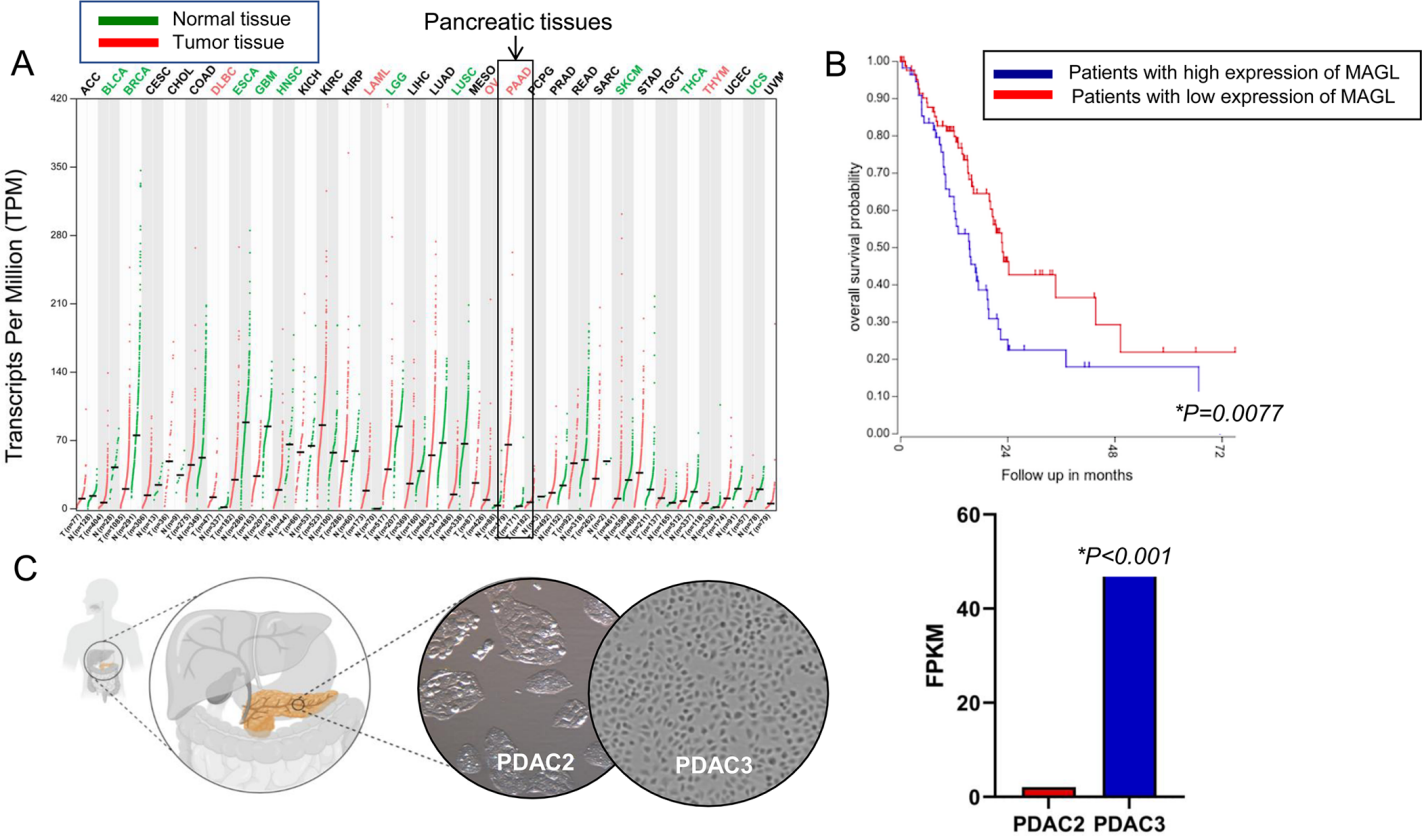
MAGL gene expression levels. (A) MAGL mRNA is more expressed in cancer tissues than in normal tissues (http://gepia.cancer-pku.cn/detail.php?gene=MAGL). Pancreatic cancer tissues are among the tumor tissues with the highest expression levels of MAGL. (B) MAGL mRNA expression is a prognostic factor in pancreatic cancer. The expression cutoff between patients with high versus low expression of MAGL (5132, RNA expression units) was obtained by the “R2: Genomics Analysis and Visualization Platform”. (C) Two primary pancreatic cancer cell cultures (PDAC2 and PDAC3) originating from patients undergoing surgery for pancreatic cancer showed significantly different expression levels of MAGL mRNA.

MAGL is overexpressed in different tumor types, including pancreatic adenocarcinoma (PAAD) as demonstrated by the analysis of RNA sequencing expression data of 179 pancreatic tumors and 171 normal pancreatic samples from the TCGA and GTEx projects^45^ as reported in Figure 8A. Applying the online genomics and visualization platform R2 (http://r2.amc.nl) on the pancreatic adenocarcinoma TCGA dataset (178-rsem-tcgars), 57 patients were classified with high MAGL mRNA expression and 89 with a low MAGL mRNA expression, while 32 samples were excluded due to missing survival data. In the computed Kaplan–Meier curve (Figure 8B), it is shown that a high MAGL mRNA level is significantly (*p* = 0.007) correlated with a poor overall survival probability compared to a low expression.

Our next-generation RNA sequencing (NGS) data showed that MAGL mRNA is expressed in two primary pancreatic cancer cell cultures, with the highest fragments per kilobase million (FPKM) score in PDAC3 (pancreatic ductal adenocarcinoma) cells (Figure 8C), which originated from the most clinically aggressive tumor.^46^ Analyzing our transcriptomic data, we found out that PDAC3 cells have a significantly higher expression of arsenite-resistance protein 2 (ARS2), a zinc finger protein that is essential for early mammalian development. Of note, a recent study in glioblastoma models demonstrated that a number of pro-tumorigenic genes are potentially regulated at the transcriptional level by ARS2 and specifically identified MAGL as a novel target of ARS2.^47^ This might at least in part explain MAGL differential expression and its impact on cancer aggressiveness. However, the role of ARS2 in the pathophysiology of pancreatic cancer has still to be clarified.

#### 2.6.2 Antiproliferative Activity and Effects on Induction of Apoptosis

The most active compound of this series of benzylpiperidine derivatives, inhibitor **13**, was tested in antiproliferative activity assays on different pancreatic ductal adenocarcinoma cancer cells, including the SUIT-2 immortalized cell line, PDAC2 and PDAC3 primary cell cultures, and immortalized ductal normal cells HPNE, by sulforhodamine-B (SRB) assay. PDAC2 and PDAC3 cell cultures were selected as cellular models in most of the following experiments because they maintain the same metastatic, genetic, and histopathological features of the primary tumor.

**Figure 9:**
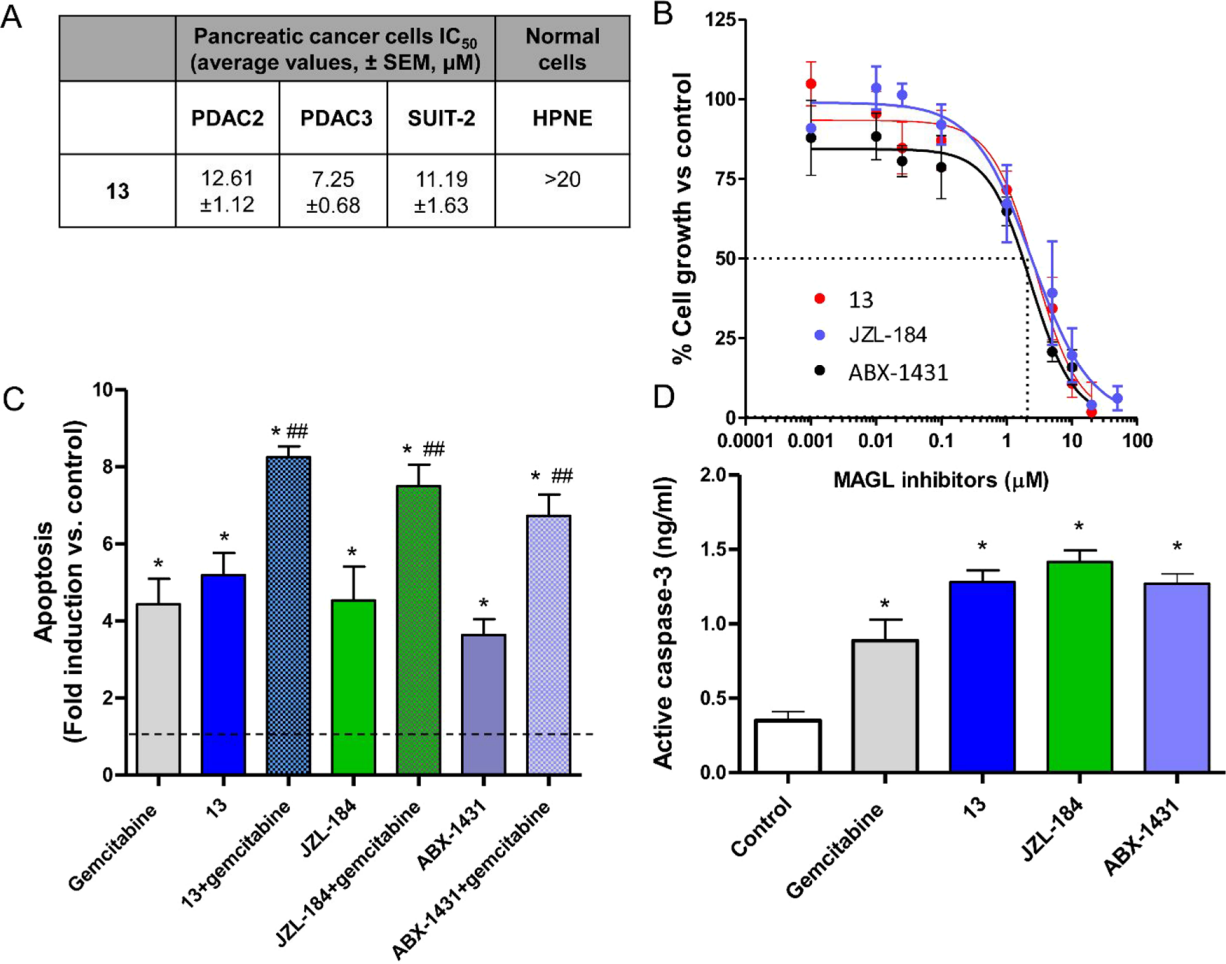
Antiproliferative and pro-apoptotic effects of MAGL inhibitor **13**. (A) IC_50_ of compound **13** in different pancreatic cancer models and in the immortalized ductal cells HPNE. (B) Representative curves of PDAC3 cells growth inhibitory effects of **13**, JZL-184 and ABX-1431, as control. (C) Induction of apoptosis and (D) levels of active caspase-3 in PDAC3 cells treated with **13**, gemcitabine, JZL-184, and ABX-1431 for 72 h, compared to control/untreated cells (value = 1, as illustrated by the dashed line). Measurements were performed in triplicate, and data are presented as means ± SEM. *p < 0.05 versus control; #*p* < 0.05 versus gemcitabine.

PDAC2, PDAC3, and SUIT-2 cells showed different sensitivities to compound **13** (Figure 9A). Both SUIT-2 and PDAC2 cells, despite being immortalized and primary cells, show a similar sensitivity. More specifically, the IC_50_ values of compound **13** in the antiproliferative activity assays with SUIT-2 and PDAC2 cells were 11.19 and 12.61 μM, respectively. However, PDAC3 cells were slightly more sensitive to MAGL inhibitor **13** with an IC_50_ value of 7.25 μM, and this difference could be in part explained considering the higher MAGL mRNA overexpression in PDAC3 compared to PDAC2 primary cell culture. Of note, the immortalized pancreatic ductal normal cells were not sensitive to compound **13**. Reference MAGL inhibitors JZL-184 and ABX-1431 were effective in reducing PDAC3 proliferation, as demonstrated by cell growth curves (Figure 9B). Previous studies suggested that inhibition of MAGL might increase apoptosis and tumor cell sensitivity to chemotherapy.^35,48^ Therefore, we evaluated apoptosis induction by **13** using two different assays and compared the pro-apoptotic effects of **13** with gemcitabine, a drug used for the standard treatment of pancreatic cancer (which has IC_50_ values in the nanomolar range, as reported in our previous studies^34^), and with the two reference MAGL inhibitors, JZL-184 and ABX-1431. In particular, the Annexin-V staining showed that **13** strongly enhanced apoptosis induction in PDAC3 cells (Figure 9C). Remarkably, this compound was able to significantly increase apoptosis induction compared to untreated cells. Gemcitabine has a similar effect and the combination led to an addictive effect. Similar results were observed in PDAC2 cells (i.e., apoptosis fold induction/change of approximately 4, 5, and 9 after treatment with **13**, gemcitabine and their combination). JZL-184 and ABX-1431 and their relative combinations with gemcitabine induced apoptosis in PDAC3 cells with a similar or slightly lower potency compared to that of **13** (Figure 9C). Moreover, we demonstrated that the apoptotic response of both PDAC3 and PDAC2 cells after exposure to compound **13** was associated with the concomitant stimulation of caspase-3 (Figure 9D). Indeed, our immunoassay measured significantly higher levels of active caspase-3 in PDAC3 cells treated with **13** or gemcitabine compared to untreated cells. PDAC2 cells showed similar results with slightly lower levels of active caspase-3 (0.35, 0.88, and 1.12 ng/mL in untreated, gemcitabine-treated, and **13**-treated cells, respectively). Even in this case, JZL-184 and ABX-1431 were similarly effective in stimulating caspase-3 in PDAC3 cells (Figure 9D).

#### 2.6.3 Cell Migration Assays

**Figure 10:**
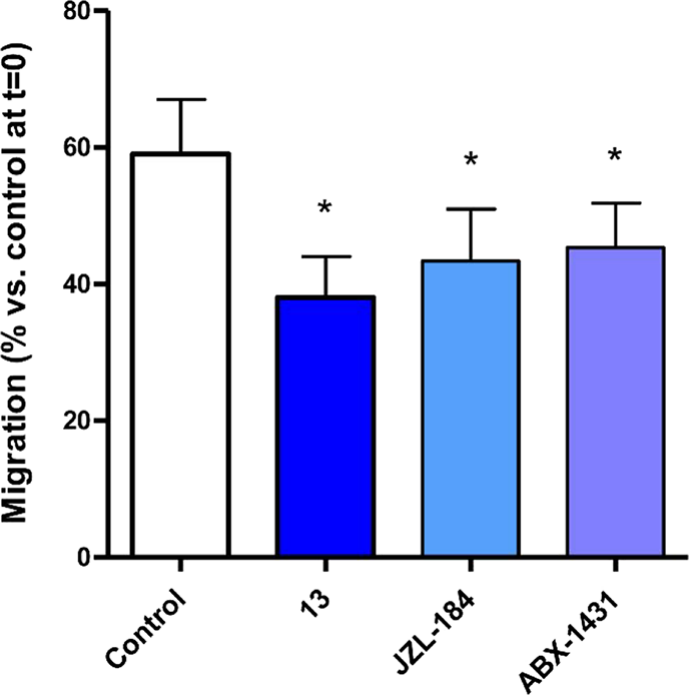
Antimigratory effects of MAGL inhibitors. Statistical evaluation of the results of the wound-healing/migration assay on the PDAC3 cells 20 h after scratch induction and treatment. The percentages of scratch closure for control, **13**-, JZL-184-, or ABX-1431-treated cells were compared with one-way analysis of variance (ANOVA)/*t* test. *p < 0.05 versus control.

It is well known that the early metastatic behavior of PDAC is responsible for the poor prognosis of this tumor. Therefore, new therapeutic agents are needed to overcome PDAC aggressiveness and counteract PDAC metastasis. The effect of compound **13** on cell migration was investigated using the wound-healing assay and compared with two reference MAGL inhibitors, JZL-184 and ABX-1431. In the PDAC3 cells (Figure 10), 59 ± 8% of the scratch area was closed after 20 h when treated with 0.1% DMSO (control). After treatment with JZL-184 or ABX-1431, 43 ± 8% or 45 ± 7% of the scratch was closed, respectively, while **13** induced a significant reduction of migration, with 38 ± 6% of the scratch closed (Figure 10). In PDAC2, the control showed 70 ± 3% gap closure, and in these cells, **13** treatment resulted in a closure with 61 ± 3%.

#### 2.6.4 Synergistic Interaction of Compound **13** with Gemcitabine and Potential Mechanisms Underlying Its Effects on Apoptosis, Migration, and Potentiation of Gemcitabine Activity

**Figure 11:**
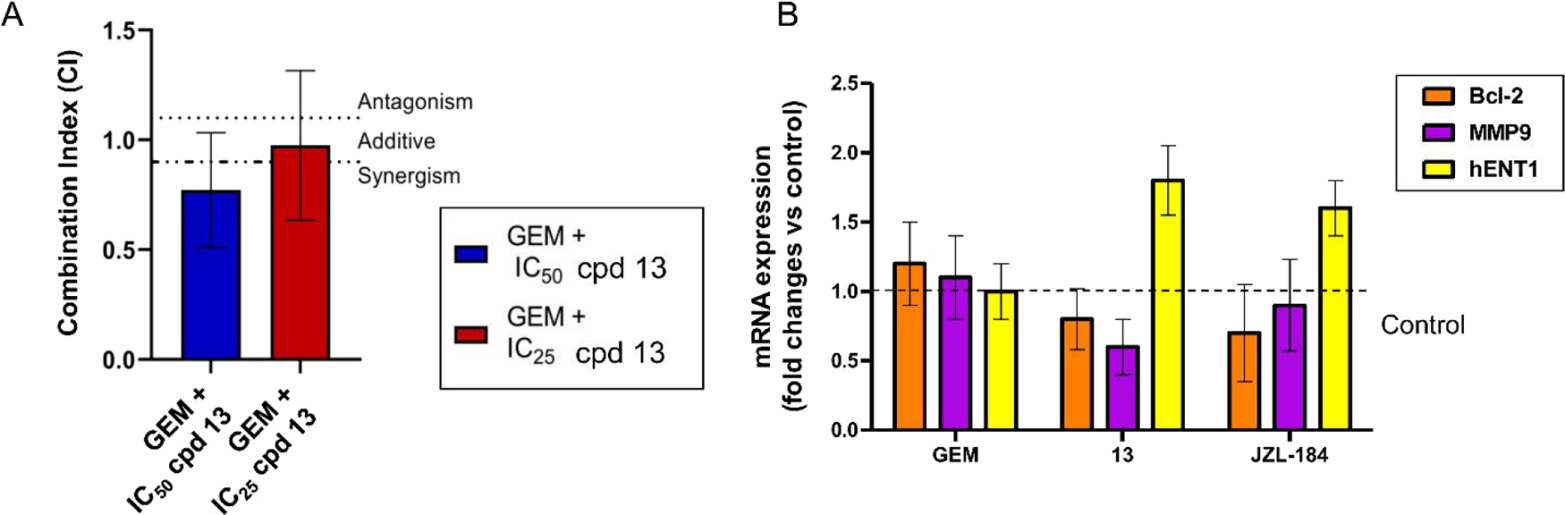
Combination assay and modulation of gene expression. (A) CI values of gemcitabine (GEM) combined with compound **13** at IC_50_ and IC_25_. The upper line represents an antagonistic CI > 1.2, the lower bar represents a synergistic CI < 0.8. (B) Combined results of different PCR experiments, evaluating the effect of GEM, **13**, and JZL-184 on potential determinants of apoptosis induction, migration, and synergistic interaction with gemcitabine compared to control/untreated cells (value = 1, as illustrated by the dashed line). Measurements were performed in triplicate, and data are presented as means ± SEM.

The pharmacological interaction of the MAGL inhibitor **13** and gemcitabine was determined on PDAC3 cells (Figure 11A) using fixed concentrations of compound **13** corresponding to its IC_25_ or IC_50_ value together with a 0–1.25 μM concentration range of gemcitabine. The PDAC3 cells treated with gemcitabine and compound **13** at IC_50_ showed synergy, whereas compound **13** at IC_25_ was additive. The PDAC2 cells showed a slight synergy between gemcitabine and **13** at IC_50_ (CI, 0.76) and additive interaction with **13** at IC_25_ (CI, 0.96).

To elucidate the previous data on apoptosis and migration and the mechanism of interaction, further studies focused on several potential cellular determinants and effectors of drug activities, such as the anti-apoptotic factor Bcl-2, the key matrix metalloproteinase 9 (MMP9), which promotes cell migration, and the main gemcitabine transporter, the human equilibrative nucleoside transporter 1 (*h*ENT1). As shown in Figure 11B, PCR analyses demonstrated the modulation of several of these important factors by compound **13**. In particular, **13** induced a slight reduction of Bcl-2, a significant reduction of MMP9, and an increase of *h*ENT1, which might at least in part explain the induction of apoptosis, the antimigratory, activity and the synergistic interaction with gemcitabine, as described previously.^49^

### 2.7 *In Vitro* ADME Assays

*In vitro* ADME properties were assessed for the best MAGL inhibitor of this series, compound **13**, in comparison with the previously published benzoylpiperidine derivative **5c**,^35^ and the results are reported in Table 2. Permeability was measured by the parallel artificial membrane permeability assay (PAMPA) to evaluate the ability of these compounds to reach MAGL in the cytoplasm. Interestingly, compound **13** displayed an increased membrane permeability (*P*_app_ of 3.695 × 10^–6^ cm/s) and a reduced membrane retention (38.9%) compared to **5c** (*P*_app_ of 2.067 × 10^–6^ cm/s and membrane retention of 52.6%). Moreover, compound **13** provided a slightly better value of metabolic stability in human liver microsomes (92.7%, expressed as percentage of unmodified compound) compared to **5c** (90.5%). Water solubility and stability tests performed in polar solvents (methanol and PBS) and in human plasma gave similar results for both compounds since both displayed solubility lower than 1 ng/mL and showed to be stable in polar solvents and human plasma for more than 24 h.

**Table 2:** *In Vitro* ADME Assays of Compounds **5c** and **13**

				stability		
compound	water solubility ng/mL (logS)	*P*_app_ × 10^–6^ cm/sec (RM %)	metabolic stability %	MeOH (h)	PBS pH 7.4 (h)	human plasma (h)
**5c**	<1	2.067	90.5	>24	>24	>24
	(< −8.702)	(52.6)				
**13**	<1	3.695	92.7	>24	>24	>24
	(< −8.676)	(38.9)				

## 3 Conclusions

In this work, we designed and synthesized a new class of MAGL inhibitors based on a benzylpiperidine scaffold. Among this series, we identified compound **13** as the most potent benzylpiperidine derivative with an IC_50_ value of 2.0 nM on isolated enzyme. Compound **13** is characterized by a reversible mechanism of action, a competitive behavior (*K*_i_ value of 1.42 nM), and a notable selectivity for MAGL compared to other targets of the endocannabinoid system, such as FAAH, CB1, CB2, ABHD6, and ABHD12.

The binding disposition of this class of compounds into the MAGL active site was suggested by molecular docking studies followed by molecular dynamics simulations and binding free energy evaluations.

After demonstrating that MAGL mRNA is overexpressed in pancreatic ductal adenocarcinoma tissues compared to normal pancreatic tissues and considering that MAGL mRNA overexpression was associated with poor patients’ prognosis, compound **13** was also subjected to preliminary pharmacological assays on pancreatic cancer cells. In these studies, **13** showed a moderate antiproliferative activity on SUIT-2 immortalized cancer cells and on PDAC2 and PDAC3 primary cell cultures (IC_50_ values ranging from 7.25 to 12.61 μM) compared to normal cells HPNE (IC_50_ > 20 μM). Moreover, compound **13** not only remarkably enhanced apoptosis induction in PDAC cells, but it also significantly reduced cell migration and exerted a synergistic effect when combined with the chemotherapeutic drug gemcitabine. Considering that the chosen PDAC preclinical model shares the same molecular complexity of the originator tumor, representing an important tool for the experimental testing of anti-cancer agents, all these results support the potential applicability to the clinical setting of this class of inhibitors.

Moreover, the completely new scaffold of compound **13**, deriving from the merging of the FAAH inhibitor PF-3845 **6** (Figure 2) and the benzoylpiperidine-based MAGL inhibitors (exemplified by compound **5a**, Figure 2), paves the way for a new chemical class of MAGL inhibitors.

Further research to confirm cellular MAGL engagement and structural optimization of this new class of MAGL inhibitors is ongoing.

## 4 Experimental Section

### 4.1 Synthesis: General Procedures and Materials

All solvents and chemicals were used as purchased without further purification. Chromatographic separations were performed on silica gel columns by flash chromatography (Kieselgel 40, 0.040–0.063 mm; Merck). Reactions were followed by thin layer chromatography (TLC) on Merck aluminum silica gel (60 F254) sheets that were visualized under a UV lamp. Evaporation was performed *in vacuo* (rotating evaporator). Sodium sulfate was always used as the drying agent. Proton (^1^H) and carbon (^13^C) NMR spectra were obtained with a Bruker Avance III 400 MHz spectrometer using the indicated deuterated solvents. Chemical shifts are given in parts per million (ppm) (δ relative to residual solvent peak for ^1^H and ^13^C). ^1^H-NMR spectra are reported in this order: multiplicity and number of protons. Standard abbreviation indicating the multiplicity was used as follows: s = singlet, d = doublet, dd = doublet of doublets, ddd = doublet of doublet of doublets, t = triplet, tt = triplet of triplets, dt = doublet of triplets, td = triplet of doublets, m = multiplet, bm = broad multiplet and bs = broad singlet. HPLC analysis was used to determine purity: all target compounds (i.e., assessed in biological assays) were ≥95% pure by HPLC, as confirmed via UV detection (λ = 254 nm). Analytical reversed-phase HPLC was conducted using a Kinetex EVO C18 column (5 μm, 150 × 4.6 mm, Phenomenex, Inc.); eluent A, water; eluent B, CH_3_CN; after 5 min. at 25% B, a gradient was formed from 25% to 75% of B in 5 min and held at 75% of B for 10 min; flow rate was 1 mL/min. HPLC analyses were performed at 254 nm. The ESI-MS spectra were recorded by direct injection at a 5 μL min^–1^ flow rate in an Orbitrap high-resolution mass spectrometer (Thermo, San Jose, CA, USA), equipped with a HESI source. The working conditions were as follows: positive polarity, spray voltage of 3.4 kV, capillary temperature of 290 °C, S-lens RF level 50. The sheath and the auxiliary gases were set at 24 and 5 (arbitrary units), respectively. For acquisition and analysis, Xcalibur 4.2 software (Thermo) was used. For spectra acquisition, a nominal resolution (at *m/z* 200) of 140,000 was used. Yields refer to isolated and purified products derived from non-optimized procedures. Compound 2-chloro-5-methoxybenzoic acid was synthesized as previously reported.^32^

#### 4.1.1 General Procedure for the Synthesis of Compounds **19**–**22**, **46**

Commercially available 3-bromophenol **18** or 4-bromophenol **43** (250 mg, 1 equiv) and 2-chloro-3-trifluoromethylpyridine **14**, 2-chloro-4-trifluoromethylpyridine **15**, 2-chloro-5-trifluoromethylpyridine **16**, or 2-chloro-6-trifluoromethylpyridine **17** (1 equiv) were mixed in anhydrous DMF (3.7 mL) and treated with anhydrous potassium carbonate (2 equiv). The mixture was stirred at 110 °C overnight, then cooled at room temperature, and partitioned between water and ethyl acetate. The organic layer was separated, dried over sodium sulfate, filtered, and concentrated. Silica gel column chromatography (1–5% EtOAc in *n*-hexane or petroleum ether) afforded the title compounds.

##### 4.1.1.1 2-(3-Bromophenoxy)-3-(trifluoromethyl)pyridine (**19**)

Light yellow oil. 82% yield from **14** and **18**. ^1^H NMR (CDCl_3_) δ (ppm): 7.08–7.17 (m, 2H), 7.29 (t, 1H, *J* = 8.1 Hz), 7.34–7.42 (m, 2H), 7.97–8.04 (m, 1H), 8.28–8.34 (m, 1H).

##### 4.1.1.2 2-(3-Bromophenoxy)-4-(trifluoromethyl)pyridine (**20**)

Light yellow oil. 99% yield from **15** and **18**. ^1^H-NMR (CDCl_3_) δ (ppm): 7.11 (ddd, 1H, *J* = 8.1, 2.2, 1.0 Hz), 7.17–7.20 (m, 1H), 7.21–7.25 (m, 1H), 7.30 (t, 1H, *J* = 8.1 Hz), 7.34 (t, 1H, *J* = 2.0 Hz), 7.39 (ddd, 1H, *J* = 8.0, 1.8, 1.0 Hz), 8.33 (d, 1H, *J* = 5.2 Hz).

##### 4.1.1.3 2-(3-Bromophenoxy)-5-(trifluoromethyl)pyridine (**21**)

Colorless oil. 77% yield from **16** and **18**. ^1^H-NMR (CDCl_3_) δ (ppm): 7.04 (d, 1H, *J* = 8.7 Hz), 7.11 (ddd, 1H, *J* = 8.2, 2.3, 1.0 Hz), 7.30 (t, 1H, *J =* 8.1 Hz), 7.34 (t, 1H, *J =* 2.1 Hz), 7.40 (ddd, 1H, *J* = 8.0, 1.8, 1.0 Hz), 7.93 (dd, 1H, *J* = 8.6, 2.2 Hz), 8.42–8.47 (m, 1H).

##### 4.1.1.4 2-(3-Bromophenoxy)-6-(trifluoromethyl)pyridine (**22**)

Light yellow oil. 82% yield from **17** and **18**. ^1^H-NMR (CDCl_3_) δ (ppm): 7.07 (d, 1H, *J* = 8.5 Hz), 7.11–7.17 (m, 1H), 7.26–7.31 (m, 1H), 7.34–7.39 (m, 2H), 7.41 (d, 1H, *J* = 7.4 Hz), 7.86 (t, 1H, *J* = 8.0 Hz).

##### 4.1.1.5 2-(4-Bromophenoxy)-5-(trifluoromethyl)pyridine (**46**)

Colorless liquid. 84% yield from **16** and **43**. ^1^H-NMR (CDCl_3_) δ (ppm): 7.01–7.08 (m, 3H), 7.54 (AA’XX’, 2H, *J_AX_* = 8.8 Hz, *J_AA’/XX’_* = 2.6 Hz), 7.92 (dd, 1H, *J* = 8.7, 2.5 Hz), 8.40–8.45 (m, 1H).

#### 4.1.2 General Procedure for the Synthesis of Compounds **44** and **45**

A vial was loaded with K_3_PO_4_ (2 equiv) and commercially available 3-bromophenol **18** (600 mg, 2 equiv). Then, in an inert atmosphere, copper(I) iodide (0.1 equiv) in anhydrous DMSO (1.4 mL) and commercially available 2-chloropyridine **41** or bromobenzene **42** (1 equiv) were added. The vial was sealed, and the reaction mixture was stirred at 130 °C. After the reaction mixture was heated for 24 h, it was cooled to room temperature and the workup consisted in the filtration of the reaction mixture through a Celite pad, washing it repeatedly with EtOAc. The filtrate was concentrated under vacuum to give a crude residue, which was then purified by flash column chromatography (silica gel, 2–5% EtOAc in *n*-hexane or petroleum ether), to give the desired compounds.

##### 4.1.2.1 2-(3-Bromophenoxy)pyridine (**44**)

Colorless oil. 32% yield from **41** and **18**. ^1^H NMR (CDCl_3_) δ (ppm): 6.94 (dt, 1H, *J* = 8.3, 0.8 Hz), 7.05 (ddd, 1H, *J* = 7.2, 5.0, 0.9 Hz), 7.09 (ddd, 1H, *J* = 8.1, 2.3, 1.1 Hz), 7.27 (t, 1H, *J* = 8.0 Hz), 7.29–7.36 (m, 2H), 7.73 (ddd, 1H, *J* = 8.8, 6.8, 2.0 Hz), 8.22 (ddd, 1H, *J* = 5.0, 2.0, 0.7 Hz).

##### 4.1.2.2 1-Bromo-3-phenoxybenzene (**45**)

Colorless oil. 11% yield from **42** and **18**. ^1^H NMR (CDCl_3_) δ (ppm): 6.94 (ddd, 1H, *J* = 7.7, 2.3, 1.5 Hz), 6.99–7.05 (m, 2H), 7.12–7.24 (m, 4H), 7.33–7.40 (m, 2H).

#### 4.1.3 General Procedure for the Synthesis of Compounds **23**–**26**, **47**–**49**

*tert*-Butyl-4-methylenepiperidine-1-carboxylate (1.25 equiv) in anhydrous toluene (2.2 mL) was treated with 9-BBN (0.5 M in THF, 1.25 equiv) and heated at 115 °C for 1 h. The reaction mixture was cooled and treated with NaOH (3.2 M aqueous solution, 3 equiv) followed by Pd(PPh_3_)_4_ (0.03 equiv). Finally, intermediates **19–22** and **44–46** (370 mg, 1 equiv) in anhydrous toluene (0.9 mL) and tetrabutylammonium iodide (0.5 equiv) were added. The reaction mixture was placed under argon and heated at 115 °C. After 18 h, the mixture was cooled and partitioned between EtOAc and saturated aqueous NaHCO_3_. The organic layer was separated and dried over sodium sulfate, filtered, and concentrated to obtain a crude, which was purified by silica gel column chromatography (5–20% EtOAc in *n*-hexane or petroleum ether) to give the desired products.

##### 4.1.3.1 *tert*-Butyl-4-(3-((3-(trifluoromethyl)pyridin-2-yl)oxy)benzyl)piperidine-1-carboxylate (**23**)

Yellow oil. 46% yield from **19**. ^1^H-NMR (CDCl_3_) δ (ppm): 1.44 (s, 9H), 1.49–1.91 (bm, 5H), 2.55 (d, 2H, *J* = 6.8 Hz), 2.64 (td, 2H, *J* = 13.0, 2.5 Hz), 4.02–4.11 (m, 2H), 6.95 (t, 1H, *J* = 1.8 Hz), 6.98–7-04 (m, 2H), 7.08 (ddd, 1H, *J* = 7.5, 5.0, 0.7 Hz), 7.33 (t, 1H, *J* = 7.9 Hz), 7.96–8.01 (m, 1H), 8.26–8.31 (m, 1H).

##### 4.1.3.2 *tert*-Butyl-4-(3-((4-(trifluoromethyl)pyridin-2-yl)oxy)benzyl)piperidine-1-carboxylate (**24**)

Orange oil. 99% yield from **20**. ^1^H-NMR (CDCl_3_) δ (ppm): 1.45 (s, 9H), 1.59–1.74 (m, 3H), 1.75–1.92 (m, 1H), 2.56 (d, 2H, *J* = 6.8 Hz), 2.64 (td, 2H, *J* = 12.9, 2.2 Hz), 4.02–4.12 (m, 2H), 4.31–4.43 (bm, 1H), 6.92 (t, 1H, *J* = 1.9 Hz), 6.99 (ddd, 1H, *J* = 8.1, 2.3, 0.9 Hz), 7.01–7.05 (m, 1H), 7.10–7.14 (m, 1H), 7.17–7.22 (m, 1H), 7.34 (t, 1H, *J* = 7.8 Hz), 8.33 (d, 1H, *J* = 5.2 Hz).

##### 4.1.3.3 *tert*-Butyl-4-(3-((5-(trifluoromethyl)pyridin-2-yl)oxy)benzyl)piperidine-1-carboxylate (**25**)

Colorless oil. 99% yield from **21**. ^1^H-NMR (CDCl_3_) δ (ppm): 1.05–1.23 (m, 3H), 1.45 (s, 9H), 1.60–1.74 (m, 3H), 2.56 (d, 2H, *J =* 6.8 Hz), 2.59–2.70 (bm, 2H), 4.00–4.15 (bm, 1H), 6.91–6.94 (m, 1H), 6.97–7.01 (m, 2H), 7.01–7.06 (m, 1H), 7.34 (t, 1H, *J =* 7.9 Hz), 7.90 (dd, 1H, *J =* 8.6, 2.1 Hz), 8.42–8.47 (m, 1H).

##### 4.1.3.4 *tert*-Butyl-4-(3-((6-(trifluoromethyl)pyridin-2-yl)oxy)benzyl)piperidine-1-carboxylate (**26**)

Colorless oil. 97% yield from **22**. ^1^H-NMR (CDCl_3_) δ (ppm): 1.45 (s, 9H), 1.50–1.73 (bm, 3H), 1.78–1.91 (bm, 2H), 2.54 (d, 2H, *J* = 6.8 Hz), 2.64 (td, 2H, *J* = 12.6, 2.6 Hz), 4.03–4.12 (m, 2H), 6.95–7.06 (m, 4H), 7.31 (t, 1H, *J* = 7.8 Hz), 7.37 (d, 1H, *J* = 7.4 Hz), 7.82 (t, 1H, *J* = 7.9 Hz).

##### 4.1.3.5 *tert*-Butyl-4-(3-(pyridin-2-yloxy)benzyl)piperidine-1-carboxylate (**47**)

Colorless oil. 30% yield from **44**. ^1^H-NMR (CDCl_3_) δ (ppm): 1.46 (s, 9H), 1.48–1.77 (m, 5H), 2.54 (d, 2H, *J* = 6.9 Hz), 2.58–2.70 (m, 2H), 3.95–4.18 (bm, 2H), 6.85–6.93 (m, 3H), 6.95–7.02 (m, 2H), 7.30 (t, 1H, *J* = 7.8 Hz), 7.65–7.72 (m, 1H), 8.21 (ddd, 1H, *J* = 5.0, 2.1, 0.8 Hz).

##### 4.1.3.6 *tert*-Butyl 4-(3-phenoxybenzyl)piperidine-1-carboxylate (**48**)

Light yellow oil. 78% yield from **45**. ^1^H-NMR (CDCl_3_) δ (ppm): 1.45 (s, 9H), 1.51–1.71 (m, 4H), 1.83–1.91 (m, 1H), 2.48–2.56 (m, 2H), 2.64 (td, 2H, *J =* 12.9, 2.2), 4.01–4.12 (m, 2H), 6.79–6.85 (m, 2H), 6.86–6.90 (m, 1H), 6.97–7.03 (m, 2H), 7.07–7.15 (m, 1H), 7.23 (t, 1H, *J* = 7.8 Hz), 7.27–7.36 (m, 2H).

##### 4.1.3.7 *tert*-Butyl-4-(4-((5-(trifluoromethyl)pyridin-2-yl)oxy)benzyl)piperidine-1-carboxylate (**49**)

Colorless oil. 70% yield from **46**. ^1^H-NMR (CDCl_3_) δ (ppm): 1.10–1.22 (bm, 1H), 1.45 (s, 9H), 1.59–1.71 (bm, 3H), 1.80–1.92 (bm, 1H), 2.56 (d, 2H, *J* = 6.9 Hz), 2.60–2.70 (m, 2H), 4.04–4.13 (m, 2H), 6.99 (d, 1H, *J* = 6.3 Hz), 7.06 (d, 2H, *J* = 8.4 Hz), 7.19 (d, 2H, *J* = 8.4 Hz), 7.89 (dd, 1H, *J* = 8.7, 2.4 Hz), 8.41–8.47 (m, 1H).

#### 4.1.4 General Procedure for the Synthesis of Compounds **27**–**30**, **50**–**52**

*N*-Boc-piperidine intermediates **23–26** and **47–49** (490 mg, 1 equiv) were dissolved in methanol (1.7 mL) and dichloromethane (1.7 mL), treated dropwise with HCl (4.0 M in dioxane, 6 equiv), and stirred at room temperature for 1 h. Toluene (1.8 mL) was added, and the reaction mixture was concentrated under nitrogen flux. A second evaporation from toluene (1.8 mL) followed by high vacuum afforded the title compounds, which were used in the next step without further purification.

##### 4.1.4.1 2-(3-(Piperidin-4-ylmethyl)phenoxy)-3-(trifluoromethyl)pyridine Hydrochloride (**27**)

White solid. 99% yield from **23**. ^1^H-NMR (D_2_O) δ (ppm): 1.32–2.02 (m, 5H), 2.64 (d, 2H, *J* = 5.6 Hz), 2.83–3.01 (m, 2H), 3.31–3.48 (m, 2H), 6.96–7.12 (m, 2H), 7.12–7.24 (m, 1H), 7.24–7.36 (m, 1H), 7.37–7.50 (m, 1H), 8.14–8.28 (m, 2H).

##### 4.1.4.2 2-(3-(Piperidin-4-ylmethyl)phenoxy)-4-(trifluoromethyl)pyridine Hydrochloride (**28**)

White solid. 99% yield from **24**. ^1^H-NMR (D_2_O) δ (ppm): 1.30–2.03 (m, 4H), 2.66 (d, 2H, *J* = 6.8 Hz), 2.87–3.02 (m, 2H), 3.37–3.46 (m, 2H), 4.29–4.47 (bm, 1H), 7.03–7.11 (m, 2H), 7.20 (d, 1H, *J* = 7.5 Hz), 7.30–7.35 (m, 1H), 7.41–7.50 (m, 2H), 8.30 (d, 1H, *J* = 5.2 Hz).

##### 4.1.4.3 2-(3-(Piperidin-4-ylmethyl)phenoxy)-5-(trifluoromethyl)pyridine Hydrochloride (**29**)

Light yellow solid. 99% yield from **25**. ^1^H-NMR (D_2_O) δ (ppm): 1.31–1.95 (m, 4H), 2.63 (d, 2H, *J =* 6.7 Hz), 2.85–2.98 (m, 2H), 3.34–3.44 (m, 2H), 4.33–4.42 (bm, 1H), 7.01–7.08 (m, 2H), 7.12 (d, 1H, *J =* 8.8 Hz), 7.18 (d, 1H, *J =* 7.8 Hz), 7.42 (t, 1H, *J =* 7.8 Hz), 8.12 (dd, 1H, *J =* 8.5, 2.3 Hz), 8.37–8.43 (m, 1H).

##### 4.1.4.4 2-(3-(Piperidin-4-ylmethyl)phenoxy)-6-(trifluoromethyl)pyridine Hydrochloride (**30**)

White solid. 99% yield from **26**. ^1^H-NMR (D_2_O) δ (ppm): 1.30–1.95 (bm, 5H), 2.62 (d, 2H, *J* = 6.4 Hz), 2.83–2.98 (bm, 2H), 3.32–3.45 (bm, 2H), 7.00–7.09 (m, 2H), 7.11–7.20 (m, 2H), 7.41 (t, 1H, *J* = 8.2 Hz), 7.59 (d, 1H, *J* = 7.4 Hz), 8.01 (t, 1H, *J* = 7.6 Hz).

##### 4.1.4.5 2-(3-(Piperidin-4-ylmethyl)phenoxy)pyridine Hydrochloride (**50**)

Yellow solid. 90% yield from **47**. ^1^H-NMR (D_2_O) δ (ppm): 1.35–1.53 (m, 3H), 1.79–2.00 (m, 2H), 2.68 (d, 2H, *J* = 7.0 Hz), 2.93 (td, 2H, *J* = 12.9, 2.5 Hz), 3.35–3.45 (m, 2H), 7.14–7.20 (m, 3H), 7.28–7.32 (m, 1H), 7.47–7.55 (m, 2H), 8.31 (ddd, 1H, *J* = 8.8, 7.0, 1.9 Hz), 8.34–8.38 (m, 1H).

##### 4.1.4.6 4-(3-Phenoxybenzyl)Piperidine Hydrochloride (**51**)

Yellow oil. 99% yield from **48**. ^1^H-NMR (D_2_O) δ (ppm): 1.80–1.97 (m, 4H), 2.25–2.40 (bm, 1H), 2.56–2.68 (m, 2H), 2.87–3.01 (m, 2H), 3.34–3.48 (m, 2H), 6.89–7.00 (m, 2H), 7.03–7.13 (m, 2H), 7.15–7.33 (m, 3H), 7.34–7.50 (m, 2H).

##### 4.1.4.7 2-(4-(Piperidin-4-Ylmethyl)Phenoxy)-5-(Trifluoromethyl)Pyridine Hydrochloride (**52**)

White solid. 91% yield from **49**. ^1^H-NMR (D_2_O) δ (ppm): 1.36–1.52 (m, 2H), 1.84–1.98 (m, 3H), 2.65 (d, 2H, *J* = 6.7 Hz), 2.87–3.00 (m, 2H), 3.35–3.46 (m, 2H), 7.08–7.16 (m, 3H), 7.32 (d, 2H, *J* = 8.5 Hz), 8.12 (dd, 1H, *J* = 8.8, 2.6 Hz), 8.37–8.42 (m, 1H).

#### 4.1.5 General Procedure for the Synthesis of Compounds **31**–**40**, **53**–**55**

HATU (1.05 equiv) was added to a solution of the appropriate commercially available or in-house synthesized benzoic acid (3-methoxybenzoic acid for **31**, **37–39**, **53–55**, 2-fluoro-5-methoxybenzoic acid for **32** and **40**, 4-fluoro-3-methoxybenzoic acid for **33**, 2-chloro-5-methoxybenzoic acid for **34**, 4-chloro-3-methoxybenzoic acid for **35**, and 4-bromo-5-methoxybenzoic acid for **36**; 1 equiv) in dry DMF (5.7 mL), and then DIPEA (4 equiv) was added dropwise. The resulting mixture was stirred at room temperature for 30 min, and then piperidine hydrochlorides **27–30** and **50–52** (460 mg, 1 equiv) were added and left under stirring at room temperature until consumption of starting material (TLC). After this time, the residue was diluted with water and extracted with EtOAc. The organic layer was repeatedly washed with brine and dried over Na_2_SO_4_, and the solvent was removed under reduced pressure. The residue was purified with a flash column chromatography (silica gel, mixtures from 8:2 to 6:4 of *n*-hexane or petroleum ether/ethyl acetate), and pure fractions containing the desired compounds were evaporated to dryness affording the amides.

##### 4.1.5.1 (3-Methoxyphenyl)(4-(3-((5-(trifluoromethyl)pyridin-2-yl)oxy)benzyl)piperidin-1-yl)methanone (**31**)

Yellow oil. 66% yield from **29** and 3-methoxybenzoic acid. ^1^H-NMR (CDCl_3_) δ (ppm): 1.50–1.69 (m, 3H), 1.74–1.88 (bm, 2H), 2.53–2.64 (m, 2H), 2.65–2.80 (bm, 1H), 2.84–3.02 (bm, 1H), 3.70–3.79 (bm, 1H), 3.81 (s, 3H), 4.64–4.77 (bm, 1H), 6.90–6.95 (m, 4H), 6.97–7.02 (m, 2H), 7.02–7.06 (m, 1H), 7.24–7.32 (m, 1H), 7.35 (t, 1H, *J =* 7.8 Hz), 7.89 (dd, 1H, *J =* 8.7, 2.5 Hz), 8.41–8.45 (m, 1H).

##### 4.1.5.2 (2-Fluoro-5-methoxyphenyl)(4-(3-((5-(trifluoromethyl)pyridin-2-yl)oxy)benzyl)piperidin-1-yl)methanone (**32**)

Yellow oil. 41% yield from **29** and 2-fluoro-5-methoxybenzoic acid. ^1^H-NMR (CDCl_3_) δ (ppm): 1.58–1.68 (bm, 3H), 1.75–1.86 (bm, 2H), 2.52–2.65 (m, 2H), 2.67–2.78 (m, 1H), 2.80–3.12 (bm, 1H), 3.53–3.64 (m, 1H), 3.78 (s, 3H), 4.70–4.79 (m, 1H), 6.80–7.08 (m, 7H), 7.34 (t, 1H, *J =* 7.8 Hz), 7.90 (dd, 1H, *J =* 8.6, 2.6 Hz), 8.41–8.46 (m, 1H).

##### 4.1.5.3 (4-Fluoro-3-methoxyphenyl)(4-(3-((5-(trifluoromethyl)pyridin-2-yl)oxy)benzyl)piperidin-1-yl)methanone (**33**)

Yellow solid. 53% yield from **29** and 4-fluoro-3-methoxybenzoic acid. ^1^H-NMR (CDCl_3_) δ (ppm): 1.10–1.40 (m, 2H), 1.60–1.90 (m, 3H), 2.60 (d, 2H, *J =* 7.2 Hz), 2.61–3.06 (bm, 2H), 3.70–3.84 (bm, 1H), 3.90 (s, 3H), 4.60–4.75 (bm, 1H), 6.90 (ddd, 1H, *J* = 8.2, 4.3, 2.0 Hz), 6.92–6.95 (m, 1H), 6.98–7.10 (m, 5H), 7.35 (t, 1H, *J =* 7.8 Hz), 7.90 (dd, 1H, *J =* 8.7, 2.5 Hz), 8.40–8.47 (m, 1H).

##### 4.1.5.4 (2-Chloro-5-methoxyphenyl)(4-(3-((5-(trifluoromethyl)pyridin-2-yl)oxy)benzyl)piperidin-1-yl)methanone (**34**)

Light yellow oil. 75% yield from **29** and 2-chloro-5-methoxybenzoic acid. ^1^H-NMR (CDCl_3_; asterisk denotes isomer peaks) δ (ppm): 1.20–1.48 (bm, 3H), 1.72–1.88 (bm, 2H), 2.51–2.64 (m, 2H), 2.65–2.79 (m, 1H), 2.82–2.94 (m, 1H), 2.98–3.10* (m, 1H), 3.37–3.48 (m,1H), 3.77* (s, H), 3.80 (s, 3H), 4.68–4.82 (m, 1H), 6.74 (d, 1H, *J* = 2.9 Hz), 6.80–6.87 (m, 2H), 6.90–6.95 (m, 1H), 6.96–7.07 (m, 2H), 7.23–7.30 (m, 1H), 7.34 (t, 1H, *J =* 7.9 Hz), 7.89 (dd, 1H, *J =* 8.9, 2.5 Hz), 8.40–8.46 (m, 1H).

##### 4.1.5.5 (4-Chloro-3-methoxyphenyl)(4-(3-((5-(trifluoromethyl)pyridin-2-yl)oxy)benzyl)piperidin-1-yl)methanone (**35**)

Yellow oil. 48% yield from **29** and 4-chloro-3-methoxybenzoic acid. ^1^H-NMR (CDCl_3_) δ (ppm): 1.08–1.33 (bm, 2H), 1.60–1.90 (bm, 3H), 2.61 (d, 2H, *J =* 7.4 Hz), 2.66–2.83 (bm, 1H), 2.84–3.05 (bm, 1H), 3.67–3.83 (bm, 1H), 3.91 (s, 3H), 4.59–4.77 (bm, 1H), 6.88 (dd, 1H *J =* 8.0, 1.8 Hz), 6.93 (t, 1H, *J =* 1.9 Hz), 6.97–7.07 (m, 4H), 7.32–7.39 (m, 2H), 7.90 (dd, 1H, *J =* 8.4, 2.6 Hz), 8.41–8.46 (m, 1H).

##### 4.1.5.6 (4-Bromo-3-methoxyphenyl)(4-(3-((5-(trifluoromethyl)pyridin-2-yl)oxy)benzyl)piperidin-1-yl)methanone (**36**)

Yellow solid. 54% yield from **29** and 4-bromo-5-methoxybenzoic acid. ^1^H-NMR (CDCl_3_) δ (ppm): 1.10–1.40 (bm, 2H), 1.60–1.80 (bm, 3H), 2.60 (d, 2H, *J =* 6.5 Hz), 2.65–2.82 (bm, 1H), 2.85–3.04 (bm, 1H), 3.65–3.80 (bm, 1H), 3.91 (s, 3H), 4.60–4.73 (bm, 1H), 6.81 (dd, 1H, *J =* 8.0, 1.8 Hz), 6.90–6.96 (m, 2H), 6.98–7.07 (m, 3H), 7.35 (t, 1H, *J =* 7.9 Hz), 7.54 (d, 1H, *J =* 8.0 Hz), 7.90 (dd, 1H, *J =* 8.5, 2.4 Hz), 8.41–8.46 (m, 1H).

##### 4.1.5.7 (3-Methoxyphenyl)(4-(3-((3-(trifluoromethyl)pyridin-2-yl)oxy)benzyl)piperidin-1-yl)methanone (**37**)

Light yellow oil. 48% yield from **27** and 3-methoxybenzoic acid. ^1^H-NMR (CDCl_3_) δ (ppm): 1.08–1.37 (bm, 2H), 1.66–1.91 (bm, 3H), 2.59 (d, 2H, *J* = 5.8 Hz), 2.65–3.03 (bm, 2H), 3.62–3.83 (bm, 1H), 3.81 (s, 3H), 4.59–4.78 (bm, 1H), 6.89–6.97 (m, 4H), 6.98–7.05 (m, 2H), 7.08 (dd, 1H, *J* = 7.6, 5.2 Hz), 7.26–7.31 (m, 1H), 7.33 (t, 1H, *J* = 7.9 Hz), 7.96–8.01 (m, 1H), 8.25–8.31 (m, 1H).

##### 4.1.5.8 (3-Methoxyphenyl)(4-(3-((4-(trifluoromethyl)pyridin-2-yl)oxy)benzyl)piperidin-1-yl)methanone (**38**)

Yellow oil. 44% yield from **28** and 3-methoxybenzoic acid. ^1^H-NMR (CDCl_3_) δ (ppm): 1.11–1.35 (bm, 1H), 1.65–1.92 (bm, 4H), 2.60 (d, 2H, *J* = 6.6 Hz), 2.68–3.06 (bm, 2H), 3.62–3.98 (bm, 1H), 3.82 (s, 3H), 4.50–4.88 (bm, 1H), 6.90–6.97 (m, 4H), 7.00 (dd, 1H, *J* = 8.2, 2.0 Hz), 7.02–7.06 (m, 1H), 7.13 (s, 1H), 7.20 (d, 1H, *J* = 5.4 Hz), 7.26–7.32 (m, 1H), 7.34 (t, 1H, *J* = 7.8 Hz), 8.32 (d, 1H, *J* = 5.2 Hz).

##### 4.1.5.9 (3-Methoxyphenyl)(4-(3-((6-(trifluoromethyl)pyridin-2-yl)oxy)benzyl)piperidin-1-yl)methanone (**39**)

Yellow oil. 61% yield from **30** and 3-methoxybenzoic acid. ^1^H-NMR (CDCl_3_) δ (ppm): 1.50–1.88 (bm, 6H), 2.50–3.00 (bm, 4H), 3.62–3.84 (bm, 1H), 3.82 (s, 3H), 6.89–7.08 (m, 7H), 7.27–7.41 (m, 3H), 7.83 (t, 1H, *J* = 7.8 Hz).

##### 4.1.5.10 (2-Fluoro-5-methoxyphenyl)(4-(3-((4-(trifluoromethyl)pyridin-2-yl)oxy)benzyl)piperidin-1-yl)methanone (**40**)

Yellow oil, 72% yield from **28** and 2-fluoro-5-methoxybenzoic acid. ^1^H-NMR (CDCl_3_) δ (ppm): 1.21–1.36 (bm, 1H), 1.56–1.68 (bm, 2H), 1.74–1.86 (bm, 2H), 2.52–2.66 (m, 2H), 2.66–2.78 (m, 1H), 2.82–3.12 (bm, 1H), 3.51–3.61 (m, 1H), 3.78 (s, 3H), 4.68–4.89 (m, 1H), 6.81–6.90 (m, 2H), 6.91–6.94 (m, 1H), 6.95–7.01 (m, 2H), 7.01–7.05 (m, 1H), 7.11–7.15 (m, 1H), 7.18–7.21 (m, 1H), 7.34 (t, 1H, *J* = 7.9 Hz), 8.32 (d, 1H, *J* = 5.2 Hz). ^13^C NMR (CDCl_3_) δ (ppm): 31.84, 32.63, 38.28, 42.31, 42.87, 47.45, 55.98, 108.09 (q, *J* = 4.1 Hz), 113.14 (d, *J* = 3.8 Hz), 114.12 (q, *J* = 3.2 Hz), 116.58 (d, *J* = 23.6 Hz), 116.81 (d, *J* = 8.0 Hz), 119.09, 122.01, 122.61 (q, *J* = 273.1 Hz), 124.97 (d, *J* = 19.9 Hz), 126.26, 129.79, 141.85 (q, *J* = 34.2 Hz), 142.22, 149.16, 152.47 (d, *J* = 240.0 Hz), 153.48, 156.07 (d, *J* = 2.0 Hz), 164.34. 164.98. HPLC analysis: retention time = 14.186 min; peak area, 95% (254 nm). HRMS: *m/z* for C_26_H_25_F_4_N_2_O_3_ [M + H]^+^ calculated: 489.17958, found: 489.17963.

##### 4.1.5.11 (3-Methoxyphenyl)(4-(3-(pyridin-2-yloxy)benzyl)piperidin-1-yl)methanone (**53**)

Yellow oil. 53% yield from **50** and 3-methoxybenzoic acid. ^1^H-NMR (CDCl_3_) δ (ppm): 1.55–1.87 (bm, 5H), 2.50–3.00 (bm, 4H), 3.65–3.80 (bm, 1H), 3.81 (s, 3H), 4.61–4.78 (bm, 1H), 6.85–7.04 (m, 8H), 7.26–7.35 (m, 2H), 7.70 (ddd, 1H, *J* = 8.8, 6.7, 1.6 Hz), 8.21 (ddd, 1H, *J* = 5.0, 2.0, 0.7 Hz).

##### 4.1.5.12 (3-Methoxyphenyl)(4-(3-phenoxybenzyl)piperidin-1-yl)methanone (**54**)

Light yellow oil. 67% yield from **51** and 3-methoxybenzoic acid. ^1^H-NMR (CDCl_3_) δ (ppm): 1.10–1.41 (bm, 1H), 1.50–1.87 (bm, 4H), 2.54 (d, 2H, *J* = 7.2 Hz), 2.61–3.00 (bm, 2H), 3.66–3.87 (bm, 1H), 3.81 (s, 3H), 4.51–4.81 (bm, 1H), 6.80–6.90 (m, 3H), 6.90–6.95 (m, 3H), 6.97–7.02 (m, 2H), 7.07–7.15 (m, 1H), 7.21 (t, 1H, *J* = 7.4 Hz), 7.25–7.36 (m, 3H).

##### 4.1.5.13 (3-Methoxyphenyl)(4-(4-((5-(trifluoromethyl)pyridin-2-yl)oxy)benzyl)piperidin-1-yl)methanone (**55**)

Light yellow oil. 72% yield from **52** and 3-methoxybenzoic acid. ^1^H-NMR (CDCl_3_) δ (ppm): 1.14–1.37 (bm, 3H), 1.72–1.89 (bm, 2H), 2.59 (d, 2H, *J* = 7.2 Hz), 2.66–3.00 (bm, 2H), 3.69–3.87 (bm, 1H), 3.82 (s, 3H), 4.59–4.82 (bm, 1H), 6.90–6.97 (m, 3H), 7.00 (d, 1H, *J* = 8.8 Hz), 7.07 (d, 2H, *J* = 8.5 Hz), 7.19 (d, 2H, *J* = 8.5 Hz), 7.30 (dd, 1H, *J* = 9.1, 7.4 Hz), 7.89 (dd, 1H, *J* = 8.6, 2.3 Hz), 8.41–8.46 (m, 1H).

#### 4.1.6 General Procedure for the Synthesis of Compounds **7**–**9**, **10a**–**e**, **11a**–**c**, **12**, **13**

A solution of *O*-methylated amides **31–40** and **53–55** (345 mg, 0.738 mmol) in anhydrous CH_2_Cl_2_ (8.6 mL) was cooled to −10 °C and treated dropwise with a 1.0 M solution of BBr_3_ in CH_2_Cl_2_ (2.3 mL) under argon. The mixture was left under stirring at the same temperature for 5 min and then at 0 °C for 1 h and finally at room temperature until the starting material was consumed (TLC). The mixture was then diluted with water and extracted with ethyl acetate. The organic phase was washed with brine, dried, and concentrated. The crude product was purified by flash chromatography over silica gel. Elution with *n*-hexane/EtOAc (4:6 to 6:4) or CHCl_3_/MeOH (95:5 to 99:1) mixtures afforded the desired compounds.

##### 4.1.6.1 (3-Hydroxyphenyl)(4-(3-((5-(trifluoromethyl)pyridin-2-yl)oxy)benzyl)piperidin-1-yl)methanone (**7**)

White solid, 59% yield from **31**. ^1^H-NMR (DMSO-*d*_6_) δ (ppm): 1.00–1.20 (bm, 2H), 1.46–1.70 (bm, 2H), 1.73–1.87 (bm, 1H), 2.56 (d, 2H, *J =* 6.8 Hz), 2.60–2.80 (bm, 1H), 2.86–3.00 (bm, 1H), 3.50–3.66 (bm, 1H), 4.30–4.50 (bm, 1H), 6.68–6.75 (m, 2H), 6.80 (ddd, 1H, *J* = 8.2, 2.5, 0.9 Hz), 6.99–7.05 (m, 2H), 7.09 (d, 1H, *J =* 7.8 Hz), 7.17–7.23 (m, 2H), 7.35 (dd, 1H, *J* = 8.6, 7.7 Hz), 8.22 (dd, 1H, *J =* 8.9, 2.4 Hz), 8.54–8.60 (m, 1H), 9.65 (exchangeable s, 1H). ^13^C NMR (DMSO-*d*_6_) δ (ppm): 31.27, 31.99, 37.29, 41.44, 41.67, 47.06, 111.65, 113.35, 116.14, 116.96, 118.97, 120.28 (q, *J* = 32.6 Hz), 121.94, 123.90 (q, *J* = 271.4 Hz), 126.03, 129.48, 129.53, 137.56 (q, *J* = 3.1 Hz), 137.71, 142.28, 145.34 (q, *J* = 4.4 Hz), 152.84, 157.22, 165.60, 168.74. HPLC analysis: retention time = 12.879 min; peak area, 98% (254 nm). HRMS: *m/z* for C_25_H_24_F_3_N_2_O_3_ [M + H]^+^ calculated: 457.17335, found: 457.17307.

##### 4.1.6.2 (3-Hydroxyphenyl)(4-(3-(pyridin-2-yloxy)benzyl)piperidin-1-yl)methanone (**8**)

White solid, 50% yield from **53**. ^1^H-NMR (DMSO-*d*_6_) δ (ppm): 1.00–1.20 (bm, 2H), 1.44–1.87 (bm, 3H), 2.54 (d, 2H, *J* = 7.1 Hz), 2.60–2.80 (bm, 1H), 2.81–3.02 (bm, 1H), 3.45–3.66 (bm, 1H), 4.30–4.48 (bm, 1H), 6.67–6.75 (m, 2H), 6.80 (ddd, 1H, *J* = 8.1, 2.5, 0.9 Hz), 6.90–6.95 (m, 2H), 6.96–7.05 (m, 2H), 7.12 (ddd, 1H, *J* = 7.2, 5.0, 0.9 Hz), 7.20 (t, 1H, *J* = 7.9 Hz), 7.31 (tt, 1H, *J* = 8.8, 7.6 Hz), 7.84 (ddd, 1H, *J* = 8.8, 6.8, 1.6 Hz), 8.15 (ddd, 1H, *J* = 5.0, 2.0, 0.8 Hz), 9.65 (exchangeable bs, 1H). ^13^C NMR (DMSO-*d*_6_) δ (ppm): 31.24, 31.96, 37.29, 41.47, 41.70, 47.02, 111.44, 113.32, 116.11, 116.94, 118.47, 118.94, 121.50, 125.10, 129.29, 129.45, 137.68, 140.07, 141.97, 147.44, 153.82, 157.19, 163.01, 168.71.

HPLC analysis: retention time = 11.703 min; peak area, 95% (254 nm). HRMS: *m/z* for C_24_H_25_N_2_O_3_ [M + H]^+^ calculated: 389.18597, found: 389.18588.

##### 4.1.6.3 (3-Hydroxyphenyl)(4-(3-phenoxybenzyl)piperidin-1-yl)methanone (**9**)

White solid, 62% yield from **54**. ^1^H-NMR (DMSO-*d*_6_) δ (ppm): 1.00–1.20 (bm, 2H), 1.43–1.84 (bm, 3H), 2.50–2.57 (m, 2H), 2.60–2.80 (bm, 1H), 2.80–3.00 (bm, 1H), 3.46–3.64 (bm, 1H), 4.30–4.50 (bm, 1H), 6.67–7.76 (m, 2H), 6.78–6.86 (m, 3H), 6.94–7.01 (m, 2H), 7.12 (tt, 1H, *J* = 7.4, 1.1 Hz), 7.15–7.23 (m, 2H), 7.24–7.31 (m, 1H), 7.35–7.41 (m, 2H), 9.64 (exchangeable s, 1H). ^13^C NMR (DMSO-*d*_6_) δ (ppm): 31.20, 31.43, 37.33, 41.50, 41.81, 47.13, 113.39, 116.20, 117.02, 118.49 (2C), 119.33, 123.32, 124.26, 128.19, 129.53, 129.73, 130.02 (2C), 137.74, 142.41, 156.52, 156.79, 157.26, 168.82. HPLC analysis: retention time = 12.997 min; peak area, 95% (254 nm). HRMS: *m/z* for C_25_H_26_NO_3_ [M + H]^+^ calculated: 388.19072, found: 388.19031.

##### 4.1.6.4 (2-Fluoro-5-hydroxyphenyl)(4-(3-((5-(trifluoromethyl)pyridin-2-yl)oxy)benzyl)piperidin-1-yl)methanone (**10a**)

White solid, 52% yield from **32**. ^1^H-NMR (DMSO-*d*_6_) δ (ppm): 1.02–1.16 (bm, 2H), 1.49–1.60 (bm, 1H), 1.62–1.73 (bm, 1H), 1.73–1.88 (bm, 1H), 2.55 (d, 2H, *J =* 6.9 Hz), 2.65–2.77 (m, 1H), 2.90–3.04 (bm, 1H), 3.35–3.49 (m, 1H), 4.40–4.49 (m, 1H), 6.57–6.67 (bm, 1H), 6.75–6.83 (m, 1H), 6.99–7.12 (m, 4H), 7.20 (d, 1H, *J =* 8.7 Hz), 7.35 (dd, 1H, *J* = 8.7, 7.6 Hz), 8.22 (dd, 1H, *J =* 9.0, 2.4 Hz), 8.54–8.58 (m, 1H), 9.64 (exchangeable bs, 1H). ^13^C NMR (DMSO-*d*_6_) δ (ppm): 31.23, 32.01, 37.17, 41.17, 41.59, 46.55, 111.67, 113.91, 116.34 (d, *J* = 23.2 Hz), 117.07 (d, *J* = 7.5 Hz), 118.99, 120.29 (q, *J* = 32.4 Hz), 121.97, 123.91 (q, *J* = 271.4 Hz), 124.90 (d, *J* = 20.4 Hz), 126.05, 129.54, 137.57 (q, *J* = 3.3 Hz), 142.24, 145.34 (q, *J* = 4.3 Hz) 150.67 (d, *J* = 235.1 Hz), 152.86, 153.71 (d, *J* = 1.9 Hz), 163.64, 165.62. HPLC analysis: retention time = 13.049 min; peak area, 95% (254 nm). HRMS: *m/z* for C_25_H_23_F_4_N_2_O_3_ [M + H]^+^ calculated: 475.16393, found: 475.16394.

##### 4.1.6.5 (4-Fluoro-3-hydroxyphenyl)(4-(3-((5-(trifluoromethyl)pyridin-2-yl)oxy)benzyl)piperidin-1-yl)methanone (**10b**)

White solid, 56% yield from **33**. ^1^H-NMR (DMSO-*d*_6_) δ (ppm): 1.07–1.19 (m, 2H), 1.49–1.72 (bm, 2H), 1.74–1.86 (bm, 1H), 2.56 (d, 2H, *J =* 6.9 Hz), 2.62–2.82 (bm, 1H), 2.82–3.01 (bm, 1H), 3.50–3.68 (bm, 1H), 4.30–4.45 (bm, 1H), 6.76 (ddd, 1H, *J* = 8.3, 4.3, 2.1 Hz), 6.91 (dd, 1H, *J =* 8.5, 2.0 Hz), 7.00–7.04 (m, 2H), 7.09 (d, 1H, *J =* 7.7 Hz), 7.13–7.22 (m, 2H), 7.36 (dd, 1H, *J* = 8.7, 7.6 Hz), 8.22 (dd, 1H, *J =* 8.8, 2.6 Hz), 8.54–8.58 (m, 1H), 10.16 (exchangeable bs, 1H). ^13^C NMR (DMSO-*d*_6_) δ (ppm): 31.35, 37.26, 41.65, 47.11, 111.65, 116.00 (d, *J* = 18.9 Hz), 116.35 (d, *J* = 3.4 Hz), 117.86 (d, *J* = 6.9 Hz), 118.97, 120.27 (q, *J* = 32.5 Hz), 121.93, 123.89 (q, *J* = 271.4 Hz), 126.01, 129.52, 132.91 (d, *J* = 3.7 Hz), 137.56 (q, *J* = 3.1 Hz), 142.26, 144.80 (d, *J* = 12.5 Hz), 145.34 (q, *J* = 4.4 Hz), 151.36 (d, *J* = 243.6 Hz), 152.84, 165.59, 167.99. HPLC analysis: retention time = 13.029 min; peak area, 98% (254 nm). HRMS: *m/z* for C_25_H_23_F_4_N_2_O_3_ [M + H]^+^ calculated: 475.16393, found: 475.16397.

##### 4.1.6.6 (2-Chloro-5-hydroxyphenyl)(4-(3-((5-(trifluoromethyl)pyridin-2-yl)oxy)benzyl)piperidin-1-yl)methanone (**10c**)

White solid, 57% yield from **34**. ^1^H-NMR (DMSO-*d*_6_, asterisk denotes isomer peaks) δ (ppm): 1.00–1.20 (bm, 1H), 1.47–1.58 (bm, 1H), 1.63–1.72 (bm, 1H), 1.73–1.87 (bm, 1H), 2.54 (d, 2H, *J* = 2.4 Hz), 2.56* (d, 2H, *J* = 2.8 Hz), 2.64–2.77 (bm, 1H), 2.87–3.02 (m, 1H), 3.19–3.30 (m, 2H), 4.36–4.52 (bm, 1H), 6.59* (d, 1H, *J =* 2.8 Hz), 6.66 (d, 1H, *J =* 2.9 Hz), 6.75–6.82 (m, 1H), 6.98–7.04 (m, 2H), 7.08 (d, 1H, *J* = 7.7 Hz), 7.20 (d, 1H, *J =* 9.0 Hz), 7.26 (dd, 1H, *J =* 8.7, 2.6 Hz), 7.31–7.39 (m, 1H), 8.22 (dd, 1H, *J =* 8.7, 2.5 Hz), 8.53–8.59 (m, 1H), 9.97 (exchangeable bs, 1H). ^13^C NMR (DMSO-*d*_6_, asterisk denotes isomer peaks) δ (ppm): 31.11*, 31.18, 31.77, 32.01*, 37.12, 37.18*, 40.90, 41.61*, 45.95*, 46.57, 111.66, 114.00, 117.12*, 117.24, 118.26, 119.00, 120.29 (*J* = 32.4 Hz), 121.96, 123.91 (q, *J* = 271.5 Hz), 126.04, 129.55, 130.23*, 130.29, 136.87*, 137.03, 137.56, 142.22, 145.36 (q, *J* = 4.1 Hz), 152.85, 156.51, 156.55*, 165.11*, 165.21, 165.62. HPLC analysis: retention time = 13.300 min; peak area, 95% (254 nm). HRMS: *m/z* for C_25_H_23_ClF_3_N_2_O_3_ [M + H]^+^ calculated: 491.13438, found: 491.13416.

##### 4.1.6.7 (4-Chloro-3-hydroxyphenyl)(4-(3-((5-(trifluoromethyl)pyridin-2-yl)oxy)benzyl)piperidin-1-yl)methanone (**10d**)

White solid, 54% yield from **35**. ^1^H-NMR (DMSO-*d*_6_) δ (ppm): 1.01–1.20 (bm, 2H), 1.44–1.88 (bm, 3H), 2.56 (d, 2H, *J* = 6.3 Hz), 2.60–2.79 (bm, 1H), 2.81–3.04 (bm, 1H), 3.46–3.68 (bm, 1H), 4.30–4.46 (bm, 1H), 6.73–6.79 (m, 1H), 6.91 (d, 1H, *J =* 2.0 Hz), 6.98–7.05 (m, 2H), 7.09 (d, 1H, *J =* 7.6 Hz), 7.20 (d, 1H, *J =* 8.8 Hz), 7.32–7.40 (m, 2H), 8.22 (dd, 1H, *J =* 8.9, 2.4 Hz), 8.54–8.60 (m, 1H), 10.50 (exchangeable bs, 1H). ^13^C NMR (DMSO-*d*_6_) δ (ppm): 31.30, 31.99, 37.26, 41.68, 47.15, 111.68, 114.86, 118.19, 119.01, 120.31 (q, *J* = 32.5 Hz) ,120.65, 121.96, 123.92 (q, *J* = 271.6 Hz), 126.04, 129.56, 129.86, 136.18, 137.59 (q, *J* = 3.2 Hz), 142.28, 145.36 (q, *J* = 4.3 Hz), 152.87, 153.00, 165.62, 167.83. HPLC analysis: retention time = 13.419 min; peak area, 98% (254 nm). HRMS: *m/z* for C_25_H_23_ClF_3_N_2_O_3_ [M + H]^+^ calculated: 491.13438, found: 491.13437.

##### 4.1.6.8 (4-Bromo-3-hydroxyphenyl)(4-(3-((5-(trifluoromethyl)pyridin-2-yl)oxy)benzyl)piperidin-1-yl)methanone (**10e**)

White solid, 46% yield from **36**. ^1^H-NMR (DMSO-*d*_6_) δ (ppm): 1.03–1.18 (bm, 2H), 1.47–1.89 (bm, 3H), 2.56 (d, 2H, *J* = 6.6 Hz), 2.61–2.76 (bm, 1H), 2.85–3.05 (bm, 1H), 3.50–3.64 (bm, 1H), 4.34–4.45 (bm, 1H), 6.70 (dd, 1H, *J =* 8.1, 1.9 Hz), 6.89 (d, 1H, *J =* 1.9 Hz), 6.89–7.05 (m, 2H), 7.09 (d, 1H, *J =* 7.4 Hz), 7.20 (d, 1H, *J* = 8.7 Hz), 7.36 (dd, 1H, *J* = 8.6, 7.7 Hz), 7.51 (d, 1H, *J =* 8.0 Hz), 8.22 (dd, 1H, *J =* 9.2, 2.6 Hz), 8.54–8.59 (m, 1H), 10.56 (exchangeable bs, 1H). ^13^C NMR (DMSO-*d*_6_) δ (ppm): 31.22, 32.04, 37.29, 41.71, 47.20, 110.32, 111.71, 114.53, 118.59, 119.05, 120.33 (q, *J* = 32.6 Hz), 121.99, 123.95 (q, *J* = 271.4 Hz), 126.07, 129.59, 132.91, 136.88, 137.62 (q, *J* = 3.0 Hz), 142.31, 145.38 (q, *J* = 4.4 Hz), 152.89, 154.07, 165.64, 165.65, 167.87. HPLC analysis: retention time = 13.548 min; peak area, 99% (254 nm). HRMS: *m/z* for C_25_H_23_BrF_3_N_2_O_3_ [M + H]^+^ calculated: 535.08387, found: 535.08398.

##### 4.1.6.9 (3-Hydroxyphenyl)(4-(3-((3-(trifluoromethyl)pyridin-2-yl)oxy)benzyl)piperidin-1-yl)methanone (**11a**)

White solid, 52% yield from **37**. ^1^H-NMR (DMSO-*d*_6_) δ (ppm): 1.02–1.20 (bm, 2H), 1.48–1.71 (bm, 2H), 1.72–1.88 (bm, 1H), 2.56 (d, 2H, *J* = 7.0 Hz), 2.62–2.75 (bm, 1H), 2.81–3.01 (bm, 1H), 3.48–3.67 (bm, 1H), 4.31–4.51 (bm, 1H), 6.67–6.71 (m, 1H), 6.73 (d, 1H, *J* = 7.5 Hz), 6.80 (dd, 1H, *J* = 8.3, 2.3 Hz), 6.95–7.01 (m, 2H), 7.09 (d, 1H, *J* = 7.6 Hz), 7.20 (t, 1H, *J* = 7.8 Hz), 7.29–7.37 (m, 2H), 8.22–8.38 (m, 1H), 8.34–8.40 (m, 1H), 9.65 (exchangeable bs, 1H). ^13^C NMR (DMSO-*d*_6_) δ (ppm): 31.32, 32.02, 37.36, 41.46, 41.69, 47.12, 112.59 (q, *J* = 32.9 Hz), 113.38, 116.17, 117.00, 118.80, 118.99, 121.91, 122.98 (q, *J* = 217.8 Hz), 125.95, 129.38, 129.51, 137.73, 137.82 (q, *J* = 4.8 Hz), 142.21, 151.58, 152.85, 157.24, 159.52, 168.77. HPLC analysis: retention time = 12.692 min; peak area, 97% (254 nm). HRMS: *m/z* for C_25_H_24_F_3_N_2_O_3_ [M + H]^+^ calculated: 457.17335, found: 457.17334.

##### 4.1.6.10 (3-Hydroxyphenyl)(4-(3-((4-(trifluoromethyl)pyridin-2-yl)oxy)benzyl)piperidin-1-yl)methanone (**11b**)

White solid, 61% yield from **38**. ^1^H-NMR (DMSO-*d*_6_) δ (ppm): 1.01–1.21 (bm, 2H), 1.48–1.72 (bm, 2H), 1.73–1.88 (bm, 1H), 2.56 (d, 2H, *J* = 6.9 Hz), 2.60–2.80 (bm, 1H), 2.82–3-01 (bm, 1H), 3.46–3.69 (bm, 1H), 4.29–4.49 (bm, 1H), 6.68–6.71 (m, 1H), 6.72 (dt, 1H, *J* = 7.4, 1.2 Hz), 6.80 (ddd, 1H, *J* = 8.2, 2.5, 1.0 Hz), 6.98–7.03 (m, 2H), 7.08 (d, 1H, *J* = 7.6 Hz), 7.20 (t, 1H, *J* = 7.8 Hz), 7.35 (tt, 1H, *J* = 8.7, 7.6 Hz), 7.38–7.41 (m, 1H), 7.46–7.50 (m, 1H), 8.40 (d, 1H, *J* = 5.2 Hz), 9.63 (exchangeable s, 1H). ^13^C NMR (DMSO-*d*_6_) δ (ppm): 31.27, 32.01, 37.21, 41.47, 41.69, 47.06, 107.68 (q, *J* = 3.9 Hz), 113.39, 114.27 (q, *J* = 3.2 Hz), 116.17, 116.97, 118.80, 121.81, 122.56 (q, *J* = 273.4 Hz), 125.84, 129.46, 129.50, 137.71, 140.28 (q, *J* = 33.5 Hz), 142.20, 149.51, 153.11, 157.26, 163.72, 168.78. HPLC analysis: retention time = 12.915 min; peak area, 98% (254 nm). HRMS: *m/z* for C_25_H_24_F_3_N_2_O_3_ [M + H]^+^ calculated: 457.17335, found: 457.17343.

##### 4.1.6.11 (3-Hydroxyphenyl)(4-(3-((6-(trifluoromethyl)pyridin-2-yl)oxy)benzyl)piperidin-1-yl)methanone (**11c**)

White solid, 60% yield from **39**. ^1^H-NMR (DMSO-*d*_6_) δ (ppm): 1.01–1.20 (bm, 2H), 1.46–1.87 (bm, 3H), 2.55 (d, 2H, *J* = 7.3 Hz), 2.60–2.78 (bm, 1H), 2.80–3.00 (bm, 1H), 3.50–3.64 (bm, 1H), 4.32–4.50 (bm, 1H), 6.67–6.76 (m, 2H), 6.80 (dd, 1H, *J* = 8.1, 1.7 Hz), 6.98–7.05 (m, 2H), 7.08 (d, 1H, *J* = 7.6 Hz), 7.20 (t, 1H, *J* = 7.8 Hz), 7.30 (d, 1H, *J* = 8.4 Hz), 7.36 (t, 1H, *J* = 7.9 Hz), 7.62 (d, 1H, *J* = 7.4 Hz), 8.11 (t, 1H, *J* = 7.9 Hz), 9.64 (exchangeable s, 1H). ^13^C-NMR (DMSO-*d*_6_) δ (ppm): 31.29, 31.96, 37.48, 41.49, 41.75, 47.07, 113.39, 115.62, 115.65, 116.16, 116.97, 118.29, 120.74 (q, *J* = 273.9 Hz), 121.75, 125.86, 129.46, 129.62, 137.72, 142.16, 142.24, 144.23 (q, *J* = 34.3 Hz), 152.88, 157.26, 162.99, 168.77. HPLC analysis: retention time = 12.843 min; peak area, 95% (254 nm). HRMS: *m/z* for C_25_H_24_F_3_N_2_O_3_ [M + H]^+^ calculated: 457.17335, found: 457.17355.

##### 4.1.6.12 (3-Hydroxyphenyl)(4-(4-((5-(trifluoromethyl)pyridin-2-yl)oxy)benzyl)piperidin-1-yl)methanone (**12**)

White solid, 29% yield from **55**. ^1^H-NMR (DMSO-*d*_6_) δ (ppm): 1.05–1.21 (bm, 2H), 1.47–1.61 (bm, 1H), 1.62–1.74 (bm, 1H), 1.75–1.88 (bm, 1H), 2.56 (d, 2H, *J* = 6.9 Hz), 2.63–2.79 (bm, 1H), 2.86–3.04 (bm, 1H), 3.49–3.69 (bm, 1H), 4.36–4.51 (bm, 1H), 6.68–6.72 (m, 1H), 6.74 (d, 1H, *J* = 7.6 Hz), 6.78–6.84 (m, 1H), 7.11 (d, 2H, *J* = 8.5 Hz), 7.17–7.22 (m, 2H), 7.25 (d, 2H, *J* = 8.5 Hz), 8.21 (dd, 1H, *J* = 8.9, 2.6 Hz), 8.52–8.59 (m, 1H), 9.67 (exchangeable bs, 1H). ^13^C NMR (DMSO-*d*_6_) δ (ppm): 31.38, 32.10, 37.46, 41.38, 47.12, 111.64, 113.38, 116.17, 117.01, 120.25 (q, *J* = 32.6 Hz), 121.25 (2C), 123.93 (q, *J* = 271.5 Hz), 129.52, 130.30 (2C), 137.15, 137.53 (q, *J* = 3.1 Hz), 137.75, 145.29 (q, *J* = 8.7 Hz), 151.04, 157.25, 165.68, 168.78. HPLC analysis: retention time = 12.933 min; peak area, 99% (254 nm). HRMS: *m/z* for C_25_H_24_F_3_N_2_O_3_ [M + H]^+^ calculated: 457.17335, found: 457.17331.

##### 4.1.6.13 (2-Fluoro-5-hydroxyphenyl)(4-(3-((4-(trifluoromethyl)pyridin-2-yl)oxy)benzyl)piperidin-1-yl)methanone (**13**)

Light-yellow solid, 66% yield from **40**. ^1^H-NMR (DMSO-*d*_6_) δ (ppm): 0.99–1.18 (m, 2H), 1.49–1.59 (m, 1H), 1.62–1.72 (m, 1H), 1.73–1.86 (m, 1H), 2.55 (d, 2H, *J* = 7.0 Hz), 2.70 (td, 1H, *J* = 12.6, 2.4 Hz), 2.91–3.03 (bm, 1H), 3.34–3.44 (m, 1H), 4.39–4.49 (m, 1H), 6.58–6.66 (m, 1H), 6.75–6.82 (m, 1H), 6.98–7.04 (m, 2H), 7.05–7.11 (m, 2H), 7.35 (dd, 1H, *J* = 8.6, 7.7 Hz), 7.38–7.42 (m, 1H), 7.48 (dd, 1H, *J* = 5.2, 0.9 Hz), 8.40 (d, 1H, *J* = 5.2 Hz), 9.65 (exchangeable bs, 1H). ^13^C NMR (DMSO-*d*_6_) δ (ppm): 31.23, 32.03, 37.20, 41.17, 41.61, 46.56, 107.72 (q, *J* = 3.9 Hz), 113.95, 114.31 (q, *J* = 3.3 Hz), 116.37 (d, *J* = 23.2 Hz), 117.09 (d, *J* = 7.7 Hz), 118.85, 121.85, 122.59 (q, *J* = 273.4 Hz), 124.91 (d, *J* = 20.4 Hz), 125.87, 129.53, 140.28 (q, *J* = 33.5 Hz), 142.18, 149.54, 150.70 (d, *J* = 233.2 Hz), 153.12, 153.73 (d, *J* = 1.9 Hz), 163.65, 163.74. HPLC analysis: retention time = 13.100 min; peak area, 98% (254 nm). HRMS: *m/z* for C_25_H_23_F_4_N_2_O_3_ [M + H]^+^ calculated: 475.16393, found: 475.16434.

### 4.2 MAGL Inhibition Assay

Human recombinant MAGL and 4-nitrophenylacetate (4-NPA) substrates were purchased from Cayman Chemical. IC_50_ values were generated in 96-well microtiter plates. The MAGL reaction was carried out at room temperature, at a final volume of 200 μL in 10 mM Tris buffer, pH 7.2, containing 1 mM EDTA, and 0.1 mg/mL bovine serum albumin (BSA). A total of 150 μL of 4-NPA 133.3 μM was added to 10 μL of DMSO containing the appropriate amount of compound. The reaction was started by adding 40 μL of MAGL (11 ng/well) so that the assay was linear over 30 min. The final concentration of the compounds analyzed ranged for from 320 to 0.02 nM. After 30 min from the start of the reaction, the absorbance values were measured using Victor X3 Microplates Reader (PerkinElmer) at 405 nm. Two reactions were also performed: one reaction containing no compounds and the second containing neither compound nor MAGL. IC_50_ values were derived from experimental data using the sigmoidal dose–response fitting of GraphPad Prism software. Final values were obtained from duplicates of three independent experiments. To remove possible false-positive results, a blank analysis was performed for each compound concentration, and the final absorbance results were obtained by subtracting the absorbance produced by the presence of all the components except MAGL under the same conditions. In the enzyme kinetics experiments, compound **13** was tested in the presence of scalar concentrations of 4-NPA. It was added in scalar amounts (concentration range = 10–1.25 nM) to a reaction mixture containing scalar concentrations of 4-NPA (15–1400 μM). Finally, the MAGL solution was added (11 ng/well). MAGL activity was measured by recording the increase in absorbance of 4-nitrophenol using Victor X3 Microplates Reader (PerkinElmer). The experimental data were analyzed by nonlinear regression analysis with GraphPad Prism software, using second order polynomial regression analysis and by applying the mixed model inhibition fit.

### 4.3 DTT Interference Assay

The inhibition assay was the same as described above, with the exception that prior to the addition of 40 μL of MAGL (11 ng/well), the compound–substrate mixture was incubated for 15 min in the presence of DTT at a 10 μM concentration.

### 4.4 MAGL Preincubation Assay

The MAGL reaction was conducted under the same conditions reported above. A total of 150 μL of MAGL (11 ng/well) was added to 10 μL of DMSO containing the appropriate amount of compound. After 0, 30, and 60 min of incubation time, the reaction was started by adding 40 μL of 4-NPA 500 μM. The enzyme activity was then measured after 30 min from the start of the reaction according to the procedure described above. Final values were obtained from triplicates of two independent experiments.

### 4.5 MAGL Dilution Assay

MAGL enzyme (880 ng in 75 μL of Tris buffer, pH 7.2) was incubated for 60 min at room temperature with 5 μL of compound **13** (concentration of 320 nM in the mixture) dissolved in DMSO. The MAGL–inhibitor mixture was then diluted 40-fold with the buffer. After 15 min of incubation, the reaction was started on a 160 μL aliquot by the addition of 40 μL of 4-NPA 500 μM and the enzyme activity was measured according to the procedure described above. Final values were obtained from triplicates of two independent experiments.

### 4.6 FAAH Inhibition Assay

Human recombinant FAAH and AMC arachidonoylamide substrates were purchased from Cayman Chemical. The FAAH reaction was carried out at room temperature (final volume of 200 μL in 125 mM Tris buffer, pH 9.0, containing 1 mM EDTA and 0.1 mg/mL BSA). A total of 150 μL of AMC arachidonoylamide (13.3 μM) (final concentration = 10 μM) was added to 10 μL of DMSO containing the appropriate amount of compound. The reaction was started adding 40 μL of FAAH (0.9 μg/well) so that the assay was linear over 30 min. The final concentration of the compounds analyzed ranged for from 200 to 0.0128 μM. After the reaction had proceeded for 30 min, fluorescence values were measured using a Victor X3 PerkinElmer instrument at an excitation wavelength of 340 nm and an emission of 460 nm. As for the MAGL assay, two reactions were also performed: one reaction containing no compound and the second one containing neither the inhibitor nor enzyme. IC_50_ values were derived from experimental data using the sigmoidal dose–response fitting of GraphPad Prism software. To remove possible false-positive results, a blank analysis was carried out for each compound concentration, and the final fluorescence results were obtained by subtracting the fluorescence produced by the presence of all the components except FAAH under the same conditions.

### 4.7 CB1 and CB2 Binding Assay

Binding assays to cannabinoid receptor 1 and 2 (CB1 and CB2) were performed as previously described.^8^ Briefly, clean membranes expressing *h*CB1 or *h*CB2 were resuspended in binding buffer (50 mM Tris–HCl, 2.5 mM EDTA, 5 mM MgCl_2_, 0.5% fatty acid-free BSA, pH 7.4) and incubated with vehicle or compounds and 0.5 nM [^3^H]CP55,940 for 90 min at 30 °C. Nonspecific binding was determined in the presence of 10 μM WIN55,512. After incubation, membranes were filtered through a pre-soaked 96-well microplate bonded with GF/B filters under vacuum and washed 12 times with 150 μL of ice-cold binding buffer. The radioactivity was measured, and the results are expressed as [^3^H]CP55,940 binding. Compound **13** was tested, at a screening concentration of 10 μM, in two independent experiments, each performed in triplicate.

### 4.8 Competitive Activity-Based Protein Profiling (ABPP)

A TAMRA-Fluorophosphonate serine hydrolase probe (TAMRA-FP, ActivX, Thermo Scientific) was used to fluorescently label serine hydrolases of mouse brain membrane preparations, while the binding of the TAMRA-FP was competed with different serine hydrolase inhibitors. Snap-frozen mouse half brains were homogenized each in 1 mL of extraction buffer (EB: 50 mM Tris–HCl, 3 mM MgCl_2_, 1 mM EGTA, pH 7.4) using a Mini-Bead Beater (BioSpec Products). The homogenate was centrifuged at 800*g* and 4 °C for 10 min. The supernatant was collected and kept on ice, while the pellet was resuspended in 1 mL of EB. The centrifugation with collection of the supernatant and resuspension was repeated four times. The collected supernatants were centrifuged at 16000*g* for 20 min at 4 °C. Afterward, the supernatants were discarded, and the pellets were resuspended with a syringe and needle (25G, 0.5 × 16 mm, NIPRO) and combined in 700 μL of 50 mM Tris–HCl (pH 7.4). The protein amount of the membrane preparation was accessed with a Pierce BCA protein assay (Thermo Scientific) according to the manufacturer’s instructions, and the membrane preparations were stored at −80 °C until use. For the ABPP, the mouse brain membrane preparations were diluted to 4 mg/mL in PBS and 19.5 μL was pre-incubated for 25 min at 25 °C with 0.5 μL of DMSO (vehicle control) or one of the following inhibitors (final concentration in brackets): **13** (10 μM, MAGL inhibitor), JZL184 (10 μM, MAGL inhibitor, Cayman Chemical Company), URB597 (4 μM, FAAH inhibitor, Cayman Chemical Company), WWL70 (10 μM, ABHD6 inhibitor, Cayman Chemical Company), THL (30 μM, ABHD6 and 12 inhibitor, Orlistat, Cayman Chemical Company) or MAFP (5 μM, serine hydrolase inhibitor, Abcam Biochemicals). TAMRA-FP (125 nM final concentration) was added to the samples and incubated for 25 min at 25 °C. The reaction was stopped by adding 6.5 μL of 4× Laemmli buffer with an incubation of 3 min at 25 °C followed by 10 min at 90 °C. The samples were cooled down, centrifuged for 1 min at 10000*g*, and separated by electrophoresis in a 12% SDS-polyacrylamide gel (120 V, 180 min). The fluorescent signal in the gel was recorded with a Typhoon FLA 9500 (GE Healthcare Bio-Sciences AB) in TAMRA settings. The comparability of the loaded protein amount was afterward confirmed by a Coomassie staining of the gel. The presented results were confirmed in two additional independent repetitions of the ABPP experiment.

### 4.9 Docking Calculations

The crystal structure of the *h*MAGL protein (5ZUN PDB code^30^) was taken from the Protein Data Bank.^50^ After adding hydrogen atoms, the protein was minimized using Amber20 software^51^ and the ff14SB force field at 300 K. The complex was placed in a rectangular parallelepiped waterbox; the TIP3P explicit solvent model for water was used, and the complex was solvated with a 10 Å water cap. Sodium ions were added as counterions to neutralize the system. Two steps of minimization were then carried out. In the first stage, we kept the protein fixed with a position restraint of 500 kcal/mol·Å^2^ and we solely minimized the positions of the water molecules. In the second stage, we minimized the entire system through 5000 steps of steepest descent followed by a conjugate gradient (CG) until a convergence of 0.05 kcal/Å mol. The energy minimized receptor was then used for the docking studies relative to compound **11b**, which were performed with AUTODOCK 4.0 software.^52^ The ligand was built with Maestro^53^ and then subjected to energy minimization performed with Macromodel^54^ until a convergence value of 0.05 kcal/Å mol, by employing the CG algorithm, MMFFs force field, and a distance-dependent dielectric constant of 1.0. AUTODOCK tools were used to automatically identify the torsion angles in the ligand, add the solvent model, and assign partial atomic charges to the protein and ligands (Kollmann and Gasteiger charges, respectively). The docking site used for calculations was defined in such a way as to contain all residues within a 10 Å shell from the reference ligand in the X-ray crystal structure. The energetic maps were calculated using a grid spacing of 0.375 Å and a distance-dependent function of the dielectric constant. The ligand was subjected to 200 runs of AUTODOCK search using the Lamarckian Genetic Algorithm, following a robust protocol,^29,55^ whose reliability was also tested with a self-docking study that produced an RMSD of 1.45 Å between the predicted and experimental conformations of the co-crystallized ligand in the reference X-ray structure. For each docking run, 10,000,000 steps of energy evaluations were performed, the number of individuals in the initial population was set to 500, and a maximum of 10,000,000 generations was simulated. An RMS cutoff of 2.0 Å was used for pose clustering. All other settings were left as their defaults. Clusters with a population lower than 10 conformers were not considered. For the docking calculations performed on compounds **7–9**, **10a-e**, **11a**, **11c**, **12**, **13**, and **40**, the same protocols of ligand construction, preparation, and docking were used, with the only exception that the average structure of the *h*MAGL-**11b** complex obtained after molecular dynamics simulations (see Section 4.3) was used as the receptor for docking studies and the best docked conformation belonging to the best cluster of solutions obtained (top-scored pose) was considered for each ligand.

### 4.10 MD Simulations

MD simulations were performed using Amber20^51^ and were carried out using the ff14SB force field. General Amber force field (GAFF) parameters were used for the ligand, whose partial charges were assigned using the Antechamber suite of Amber20, based on the AM1-BCC method. Each analyzed MAGL–ligand complex produced by docking was placed at the center of a rectangular parallelepiped box and solvated with a 15 Å water cap, generated using the TIP3P explicit solvent model. Sodium ions were then added to neutralize the obtained system, which was then energy-minimized using the same two-step protocol employed for the initial minimization of the receptor. The minimized complexes were used as input structures for the MD simulations, which were run using Particle Mesh Ewald (PME) electrostatics, a cutoff of 10 Å for the non-bonded interactions, and periodic boundary conditions. The SHAKE algorithm was used to constrain all bonds involving hydrogen atoms, and a time step of 2.0 fs was thus used for the simulation. Initially, a MD heating stage of 0.5 ns, in which the temperature of the system was raised from 0 to 300 K, was performed using constant-volume periodic boundary conditions. An initial equilibration stage of constant-pressure periodic boundary MD was run for 50 ns, keeping the temperature of the system at the constant value of 300 K with the Langevin thermostat. A second constant-pressure step of 500 ns was then performed at 300 K for optimal relaxation of the ligand–protein binding conformation and complex equilibration. In all these steps all α carbons of the protein were subjected to a harmonic potential of 10 kcal/mol Å^2^. Finally, a production step of 500 ns was performed, maintaining the same temperature and pressure conditions but removing any harmonic restraint, thus leaving the system totally free. In total, each analyzed complex was thus subjected to 1.05 μs of MD simulation. The final structures of the different MAGL–ligand complexes corresponded to the average of the last 500 ns of MD simulation minimized by the CG method until a convergence of 0.05 kcal/mol Å^2^. The average structures were obtained using the Cpptraj program^56^ implemented in Amber20, which was also used for RMSD and H-bond analyses.

### 4.11 Binding Energy Evaluations

The evaluation of the binding free energy associated with all *h*MAGL–ligand complexes analyzed through MD simulations was carried out using Amber20 as previously described.^57,58^ The trajectories relative to the last 500 ns of each simulation were extracted and used for the calculation, for a total of 500 snapshots (at time intervals of 1 ns). Van der Waals electrostatic and internal interactions were calculated with the SANDER module of Amber20, and the MOLSURF program was employed to estimate the nonpolar energies, while polar energies were calculated using the Poisson–Boltzmann methods with the MM-PBSA module of Amber20. A dielectric constant of 80 was used to represent the water phase in all calculations. For the gas phase, 10 different values of dielectric constant were used, ranging from 1 to 10. A total of 10 different binding free energy evaluations were thus performed for each of the analyzed ligand–protein complexes.

### 4.12 Evaluation of MAGL mRNA Expression in Human Normal and Tumor Tissues and Correlation with Survival in Pancreatic Cancer

The mRNA expression of MAGL was evaluated using the web-based genomics analysis and visualization platform GEPIA, analyzing the RNA sequencing expression data of 9736 tumors and 8587 normal samples from the TCGA and the GTEx projects.^45^ Moreover, we performed a correlation of MAGL mRNA expression with overall survival using TCGA-PAAD data of pancreatic cancer specimens (Tumor pancreatic adenocarcinoma TCGA dataset *178-rsem-tcgars)* in R2 (R2: Genomics analysis and Visualization Platform, http://r2.amc.nl).

### 4.13 Cell Culture

The pancreatic cancer cell line SUIT-2 (JCRB1094, Tokyo, Japan) and the primary cell cultures PDAC2 and PDAC3 were cultured in RPMI-1640 medium (Lonza, Basel, Switzerland) supplemented with 10% newborn calf serum, penicillin (50 IU/mL), and streptomycin (50 μg/mL) from Gibco (Gaithersburg, MD). In addition, the ductal immortalized normal hTERT-HPNE cells obtained from ATCC (Manassas, VA, USA) were cultured in DMEM medium with 5% FBS and 10 ng/mL human recombinant EGF. The cells were grown at 37 °C, 5% CO_2_ and were frequently tested for mycoplasma contamination with the MycoAlert Mycoplasma Detection Kit (Westburg, Leusden, The Netherlands).

### 4.14 Evaluation of *MAGL* mRNA Expression in Pancreatic Cancer Cells

RNA-sequencing analyses for PDAC2 and PDAC3 were performed, as described by Firuzi *et al*.^59^ Raw data were pre-processed for quality filtering and adapter trimming using the FASTX Toolkit (version 0.7) and subsequently mapped to the Human genome (GRCh38) using the STAR alignment tool (version 2.5.3a). We obtained ∼90% of reads mapped to the Human Genome per sample. Gene counts in fragments per kilobase of transcript per million mapped reads (FPKM) normalization were computed using the CuffLinks algorithm, and plots were generated with R version 3.5.0. SUIT-2 mRNA expression data was obtained from the Cancer Cell Line Encyclopedia (https://portals.broadinstitute.org/ccle).

### 4.15 Drugs and Chemicals

Gemcitabine was a generous gift from Eli-Lilly (Indianapolis, IN) and was dissolved in sterile water, while MAGL inhibitors were solubilized in DMSO and diluted in culture medium before use. All other chemicals were purchased from Sigma-Aldrich (Zwijndrecht, The Netherlands).

### 4.16 Growth Inhibition Studies

The inhibitory effects on cell growth were evaluated by sulforhodamine B (SRB) assay. Cells were seeded in 96 well plates at a density of 5000 per well. After 24 h, once the cell monolayer was formed, cells were treated for 72 h with MAGL inhibitors (0.1–50 μM) or gemcitabine (1–1250 nM). Cells were then incubated for 72 h, fixed with trichloroacetic acid at 4 °C, washed with deionized water, and then dried at RT. After the fixation, the plate was stained with SRB, washed with acetic acid solution, and left to dry again. SRB was resuspended in a Tris base solution and its absorption was measured at 490 and 540 nm, as described previously.^60^ Finally, the half-maximal response concentration (IC_50_) was calculated with GraphPad Prism version 9 (GraphPad PRISM, Intuitive Software for Science, San Diego, CA).

### 4.17 Analysis of Cell Migration

Pancreatic cancer cells were seeded in a 96 well plate at a density of 25,000 cells/well to form a confluent monolayer after 24 h. Subsequently, the monolayer was wounded by a 96-well pin tool scratcher. Detached cells were washed away with phosphate-buffered saline (PBS). Medium only or medium containing a concentration of 5 times (5×) the IC_50_ of each drug was added to the wells: 36 μM for **13**, 14 μM for JZL-184, and 17 μM for ABX-1431. Bright-field images were taken with the software Universal Grab 6.3 digital on a Leica DMI300B microscope (Leica Microsystems, Eindhoven, The Netherlands) at different time points, to be analyzed with Scratch Assay 6.2 software (Digital Cell imaging Labs, Keerbergen, Belgium) as described previously.^60^

### 4.18 Apoptosis Assays

First, cells were seeded in a 96 well plate at a density of 5000 per well. After 24 h, cells were treated with drugs at the concentration of IC_50_ for 72 h. At the end of treatment, cells were fixed with paraformaldehyde, washed, and stained with annexin V/FITC (Apoptest, VPS Diagnostic, Hoeven, the Netherlands) in binding buffer BBA (10 mM HEPES/NaOH pH 7.4, 140 mM NaCl, and 2.5 mM CaCl2). After a wash with BBA, the fluorescence signal was measured by a plate reader (BioTek Instruments Inc., Winooski, VT) with excitation and emission at 485 and 535 nm, respectively. The values were normalized on cells number stained by crystal violet solution (for a solution of 100 mL: 750 mg of violet crystal powder, 250 mg of NaCl, 4.7 mL of 37% formaldehyde, 50 mL of ethanol and 45.3 mL of bidistilled water). The dye was solubilized in PBS containing 1% SDS and measured photometrically at 595 nm absorbance. Then, to assess whether caspase-3, an enzyme involved in the effector phase of apoptosis, is a downstream target of MAGL inhibitors, its enzymatic activity was measured by a specific spectrofluorimetric activity assay (Human Active Caspase-3 Immunoassay Quantikine ELISA, Catalog Number KM300, R&D Systems, Inc., Minneapolis, MN)**.** Briefly, cells were plated in 6-well plates (5 × 10^5^ cells/ml) and exposed to the drugs for 24 h at 5× IC_50_ or for 72 h at their IC_50_. At the end of drug incubation, cell extracts were diluted and mixed to the reagents according to the manufacturers’ protocol. Absorbance was measured at 450 nm, subtracting readings at 540 nm. Relative caspase activity was calculated using a standard curve with human recombinant caspase-3.

### 4.19 Evaluation of Pharmacological Interaction with Gemcitabine

The pharmacological interaction between compound **13** and gemcitabine was evaluated by the median drug effect analysis method, as described previously.^61^ Compound **13** was added at the inhibitory concentration of 50%, while gemcitabine was added in a drug range between 0 and 1250 nM. The combination index (CI) was calculated to compare cell growth inhibition of the combination and each drug alone. Data analysis was carried out using CalcuSyn software (Biosoft, Oxford, UK). A CI of below 0.8 indicates a synergetic cytotoxic effect. A CI between 0.8 and 1.2 indicates an additive effect and above 1.2 indicates an antagonistic effect of the combination therapy.

### 4.20 PCR Assays to Evaluate Key Determinants in Migration, Apoptosis Induction, and Gemcitabine Activity

Real-time quantitative reverse transcription PCR (qRT-PCR) was performed to evaluate the gene expression of MMP9, BCL-2, BCL-x, and *h*ENT1, using β*-actin*, and *GAPDH* as housekeeping genes. The cells were seeded at 3 × 10^3^ to 5 × 10^3^ in a 6-well flat bottom plate with 2 mL medium per well and incubated with drugs at 5× IC_50_ for 24 h. Total RNA was extracted using the TRIzol Reagent (15596–026, ThermoFisher Scientific, Waltham, MA) according to the manufacturer’s protocol. One microgram of RNA was reverse transcribed using first-strand cDNA synthesis (First Strand cDNA Synthesis Kit; ThermoFisher #K1612) on a Bio-Rad machine C100 Thermal Cycler. Real-time qPCR quantification was performed using specific TaqMan detection probes and primers (TaqMan Universal PCR Master Mix #4304437; Thermo Fisher Scientific, USA) with the ABIPRISM-7500 instrument (Applied Biosystems, Foster City, CA), as described previously.^60^ Data obtained were analyzed according to the 2^–ΔΔ*Ct*^ method.

### 4.21 Statistics

All experiments were performed in triplicate and repeated at least twice. Data were expressed as mean values ± standard deviation (SD) or standard error of the mean (SEM) and analyzed by Student’s *t* test or ANOVA performed by GraphPad Prism 9 software. The level of significance was *p* < 0.05.

### 4.22 *In Vitro* ADME Assays

#### 4.22.1 Chemicals

All solvents and reagents were from Sigma-Aldrich Srl (Milan, Italy). Dodecane was purchased from Fluka (Milan, Italy). Pooled male donors 20 mg/mL HLMs were from BD Gentest-Biosciences (San Jose, California). Milli-Q quality water (Millipore, Milford, MA, USA) was used. Hydrophobic filter plates (MultiScreen-IP, clear plates, 0.45 mm diameter pore size), 96-well microplates, and 96-well UV-transparent microplates were obtained from Millipore (Bedford, MA, USA).

#### 4.22.2 UV/LC–MS Methods

LC analyses for water solubility were performed by an LC–MS/MS system consisting of a Varian apparatus (Varian Inc) including a vacuum solvent degassing unit, two pumps (212-LC), a Triple Quadrupole MSD (Mod. 320-LC) mass spectrometer with ES interface, and Varian MS Workstation System Control Vers. 6.9 software. Chromatographic separation was obtained using a Kinetex C18 column (150 × 4.6 mm) with a 5 μm particle size and gradient elution with a binary solution; (eluent A: ACN, eluent B: water, both eluents were acidified with formic acid 0.1% v/v). The analysis started with 5% of A (from *t* = 0 to *t* = 1 min), then A was increased to 95% (from *t* = 1 to *t* = 10 min), then kept at 95% (from *t* = 10 to *t* = 19 min), and finally returned to 5% of eluent A in 1.0 min. The flow rate was 0.6 mL/min, and injection volumes were 10 μL. The instrument operated in positive mode, and parameters were detector 1450 V, drying gas pressure 35.0 psi, desolvation temperature 300.0 °C, nebulizing gas 45.0 psi, needle 5550 V and shield 350 V. Nitrogen was used as a nebulizer gas and drying gas. Collision-induced dissociation was performed using argon as the collision gas at a pressure of 1.8 mTorr in the collision cell. The transitions as well as the capillary voltage and the collision energy used are appropriated for each tested compound. Quantification of the single compound was made by comparison with appropriate calibration curves realized with standard solutions in methanol. LC analyses of PAMPA, metabolic stability, and stability tests (in MeOH, PBS, human plasma) were performed by UV/LC–MS with an Agilent 1100 LC/MSD VL system ((G1946C) Agilent Technologies, Palo Alto, CA) using a Phenomenex Kinetex C18–100 Å (150 × 4.6 mm, 5 μm particle size) at room temperature. Analyses were carried out with the same chromatographic conditions reported above.

#### 4.22.3 Water Solubility

Solid **5c** and **13** (1 mg) were added to 1 mL of distilled water. Each sample was mixed at room temperature in a shaker water bath for 24 h.^62^ The resulting suspension was filtered through a 0.45 μm nylon filter (Acrodisc), and the solubilized compound was quantified in triplicate using the LC–MS/MS method reported above, by comparison with the appropriate calibration curve that was obtained from samples of the compound dissolved in methanol at different concentrations.

#### 4.22.4 Parallel Artificial Membrane Permeability Assay (PAMPA)

Each “donor solution” was prepared from a solution of the appropriate compound (DMSO, 1 mM) diluted with phosphate buffer (pH 7.4, 0.025 M) up to a final concentration of 0.5 mM. The donor wells were filled with 150 μL of “donor solution”. The filters were coated with 10 μL of 1% (w/v) dodecane solution of phosphatidylcholine, and the lower wells were filled with 300 μL of “acceptor solution” (50% v/v DMSO and phosphate buffer). The sandwich plate was assembled and incubated for 5 h at room temperature with gentle shaking. After the incubation time, plates were separated and the amount of compound in both the donor and acceptor wells was measured by UV/LC–MS. For each compound, the determination was performed in three independent experiments. Permeability (*P*_app_) was calculated according to the following equation obtained from the literature equation^63,64^ with some modification in order to obtain permeability values in cm/s:
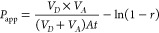
where *V*_A_ is the volume in the acceptor well (cm^3^), *V*_D_ is the volume in the donor well (cm^3^), *A* is the “effective area” of the membrane (cm^2^), *t* is the incubation time (s), and *r* is the ratio between drug concentration in the acceptor and equilibrium concentration of the drug in the total volume (*V*_D_ + *V*_A_). Drug concentration was estimated by using the peak area integration. Membrane retentions (%MR) were calculated according to the following equation:
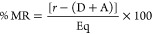
where *r* is the ratio between drug concentration in the acceptor and equilibrium concentration, and D, A, and Eq represent drug concentration in the donor, acceptor, and equilibrium solutions, respectively.

#### 4.22.5 Metabolic Stability in HLMs (Human Liver Microsomes)

DMSO solutions of **5c** and **13** were incubated at 37 °C for 1 h in presence of human liver microsomes (0.2 mg/mL, 5 μL), an NADPH regenerating system (NADPH 0.2 mM, NADPH^+^ 1 mM, d-glucose-6-phosphate 4 mM, 4 unit/mL glucose-6-phosphate dehydrogenase and MgCl_2_ 48 mM), and phosphate buffer (pH 7.4, 25 mM, up to a final volume of 500 μL). The reaction was cooled down in ice and quenched by adding acetonitrile (1.0 mL). After centrifugation (4000 rpm for 10 min), the supernatant was taken, dried under nitrogen flow, and suspended in 100 μL of methanol and the parent drug and metabolites were subsequently determined by UV/LC–MS. The percentage of nonmetabolized compounds was calculated by comparison with reference solutions. For each compound, the determination was performed in three independent experiments.

#### 4.22.6 Stability Test

##### 4.22.6.1 In Polar Solvents

Each compound was dissolved at RT in MeOH or PBS (25 mM, pH 7.4) up to a final concentration of 500 μM. Aliquot samples (20 μL) were taken at fixed time points (0.0, 4.0, 8.0, and 24.0 h) and were analyzed by UV/LC–MS. For each compound, the determination was performed in three independent experiments.

##### 4.22.6.2 In Human Plasma

The incubation mixture (total volume of 2.0 mL) was constituted by the following components: pooled human plasma (1.5 mL, 55.7 mg protein/mL),^65^ HEPES buffer (1.4 mL, 25 mM, 140 mM NaCl pH 7.4), and 100 μL of each compound in DMSO (3.0 mM). The solution was mixed in a test tube that was incubated at 37 °C. At set time points (0.0, 0.25, 0.50, 1.0, 2.0, 4.0, 8.0, and 24.0 h), samples of 100 μL were taken, mixed with 400 μL of cold acetonitrile, and centrifuged at 5000 rpm for 15 min.^66^ The supernatant was removed and analyzed by UV/LC–MS. For each compound, the determination was performed in three independent experiments.

## Supporting Information Available

RMSD analysis of ligand disposition in the MAGL-**11b** complexes during the MD; MM-PBSA results for the eight different MAGL-**11b** complexes; correlation of ligand activity and binding energy obtained using different ε_int_ values; activities and best correlated binding energy values predicted for the analyzed ligands; IC_50_ values of compound **13** toward CB1, CB2, and FAAH; MM-PBSA (ε_int_ = 4) results for MAGL-**11b** and MAGL-**13** complexes; minimized average structures of MAGL in complex with **7** and **12**; RP-HPLC traces of the final compounds; ^1^H and ^13^C-NMR spectra of the final compounds; ESI-HRMS spectra of the final compounds; analysis of the mechanism of MAGL inhibition of JZL-184; inhibition of the activity of MAGL and competitive binding of compound **13**; minimized average structures of MAGL in complex with **11b** superimposed with **5b**; minimized average structures of MAGL in complex with **13** (PDF)
Molecular formula strings and associated biochemical data for the reported compounds (CSV)
PDB ID of the crystal structure of MAGL (PDB)

## Supplementary Material

jm1c01806_si_002.csv

jm1c01806_si_003.pdb

## ABBREVIATIONS

2-AG: 2-arachidonoylglycerol
4-NPA: 4-nitrophenylacetate
9-BBN: 9-borabicyclo[3.3.1]nonane
AA: arachidonic acid
ABHD6: α/β hydrolase-6
ABHD12: α/β hydrolase-12
ABPP: activity-based protein profiling
AEA: anandamide
BSA: bovine serum albumin
CB: cannabinoid receptor
CG: conjugate gradient
DIPEA: *N*,*N*-diisopropylethylamine
DMF: *N*,*N*-dimethylformamide
DTT: 1,4-dithio-dl-threitol
eCBs: endocannabinoids
FAAH: fatty acid amide hydrolase
FPKM: fragments per kilobase million
GAFF: general Amber force field
HATU: 1-[bis(dimethylamino)methylene]-1*H*-1,2,3-triazolo[4,5-*b*]pyridinium 3-oxide hexafluorophosphate
*h*ENT1: human equilibrative nucleoside transporter 1
MAGL: monoacylglycerol lipase
MD: molecular dynamics
MM-PBSA: molecular mechanics Poisson–Boltzmann surface area
MMP9: matrix metalloproteinase 9
PAAD: pancreatic adenocarcinoma
PDAC: pancreatic ductal adenocarcinoma
PME: Particle Mesh Ewald
RMSD: root-mean square deviation
SRB: sulforhodamine B
TBAI: tetrabutylammonium iodide
