# Supplementary material for: Reversible Monoacylglycerol Lipase Inhibitors: Discovery of a New Class of Benzylpiperidine Derivatives
Source: J Med Chem. 2022 May 6;65(10):7118–40. doi: 10.1021/acs.jmedchem.1c01806 (PMC9150076; doi:10.1021/acs.jmedchem.1c01806)
Supplement: Supplementary file 1 — jm1c01806_si_001.pdf [file jm1c01806_si_001.pdf]

Supporting Information

Reversible monoacylglycerol lipase (MAGL) inhibitors: discovery of a new class of benzylpiperidine derivatives

Giulia Bononi[a], Miriana Di Stefano[a,b], Giulio Poli[a], Gabriella Ortore[a], Philip Meier[c], Francesca Masetto[d], Isabella Caligiuri[e], Flavio Rizzolio[e,f], Marco Macchia[a], Andrea Chicca[c], Amir Avan[g], Elisa Giovannetti[d,h], Chiara Vagaggini[i], Annalaura Brai[i], Elena Dreassi[i], Massimo Valoti[b], Filippo Minutolo[a,j], Carlotta Granchi[a,j,], Jürg Gertsch[c], Tiziano Tuccinardi[a,j].*

[a] Department of Pharmacy, University of Pisa, Via Bonanno 6, 56126 Pisa, Italy.

[b] Department of Life Sciences, University of Siena, Via Aldo Moro, 2, 53100 Siena, Italy.

[c] Institute of Biochemistry and Molecular Medicine, NCCR TransCure, University of Bern, CH-3012 Bern, Switzerland.

[d] Department of Medical Oncology, VU University Medical Center, Cancer Center Amsterdam, DeBoelelaan 1117, 1081HV, Amsterdam, The Netherlands.

[e] Pathology Unit, Centro di Riferimento Oncologico di Aviano (CRO) IRCCS, 33081 Aviano, Italy.

[f] Department of Molecular Sciences and Nanosystems, Ca' Foscari University, 30123 Venezia, Italy.

[g] Metabolic Syndrome Research Center, Mashhad University of Medical Science, Mashhad 91886-17871, Iran.

[h] Cancer Pharmacology Lab, Fondazione Pisana per la Scienza, via Giovannini 13, 56017 San Giuliano Terme, Pisa, Italy.

[i] Department of Biotechnology, Chemistry and Pharmacy, University of Siena, 53100 Siena, Italy.

[j] Center for Instrument Sharing of the University of Pisa (CISUP), Lungarno Pacinotti 43, 56126 Pisa, Italy.

Corresponding author: Carlotta Granchi, carlotta.granchi@unipi.it

Table of Contents

Table S1. RMSD analysis of ligand disposition in the MAGL-**11b** complexes during the MD. S3

Table S2. MM-PBSA results for the eight different MAGL-**11b** complexes. S4

Table S3. Correlation of ligand activity and binding energy obtained using different ε_{int} values. S5

Table S4. Activities and best correlated binding energy values predicted for the analyzed ligands. S6

Table S5. IC_{50} values of compound **13** towards CB1, CB2 and FAAH. S7

Table S6. MM-PBSA (ε_{int} = 4) results for MAGL-**11b** and MAGL-**13** complexes. S7

Figure S1. Minimized average structures of MAGL in complex with **7** and **12**. S8

Figure S2-S15. RP-HPLC traces of the final compounds. S9-S22

Figure S16-S29. ^{1}H and ^{13}C-NMR NMR spectra of the final compounds. S23-S36

Figures S30-S43. ESI-HRMS spectra of the final compounds. S37-S43

Figure S44. Analysis of the mechanism of MAGL inhibition of JZL-184. S44

Figure S45. Inhibition of the activity of MAGL and competitive binding of compound **13**. S44

Figure S46. Minimized average structures of MAGL in complex with **11b** superimposed with **5b**. S45

Figure S47. Minimized average structures of MAGL in complex with **13**. S45

Table S1. Average RMSD of the ligand disposition, with respect to the initial coordinates, observed for the eight *h*MAGL-**11b** complexes during the last 500 ns of MD simulation.

**Binding pose**	**Mean RMSD (Å)**
**1**	3.8
**2**	5.6
**3**	1.9
**4**	3.8
**5**	6.3
**6**	4.5
**7**	5.0
**8**	3.0

Table S2. MM-PBSA results for the eight different *h*MAGL-**11b** complexes.

	VDW	EEL	EPB	ENPOLAR	EDISPER	ΔPBSA[a]
**Pose 1**	-58.6	-11.0	48.8	-42.5	74.8	11.5
**Pose 2**	-58.2	-11.9	47.6	-43.5	74.1	8.1
**Pose 3**	-55.6	-26.2	46.4	-41.2	67.6	-8.9
**Pose 4**	-50.7	-25.9	47.1	-38.4	65.2	-2.7
**Pose 5**	-55.5	-24.6	46.3	-40.8	69.3	-5.3
**Pose 6**	-58.7	-19.7	48.2	-43.4	73.3	-0.35
**Pose 7**	-53.0	-23.8	49.0	-41.1	69.6	0.67
**Pose 8**	-56.4	-27.6	50.7	-42.9	72.9	-3.2

VDW, Van der Waals molecular mechanics energy; EEL, Electrostatic molecular mechanics energy; EPB, Polar contribution to the solvation energy; ENPOLAR, Nonpolar contribution of repulsive solute-solvent interactions to the solvation energy; EDISPER, Nonpolar contribution of attractive solute-solvent interactions to the solvation energy.

[a] ΔPBSA is the sum of the electrostatic (EEL) and van der Waals (VDW) as well as polar (EPB) and nonpolar (ENPOLAR, EDISPER) solvation free energies. Data are expressed as kcal/mol.

Table S3. R^2 values obtained for the correlation between the activity of the 14 analyzed benzylpiperidine ligands, expressed as pIC_{50} values, and the corresponding binding free energies calculated using the MM-PBSA method with different internal dielectric constant (ε_{int}) values.

ε_{int}	R^2
**1**	0.53
**2**	0.70
**3**	0.78
**4**	0.79
**5**	0.78
**6**	0.76
**7**	0.75
**8**	0.72
**9**	0.72
**10**	0.71

Table S4. Binding free energy values calculated for the 14 analyzed benzylpiperidine ligands using the MM-PBSA method with internal dielectric constant ε_{int} = 4. Values are expressed in kcal/mol. The corresponding activities of the ligands expressed as pIC_{50} values are reported.

Compound	Activity (pIC_{50})	ΔPBSA (kcal/mol)
**7**	6.87	-22.1
**8**	6.86	-22.1
**9**	6.83	-23.4
**10a**	7.58	-24.4
**10b**	7.07	-23.0
**10c**	6.90	-22.8
**10d**	6.97	-23.5
**10e**	6.96	-23.8
**11a**	6.76	-23.6
**11b**	7.88	-24.8
**11c**	7.44	-23.4
**12**	6.06	-20.6
**13**	8.69	-25.4
**40**	5.82	21.2

Table S5. Pharmacological characterization of compound **13** towards cannabinoid receptors and FAAH.

	**IC_{50} values (μM)**		
**Compound**	**CB1**	**CB2**	**FAAH**
**13**	> 10	> 10	> 10

Table S6. MM-PBSA (ε_{int} = 4) results for *h*MAGL-**11b** and *h*MAGL-**13** complexes.

**11b**	**POLAR**		**NON POLAR**			**ΔPBSA[a]** -24.8
	4.3		-29.1			
	**EEL**	**EPB**	**VDW**	**ENPOLAR**	**EDISPER**	
	-6.5	10.9	-55.6	-41.2	67.6	
**13**	**POLAR**		**NON POLAR**			**ΔPBSA[a]** -25.4
	3.8		-29.2			
	**EEL**	**EPB**	**VDW**	**ENPOLAR**	**EDISPER**	
	-7.4	11.2	-56.5	-41.1	68.4	

EEL, Electrostatic molecular mechanics energy; EPB, Polar contribution to the solvation energy; VDW, Van der Waals molecular mechanics energy; ENPOLAR, Nonpolar contribution of repulsive solute-solvent interactions to the solvation energy; EDISPER, Nonpolar contribution of attractive solute-solvent interactions to the solvation energy.

[a] ΔPBSA is the sum of the electrostatic (EEL) and van der Waals (VDW) as well as polar (EPB) and nonpolar (ENPOLAR, EDISPER) solvation free energies. Data are expressed as kcal/mol.

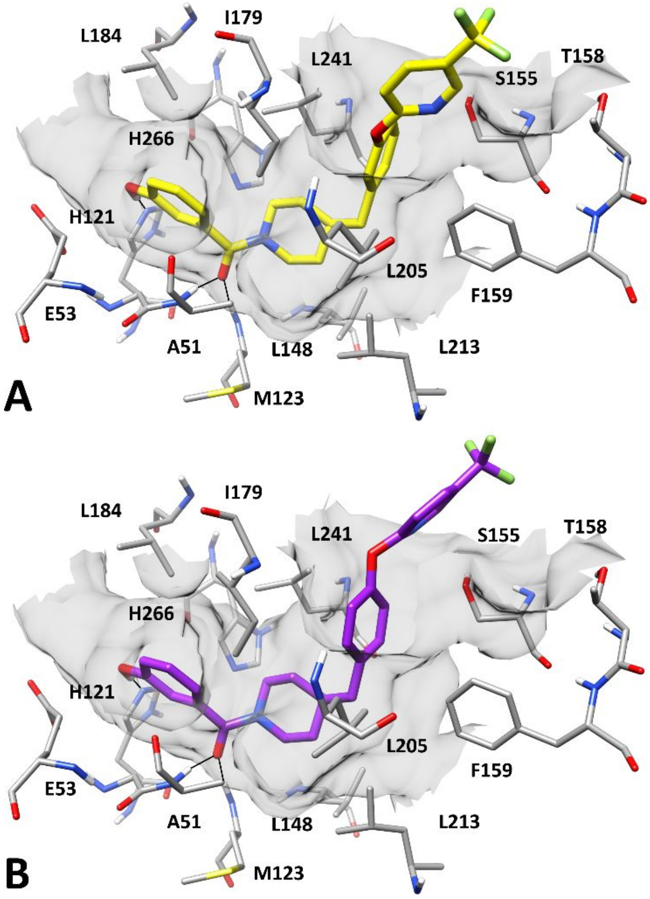

Figure S1. Minimized average structure of *h*MAGL in complex with A) compound **7** (yellow) and B) compound **12** (purple). In both panels, the inner surface of MAGL binding site is shown in gray, while ligand-protein H-bonds are shown as black lines.

Sample Name : MDS22_500uM
Sample ID : MDS22_500uM
Data Filename : MDS22_500uM_p.lcd
Method Filename : MAGL254.lcm

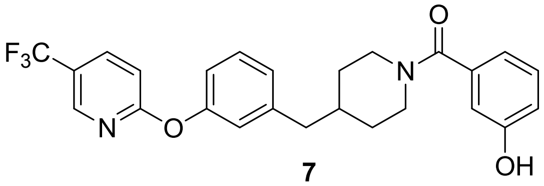

7

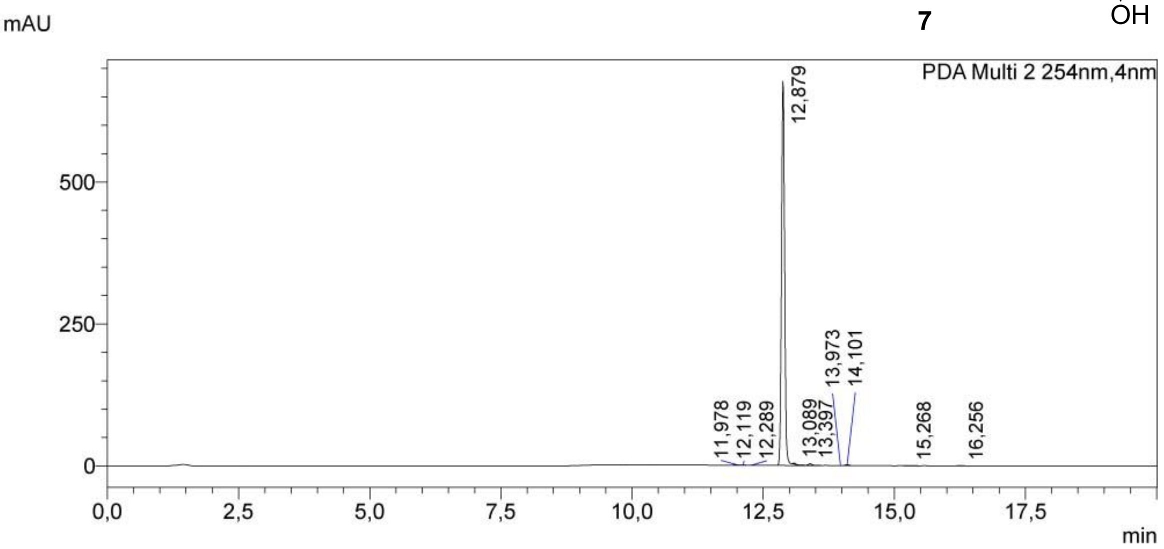

PDA Ch2 254nm

Peak#	Ret. Time	Area	Area%	Height
1	11,978	8410	0,300	1599
2	12,119	4016	0,143	523
3	12,289	2718	0,097	396
4	12,879	2758402	98,260	676596
5	13,089	5045	0,180	1658
6	13,397	10804	0,385	2508
7	13,973	1756	0,063	264
8	14,101	9907	0,353	1699
9	15,268	3063	0,109	328
10	16,256	3140	0,112	527
Total		2807262	100,000	686098

Figure S2. HPLC chromatogram of compound **7**.

Sample Name : LB10_500uM
Sample ID : LB10_500uM
Data Filename : LB10_500uM_p2.lcd
Method Filename : MAGL254.lcm

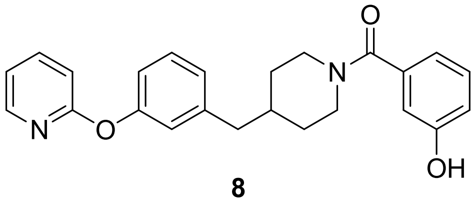

8

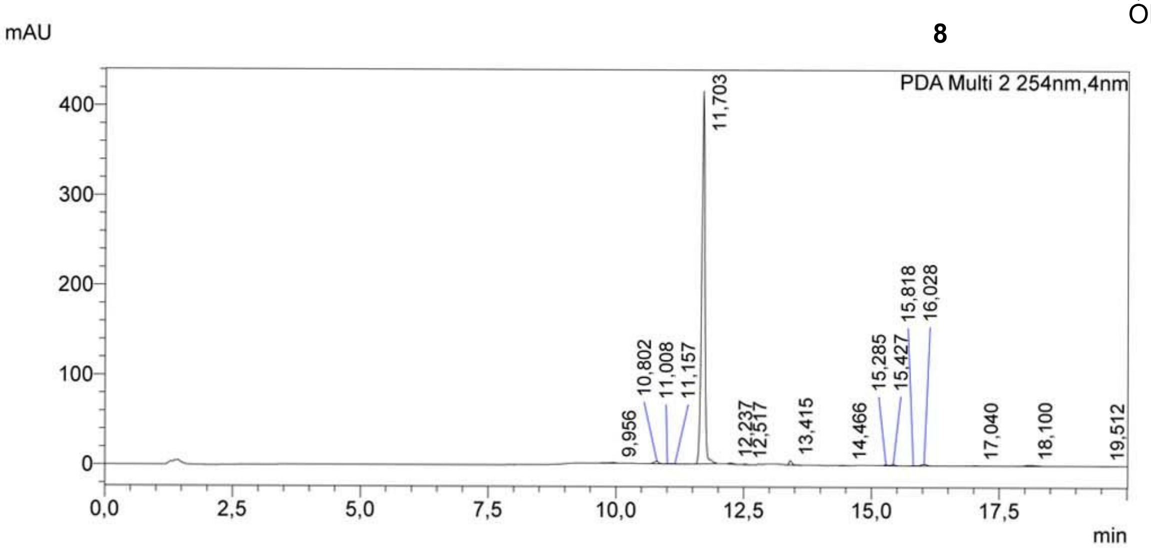

PDA Ch2 254nm

Peak#	Ret. Time	Area	Area%	Height
1	9,956	6692	0,336	733
2	10,802	16862	0,847	2829
3	11,008	1189	0,060	188
4	11,157	1379	0,069	213
5	11,703	1888125	94,798	415217
6	12,237	5859	0,294	1092
7	12,517	1936	0,097	553
8	13,415	18855	0,947	4867
9	14,466	1195	0,060	155
10	15,285	4477	0,225	847
11	15,427	5969	0,300	1125
12	15,818	2508	0,126	397
13	16,028	14403	0,723	1735
14	17,040	3800	0,191	427
15	18,100	16920	0,850	1143
16	19,512	1563	0,078	147
Total		1991733	100,000	431667

Figure S3. HPLC chromatogram of compound **8**.

Sample Name : CG1624_500uM
Sample ID : CG1624_500uM
Data Filename : CG1624_500uM_p2.lcd
Method Filename : MAGL254.lcm

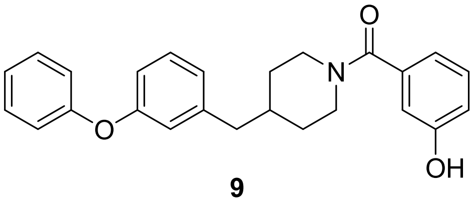

9

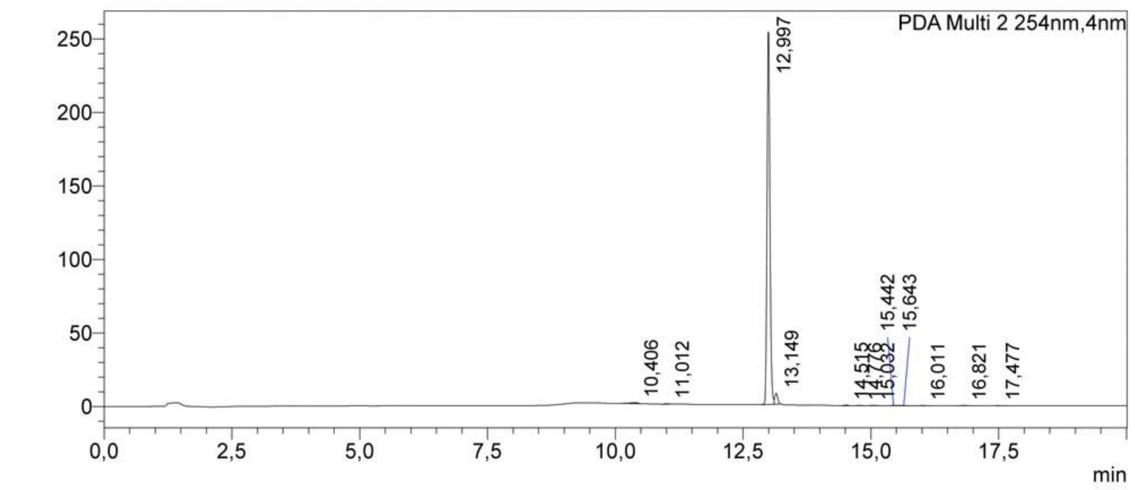

PDA Ch2 254nm

Peak#	Ret. Time	Area	Area%	Height
1	10,406	7882	0,779	687
2	11,012	2207	0,218	285
3	12,997	960673	94,974	253256
4	13,149	27019	2,671	7436
5	14,515	2148	0,212	462
6	14,776	1304	0,129	213
7	15,032	2484	0,246	250
8	15,442	2529	0,250	311
9	15,643	1407	0,139	197
10	16,011	1158	0,114	193
11	16,821	1653	0,163	193
12	17,477	1041	0,103	150
Total		1011507	100,000	263632

Figure S4. HPLC chromatogram of compound **9**.

Sample Name : MDS24_500uM
Sample ID : MDS24_500uM
Data Filename : MDS24_500uM_p.lcd
Method Filename : MAGL254.lcm

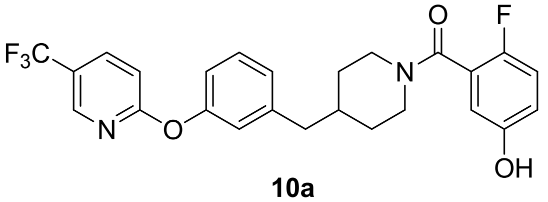

10a

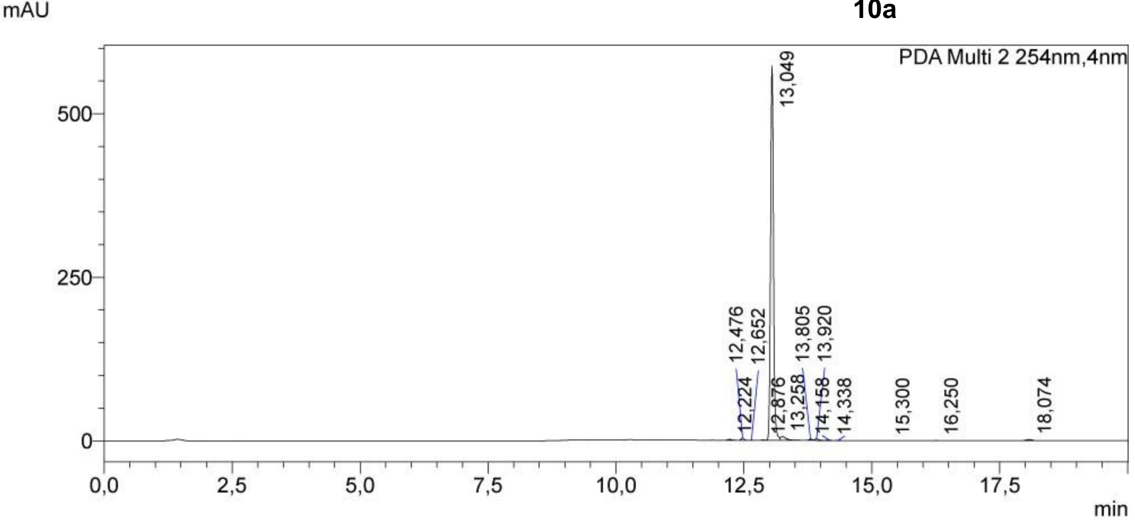

PDA Ch2 254nm

Peak#	Ret. Time	Area	Area%	Height
1	12,224	10442	0,433	1651
2	12,476	15737	0,652	3591
3	12,652	1467	0,061	274
4	12,876	3282	0,136	764
5	13,049	2280625	94,494	571979
6	13,258	51870	2,149	5690
7	13,805	10752	0,445	2042
8	13,920	13577	0,563	2371
9	14,158	4425	0,183	939
10	14,338	1756	0,073	335
11	15,300	1889	0,078	209
12	16,250	2033	0,084	305
13	18,074	15669	0,649	1713
Total		2413524	100,000	591863

Figure S5. HPLC chromatogram of compound **10a**.

Sample Name : MDS26_500uM
Sample ID : MDS26_500uM
Data Filename : MDS26_500uM_p.lcd
Method Filename : MAGL254.lcm

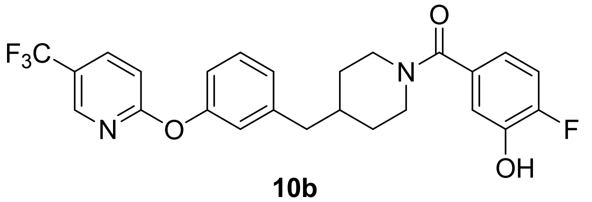

10b

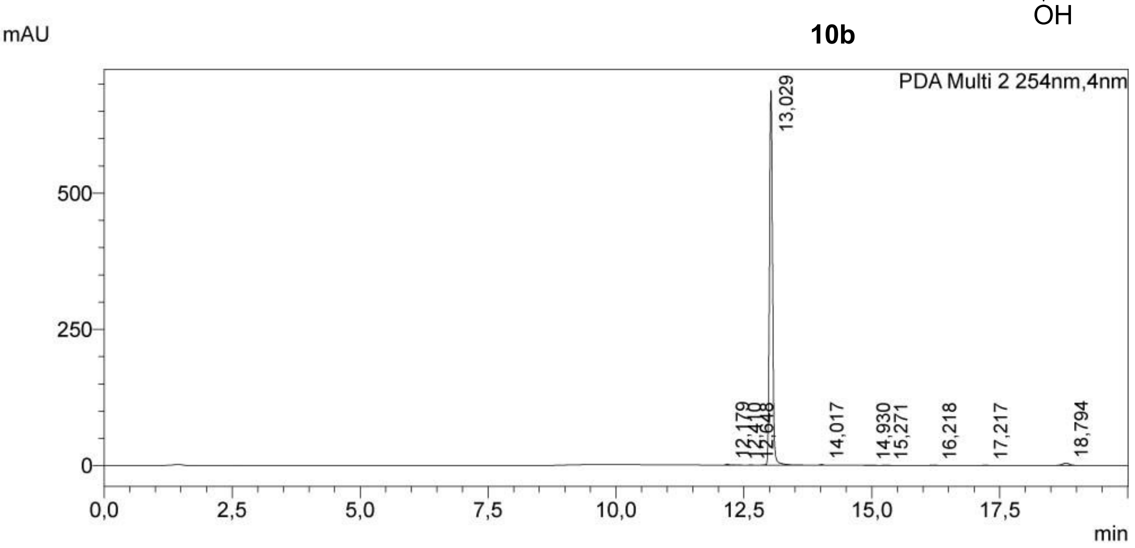

PDA Ch2 254nm

Peak#	Ret. Time	Area	Area%	Height
1	12,179	11249	0,387	1841
2	12,410	1580	0,054	285
3	12,648	2047	0,071	340
4	13,029	2832873	97,571	688005
5	14,017	6900	0,238	1546
6	14,930	2129	0,073	249
7	15,271	1509	0,052	197
8	16,218	2610	0,090	319
9	17,217	1679	0,058	172
10	18,794	40815	1,406	4173
Total		2903391	100,000	697127

Figure S6. HPLC chromatogram of compound **10b**.

Sample Name : MDS38_500uM
Sample ID : MDS38_500uM
Data Filename : MDS38_500uM_p.lcd
Method Filename : MAGL254.lcm

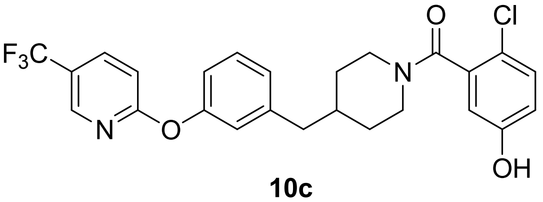

10c

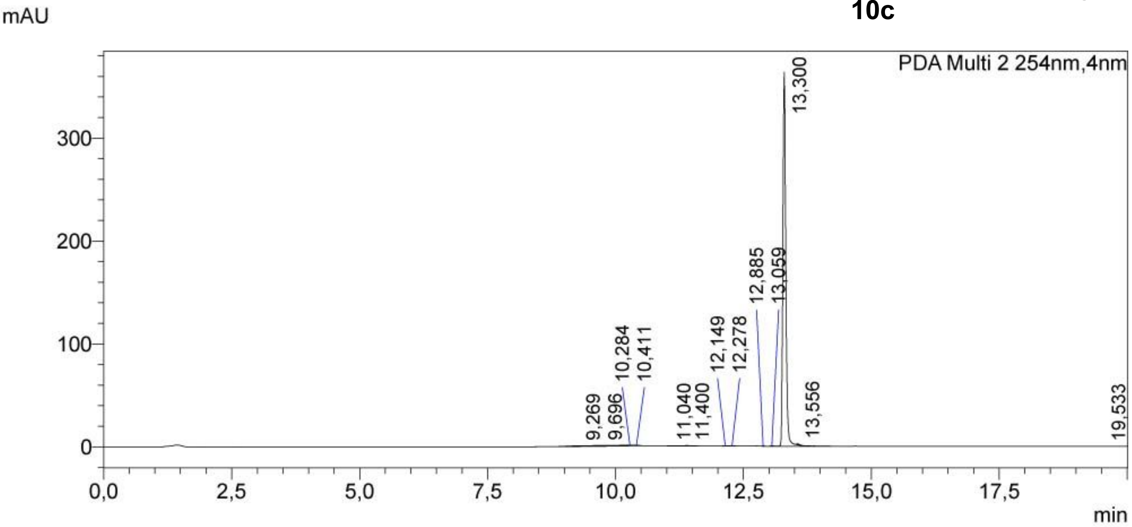

PDA Ch2 254nm

Peak#	Ret. Time	Area	Area%	Height
1	9,269	15456	1,032	565
2	9,696	16467	1,099	653
3	10,284	16772	1,119	709
4	10,411	5279	0,352	692
5	11,040	4052	0,270	172
6	11,400	4672	0,312	414
7	12,149	2369	0,158	383
8	12,278	3857	0,257	421
9	12,885	1178	0,079	352
10	13,059	2886	0,193	639
11	13,300	1421291	94,860	363576
12	13,556	2838	0,189	920
13	19,533	1194	0,080	115
Total		1498311	100,000	369612

Figure S7. HPLC chromatogram of compound **10c**.

Sample Name : MDS29_500uM
Sample ID : MDS29_500uM
Data Filename : MDS29_500uM_p.lcd
Method Filename : MAGL254.lcm

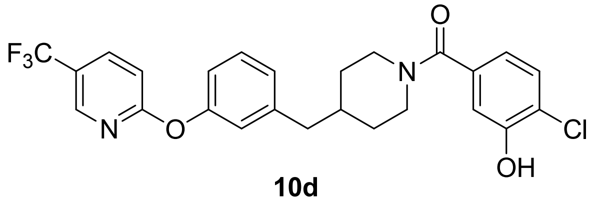

10d

mAU

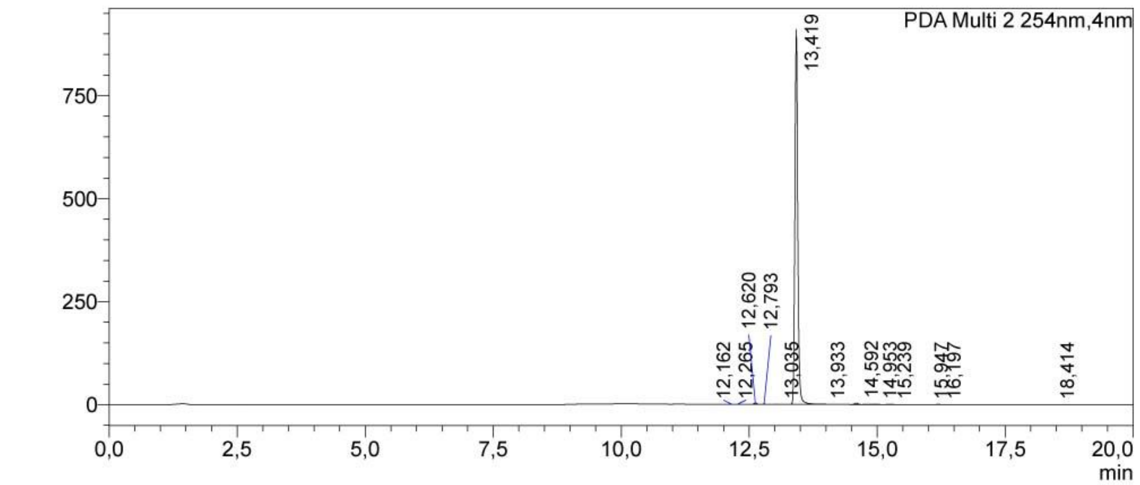

PDA Ch2 254nm

Peak#	Ret. Time	Area	Area%	Height
1	12,162	2235	0,060	337
2	12,265	2591	0,069	383
3	12,620	16410	0,439	3327
4	12,793	4732	0,127	869
5	13,035	3166	0,085	670
6	13,419	3668990	98,242	910441
7	13,933	1843	0,049	280
8	14,592	15363	0,411	3455
9	14,953	6579	0,176	772
10	15,239	3664	0,098	494
11	15,947	2044	0,055	191
12	16,197	2846	0,076	406
13	18,414	4186	0,112	367
Total		3734648	100,000	921993

Figure S8. HPLC chromatogram of compound **10d**.

Sample Name : MDS35_500uM
Sample ID : MDS35_500uM
Data Filename : MDS35_500uM_p.lcd
Method Filename : MAGL254.lcm

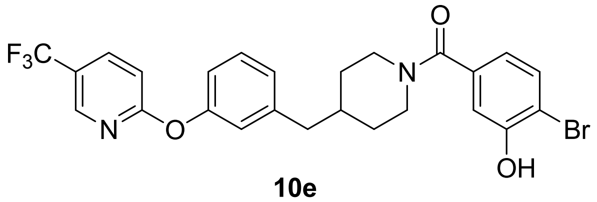

10e

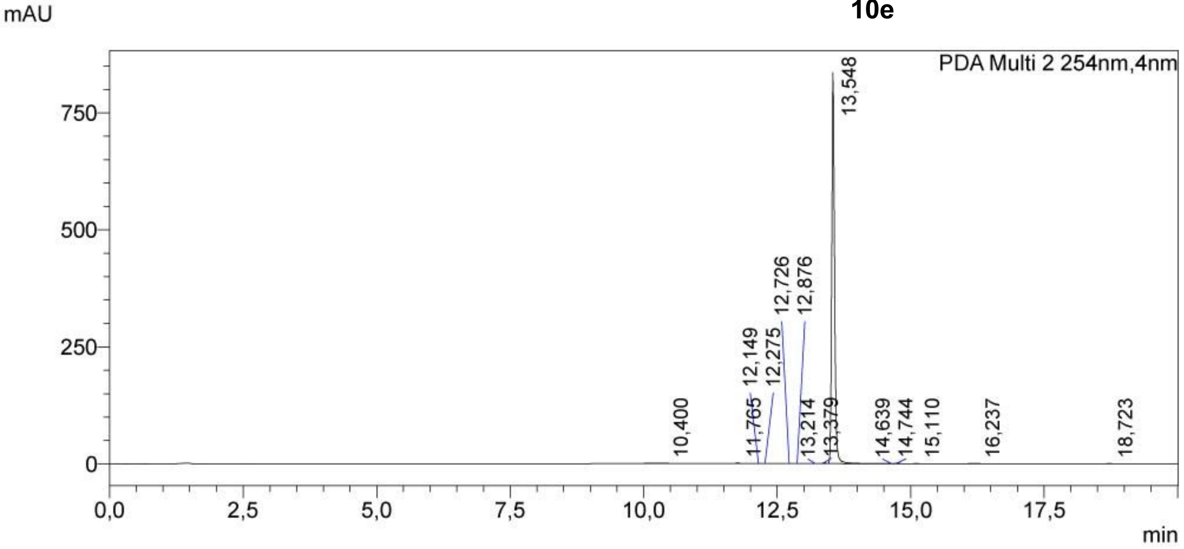

PDA Ch2 254nm

Peak#	Ret. Time	Area	Area%	Height
1	10,400	11139	0,342	670
2	11,765	5469	0,168	1154
3	12,149	1355	0,042	228
4	12,275	2748	0,084	428
5	12,726	1492	0,046	410
6	12,876	1859	0,057	539
7	13,214	1575	0,048	165
8	13,379	8332	0,256	2030
9	13,548	3205709	98,523	835537
10	14,639	1702	0,052	370
11	14,744	4659	0,143	1051
12	15,110	1446	0,044	188
13	16,237	3765	0,116	211
14	18,723	2511	0,077	247
Total		3253761	100,000	843227

Figure S9. HPLC chromatogram of compound **10e**.

Sample Name : FS10_500uM
Sample ID : FS10_500uM
Data Filename : FS10_500uM_p.lcd
Method Filename : MAGL254.lcm

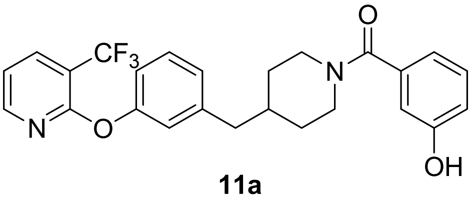

11a

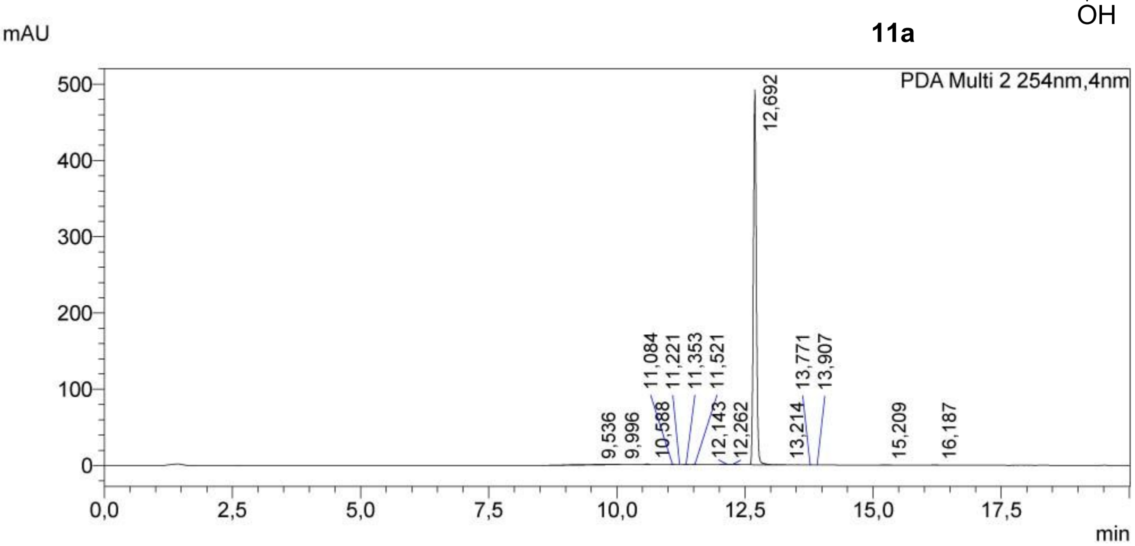

PDA Ch2 254nm

Peak#	Ret. Time	Area	Area%	Height
1	9,536	21660	1,093	415
2	9,996	2758	0,139	242
3	10,588	2270	0,115	585
4	11,084	1877	0,095	362
5	11,221	1048	0,053	164
6	11,353	1946	0,098	250
7	11,521	1864	0,094	259
8	12,143	7222	0,365	720
9	12,262	6201	0,313	676
10	12,692	1918343	96,844	491857
11	13,214	1634	0,082	270
12	13,771	1270	0,064	334
13	13,907	3064	0,155	197
14	15,209	4734	0,239	492
15	16,187	4960	0,250	565
Total		1980851	100,000	497386

Figure S10. HPLC chromatogram of compound **11a**.

Sample Name : FS5_500uM
Sample ID : FS5_500uM
Data Filename : FS5_500uM_p.lcd
Method Filename : MAGL254.lcm

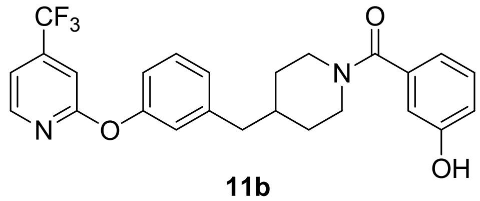

11b

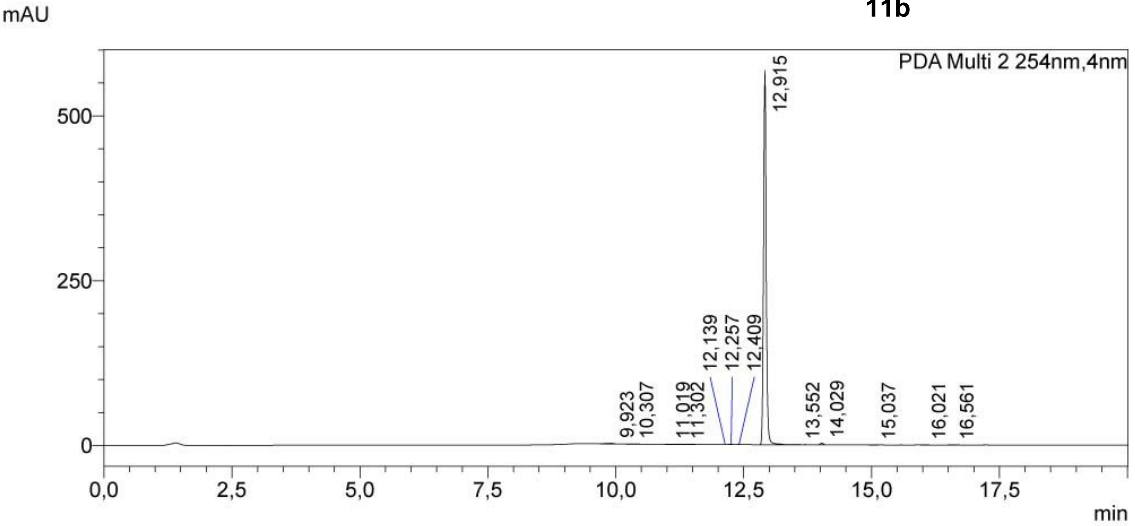

PDA Ch2 254nm

Peak#	Ret. Time	Area	Area%	Height
1	9,923	9548	0,435	973
2	10,307	3810	0,174	275
3	11,019	3357	0,153	246
4	11,302	6285	0,287	415
5	12,139	1646	0,075	317
6	12,257	4273	0,195	719
7	12,409	2511	0,114	512
8	12,915	2144145	97,761	567851
9	13,552	1973	0,090	415
10	14,029	12088	0,551	2854
11	15,037	1436	0,065	225
12	16,021	1098	0,050	189
13	16,561	1093	0,050	185
Total		2193262	100,000	575176

Figure S11. HPLC chromatogram of compound **11b**.

Sample Name : CG1645B_500uM
Sample ID : CG1645B_500uM
Data Filename : CG1645B_500uM_p.lcd
Method Filename : MAGL254.lcm

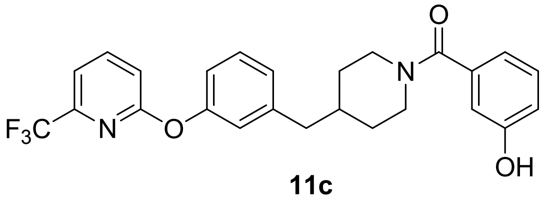

11c

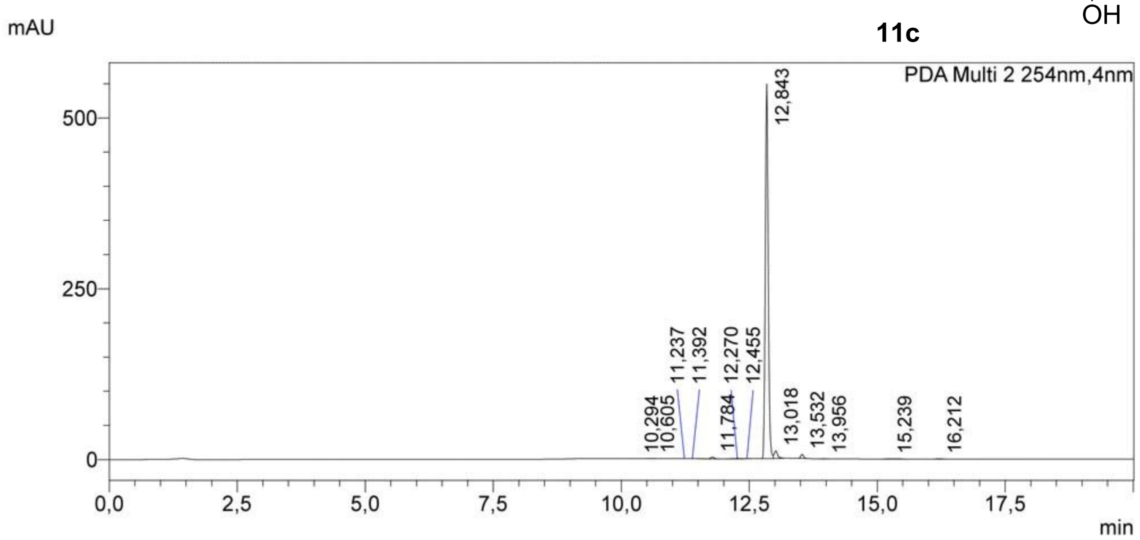

PDA Ch2 254nm

Peak#	Ret. Time	Area	Area%	Height
1	10,294	2868	0,131	247
2	10,605	1050	0,048	298
3	11,237	2934	0,134	224
4	11,392	3006	0,137	239
5	11,784	10877	0,495	2505
6	12,270	4369	0,199	530
7	12,455	2318	0,106	385
8	12,843	2091534	95,249	548420
9	13,018	48681	2,217	10891
10	13,532	22376	1,019	5765
11	13,956	1605	0,073	274
12	15,239	2721	0,124	295
13	16,212	1514	0,069	245
Total		2195853	100,000	570318

Figure S12. HPLC chromatogram of compound **11c**.

Sample Name : GB445B_500uM
Sample ID : GB445B_500uM
Data Filename : GB445B_500uM_p.lcd
Method Filename : MAGL254.lcm

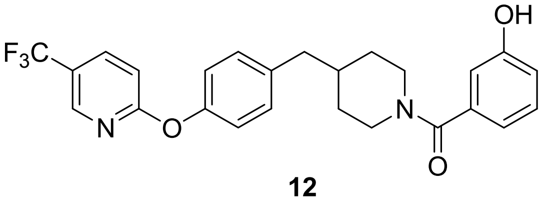

12

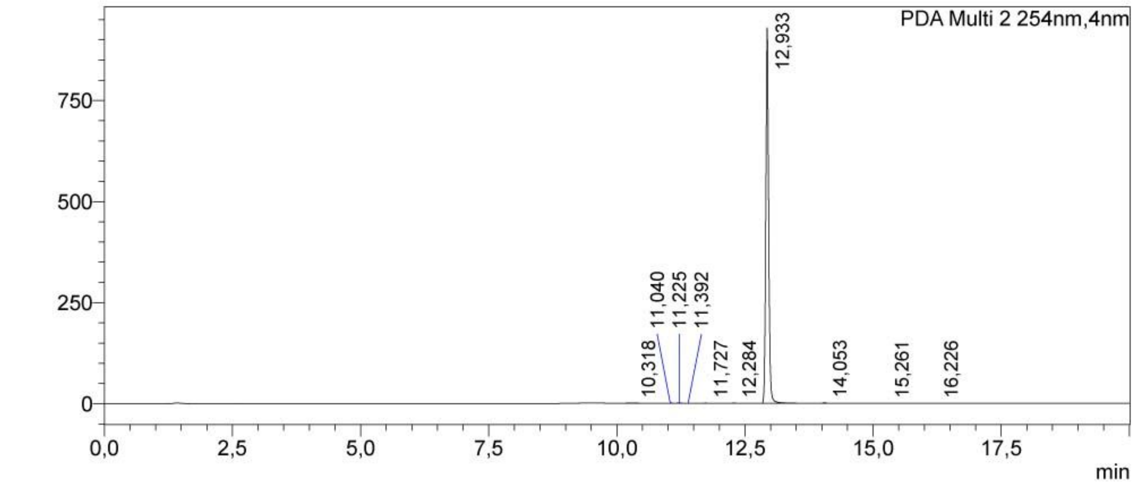

PDA Ch2 254nm

Peak#	Ret. Time	Area	Area%	Height
1	10,318	2935	0,078	218
2	11,040	1178	0,031	109
3	11,225	1441	0,038	213
4	11,392	2019	0,054	157
5	11,727	1646	0,044	393
6	12,284	3022	0,081	393
7	12,933	3726900	99,380	928754
8	14,053	7504	0,200	1469
9	15,261	2123	0,057	288
10	16,226	1369	0,036	227
Total		3750136	100,000	932220

Figure S13. HPLC chromatogram of compound **12**.

Sample Name : GB454_500uM
Sample ID : GB454_500uM
Data Filename : GB454_500uM_p.lcd
Method Filename : MAGL254.lcm

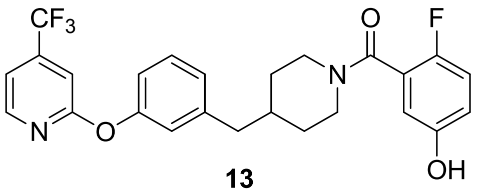

13

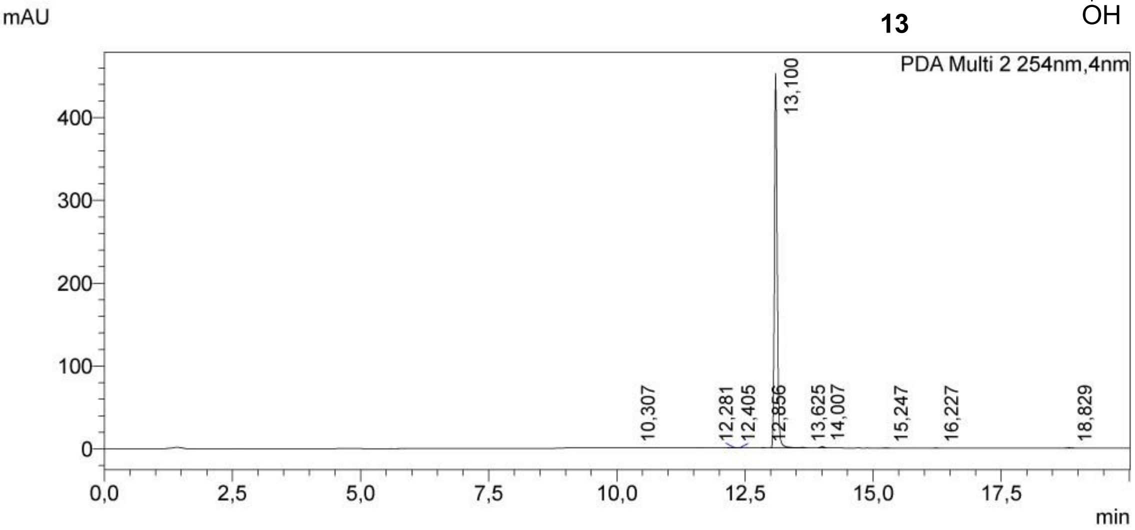

PDA Ch2 254nm

Peak#	Ret. Time	Area	Area%	Height
1	10,307	2382	0,132	177
2	12,281	4953	0,274	538
3	12,405	1104	0,061	227
4	12,856	1469	0,081	388
5	13,100	1771779	98,189	452536
6	13,625	2146	0,119	516
7	14,007	10524	0,583	1842
8	15,247	3076	0,170	330
9	16,227	1712	0,095	266
10	18,829	5314	0,294	548
Total		1804458	100,000	457369

Figure S14. HPLC chromatogram of compound **13**.

Sample Name : GB456_500uM
Sample ID : GB456_500uM
Data Filename : GB456_500uM_p.lcd
Method Filename : MAGL254.lcm

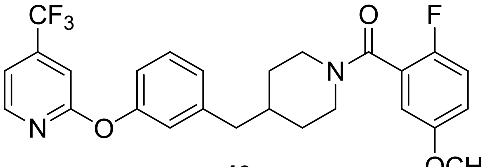

40

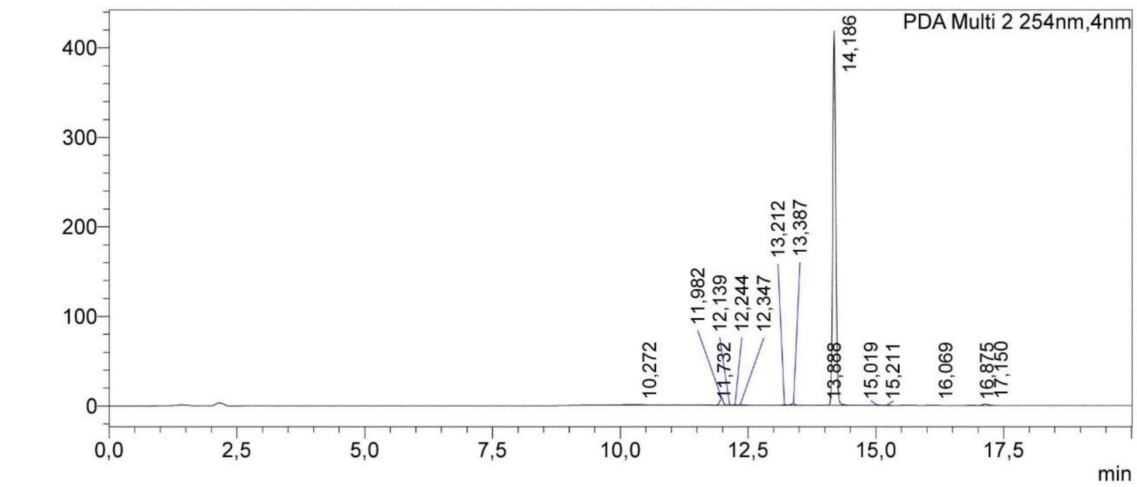

PDA Ch2 254nm

Peak#	Ret. Time	Area	Area%	Height
1	10,272	9284	0,505	425
2	11,732	1385	0,075	325
3	11,982	37747	2,055	8415
4	12,139	1590	0,087	314
5	12,244	2664	0,145	438
6	12,347	3301	0,180	393
7	13,212	3492	0,190	809
8	13,387	10638	0,579	2205
9	13,888	1234	0,067	143
10	14,186	1746830	95,098	418256
11	15,019	4220	0,230	488
12	15,211	1097	0,060	214
13	16,069	1166	0,063	213
14	16,875	1225	0,067	155
15	17,150	10996	0,599	1491
Total		1836868	100,000	434284

Figure S15. HPLC chromatogram of compound **40**.

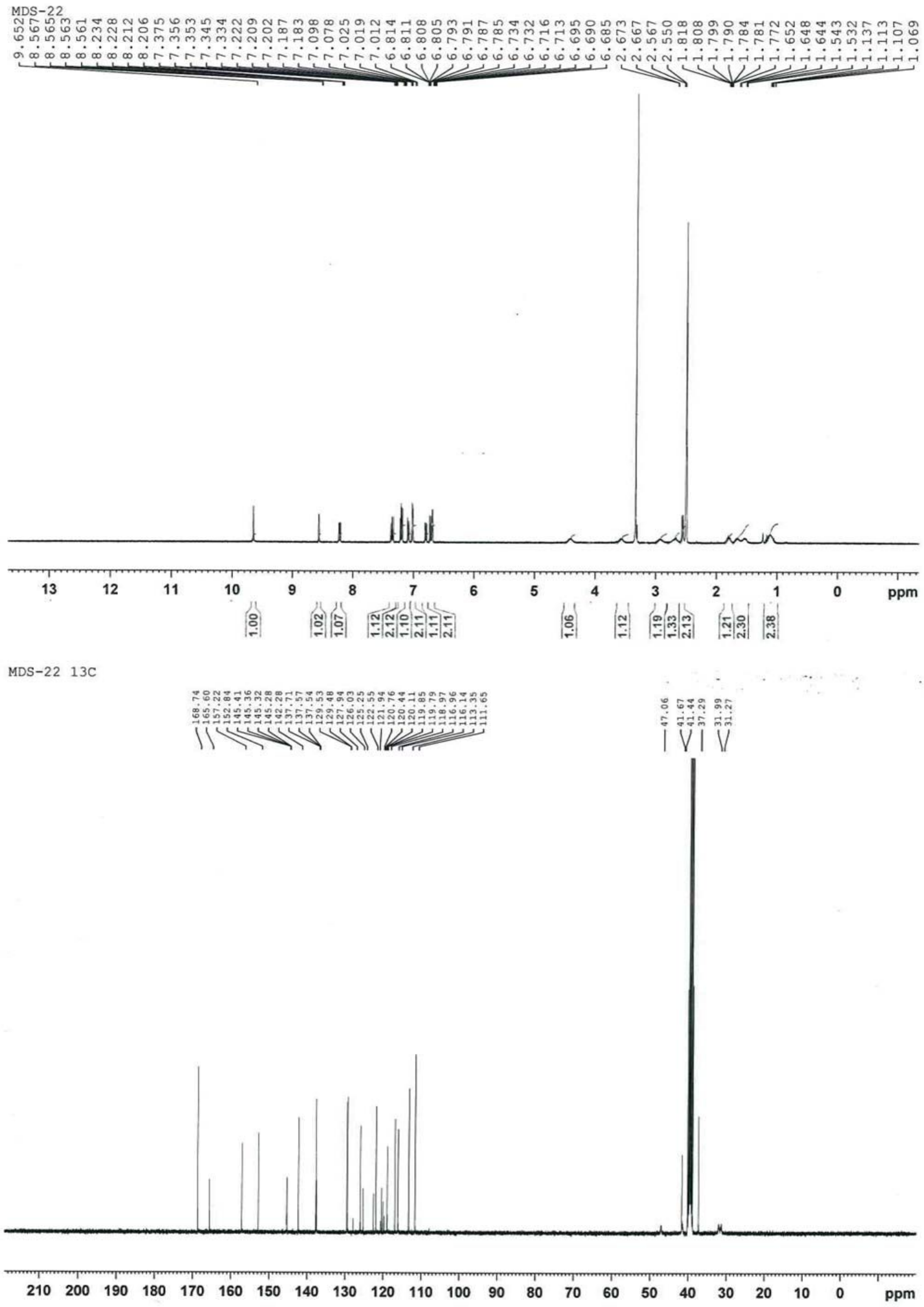

Figure S16. ^{1}H-NMR and ^{13}C-NMR of compound **7**.

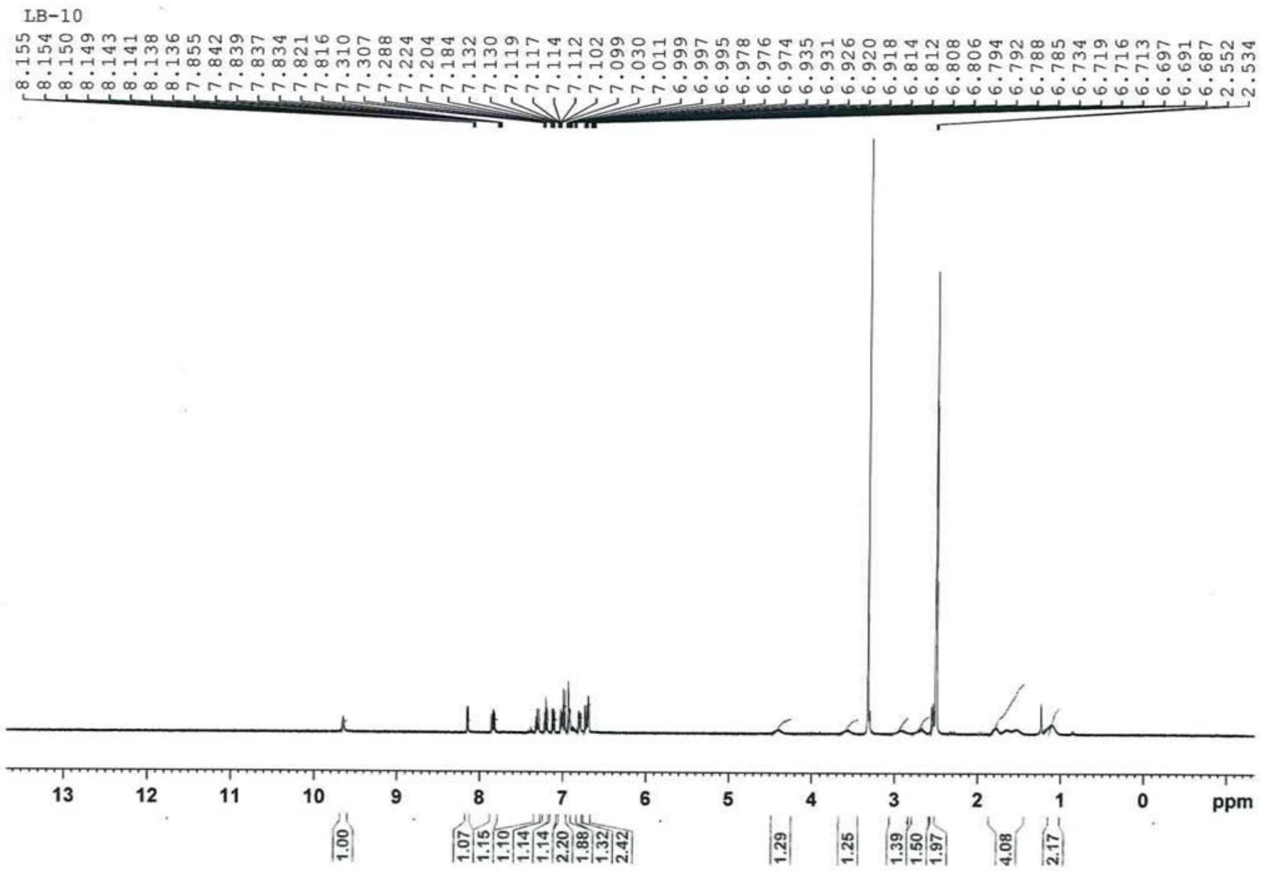

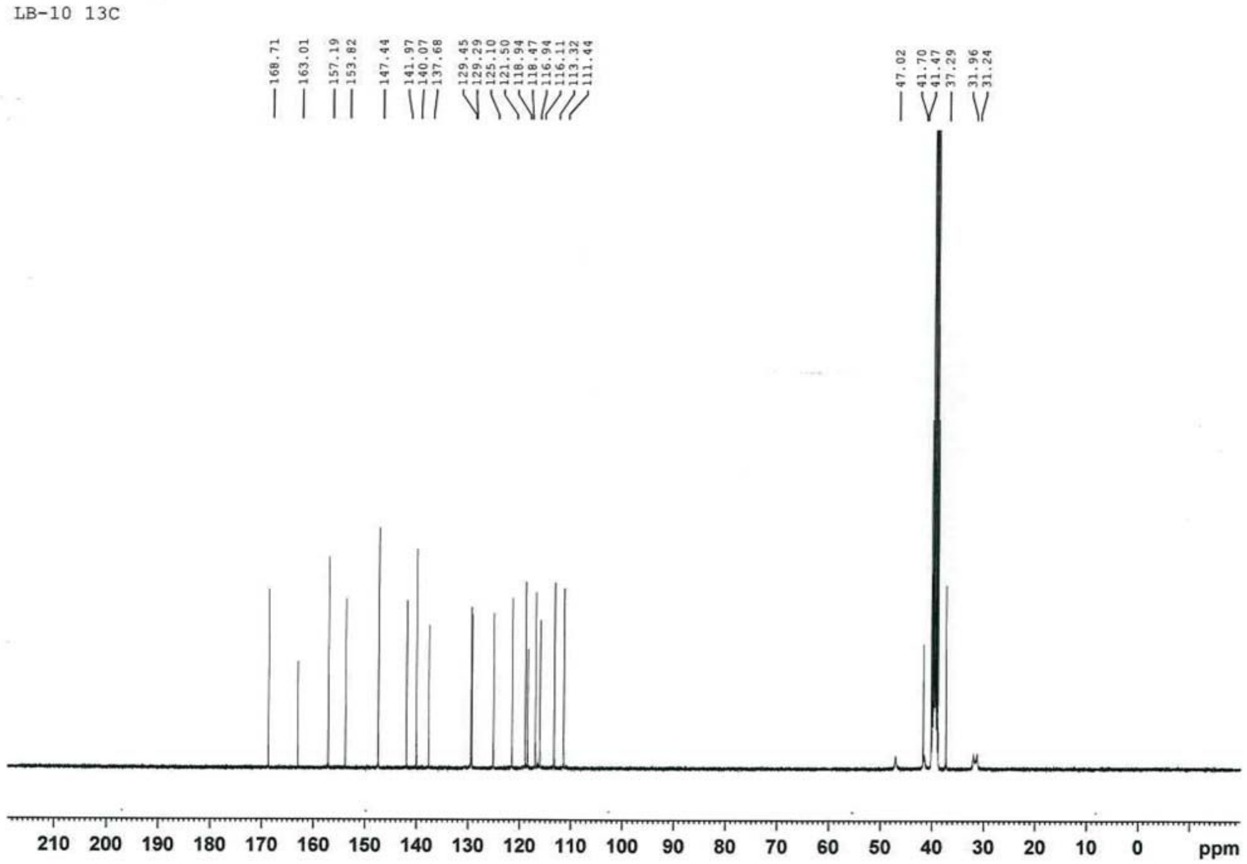

Figure S17. ^{1}H-NMR and ^{13}C-NMR of compound **8**.

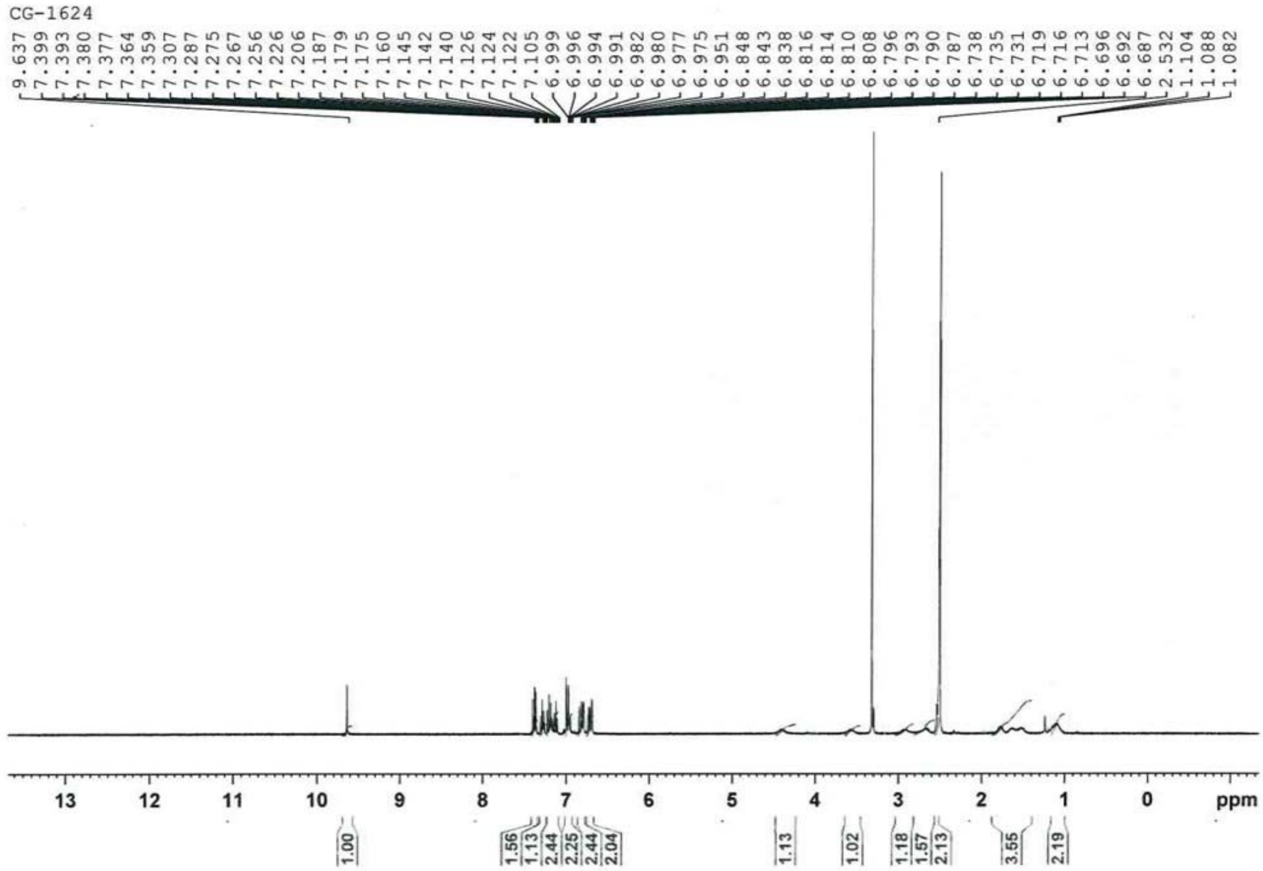

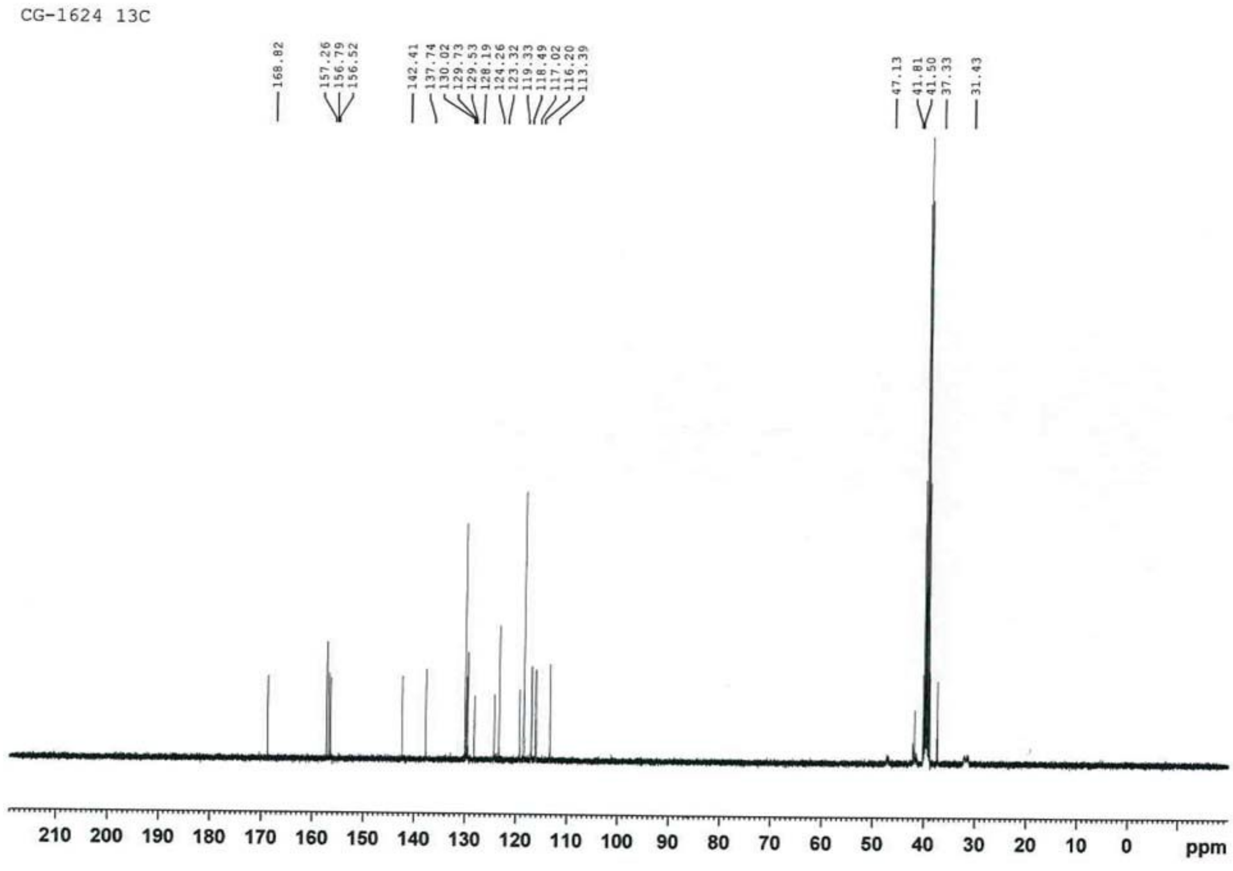

Figure S18. ^{1}H-NMR and ^{13}C-NMR of compound **9**.

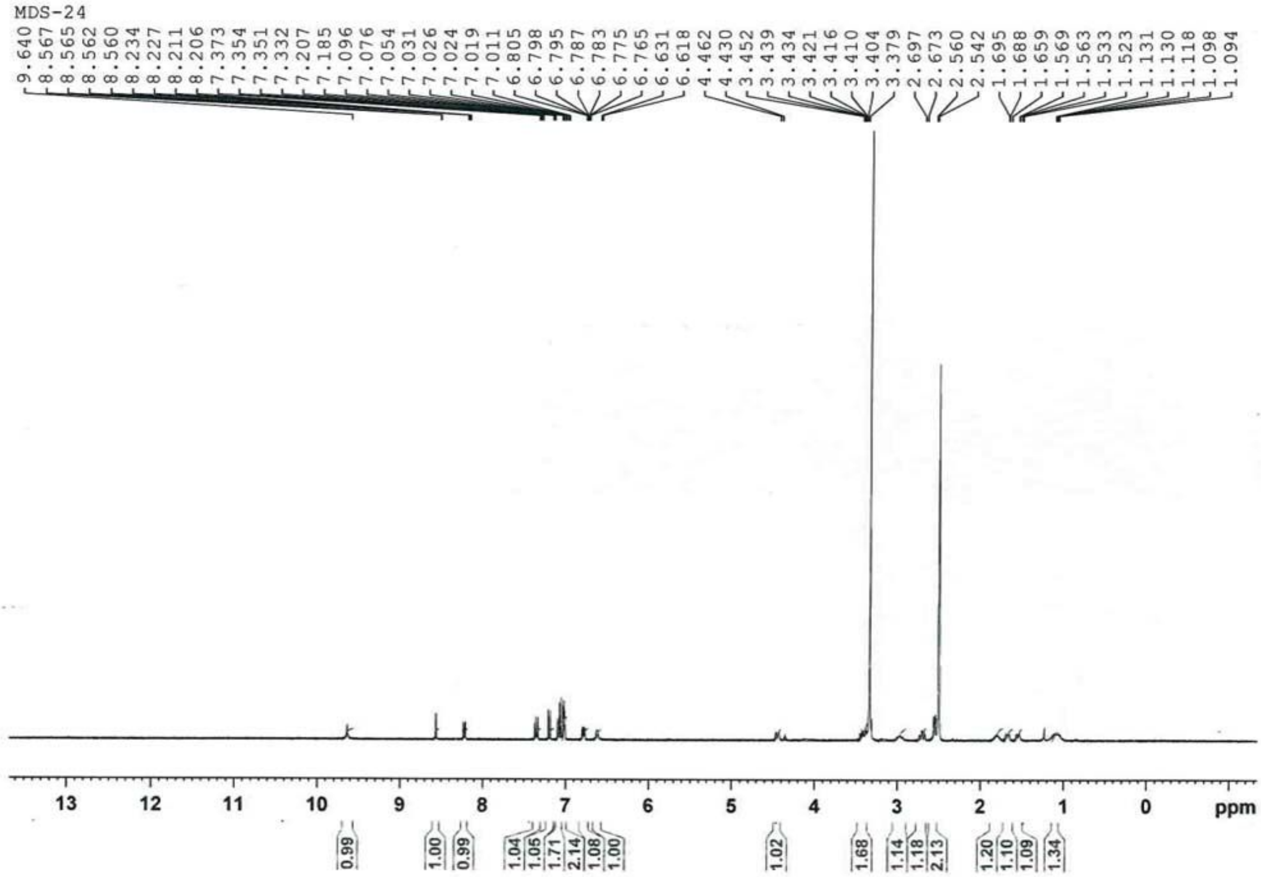

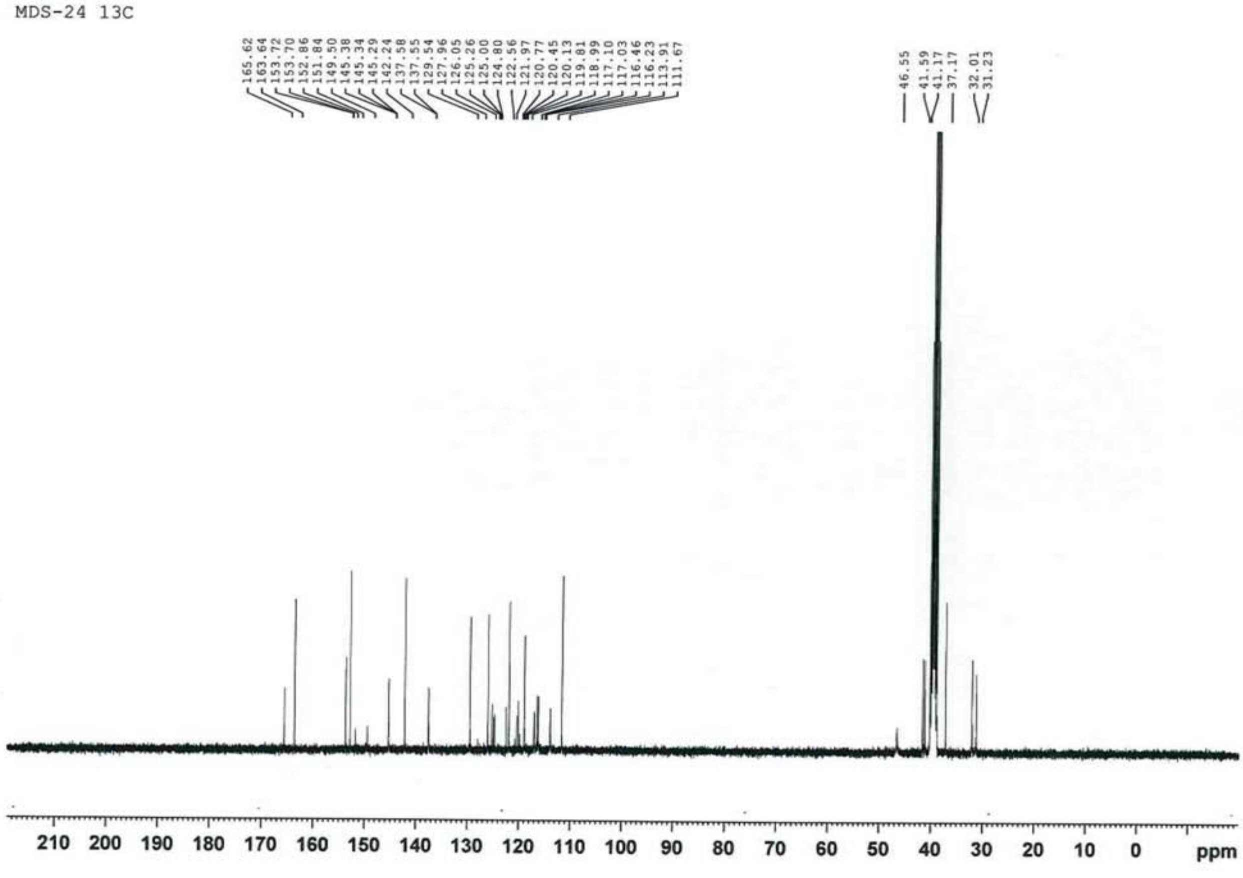

Figure S19. ^{1}H-NMR and ^{13}C-NMR of compound **10a**.

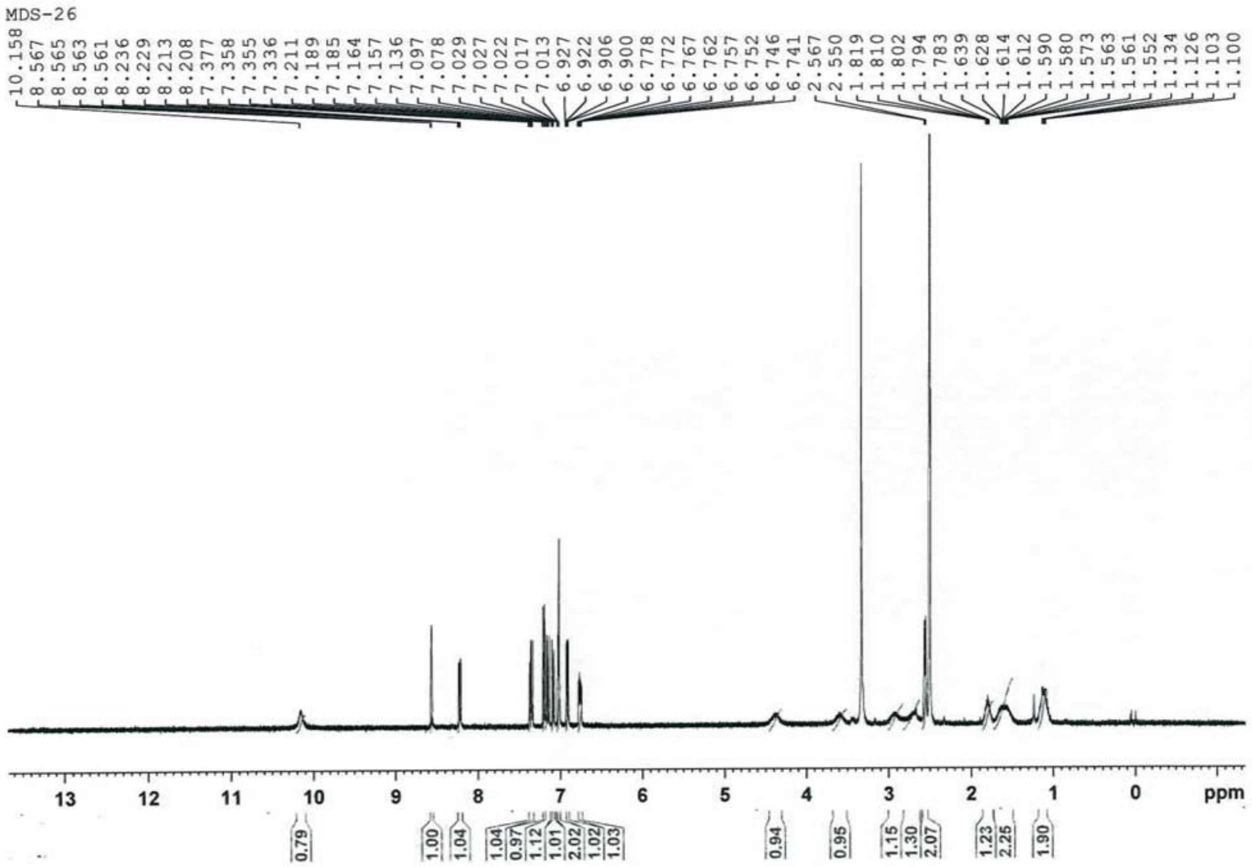

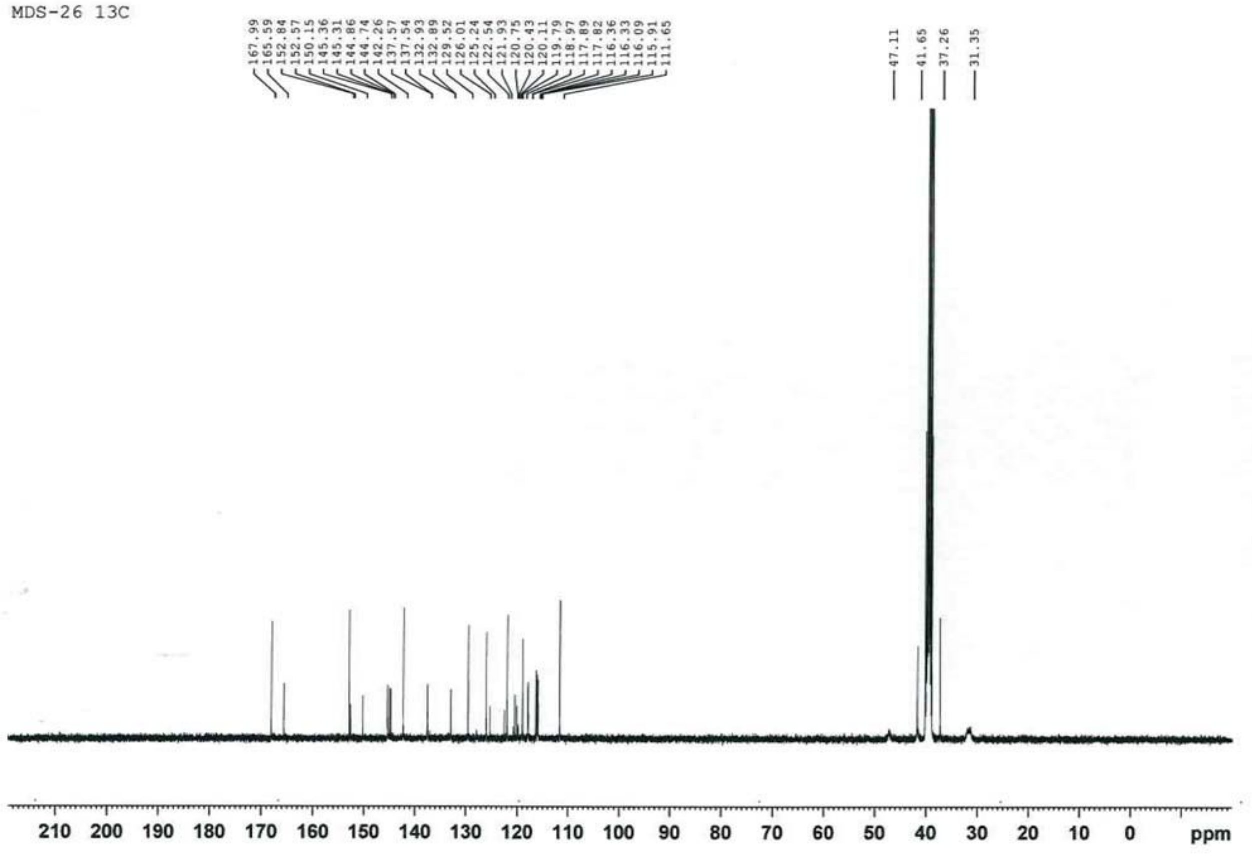

Figure S20. ^{1}H-NMR and ^{13}C-NMR of compound **10b**.

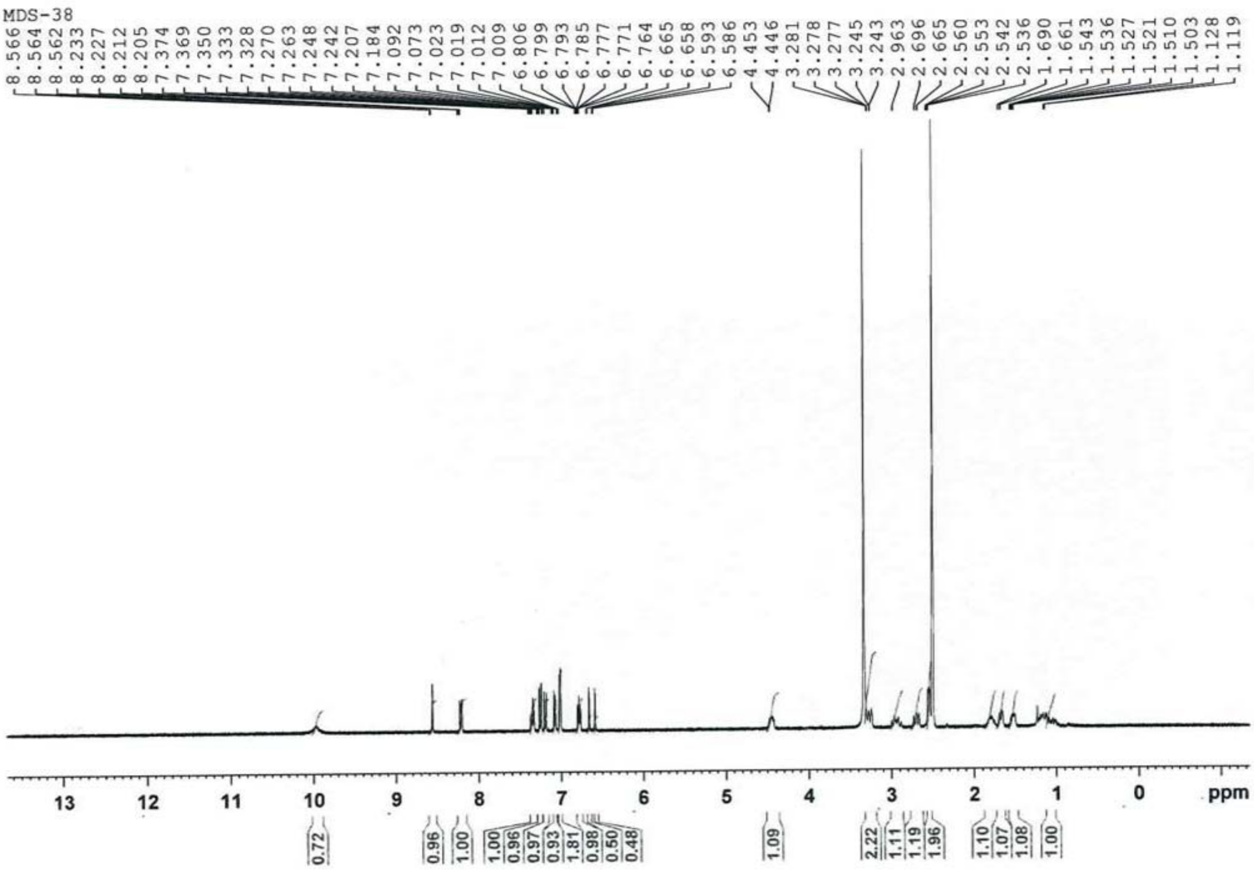

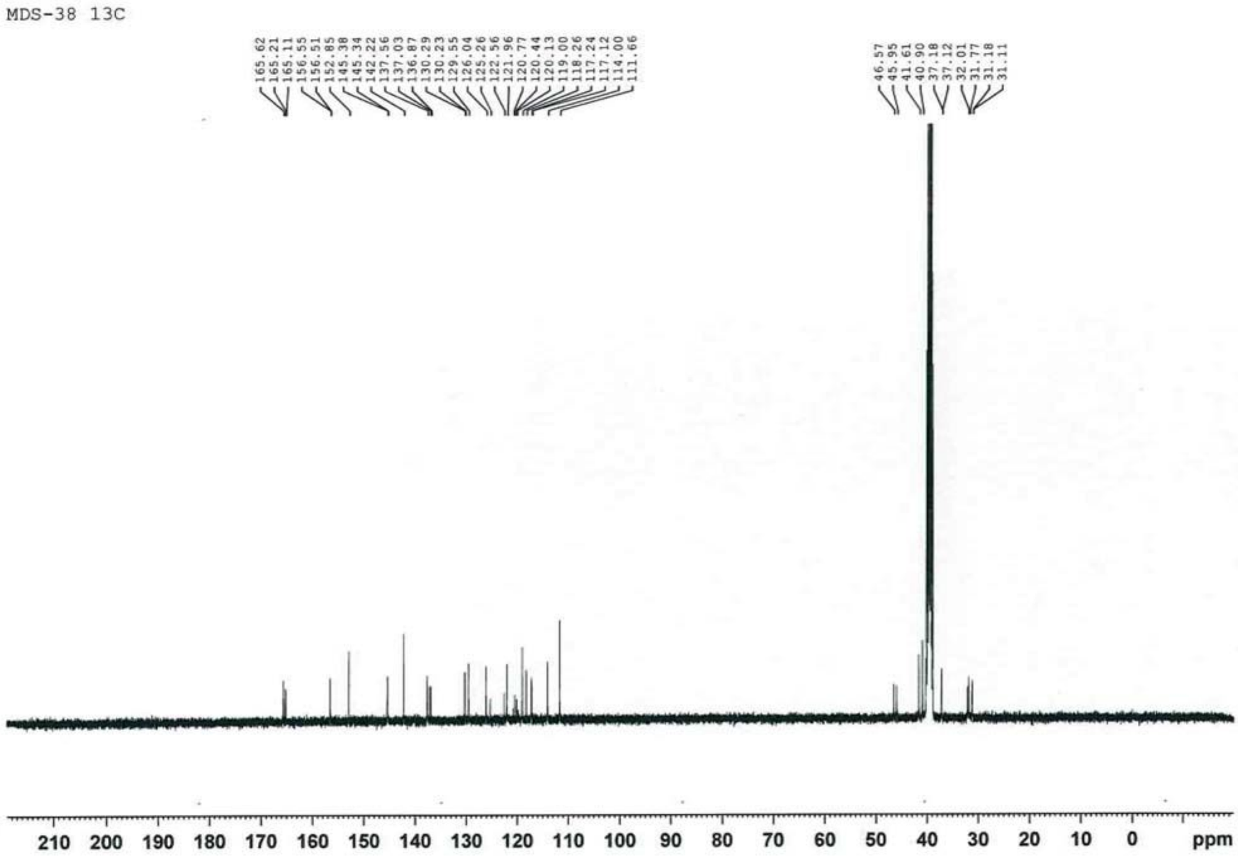

Figure S21. ^{1}H-NMR and ^{13}C-NMR of compound **10c**.

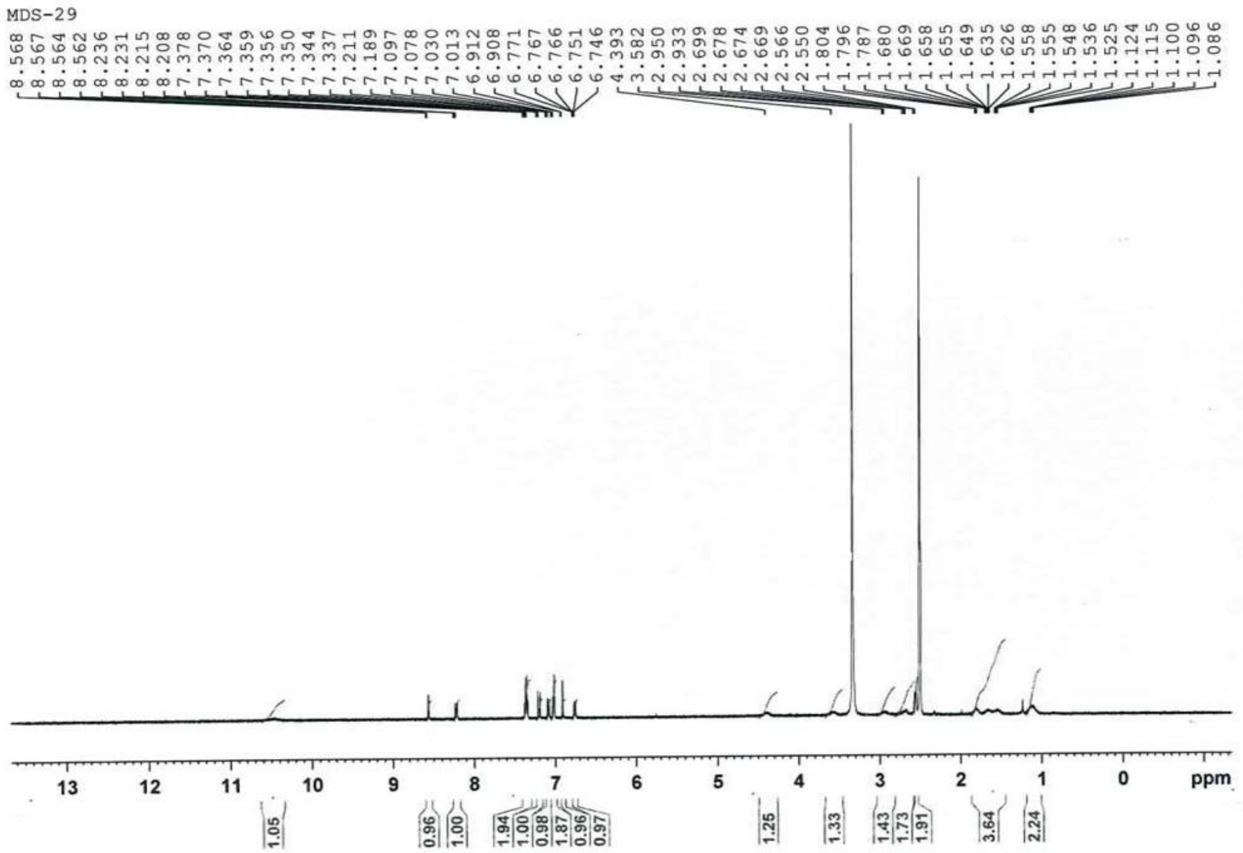

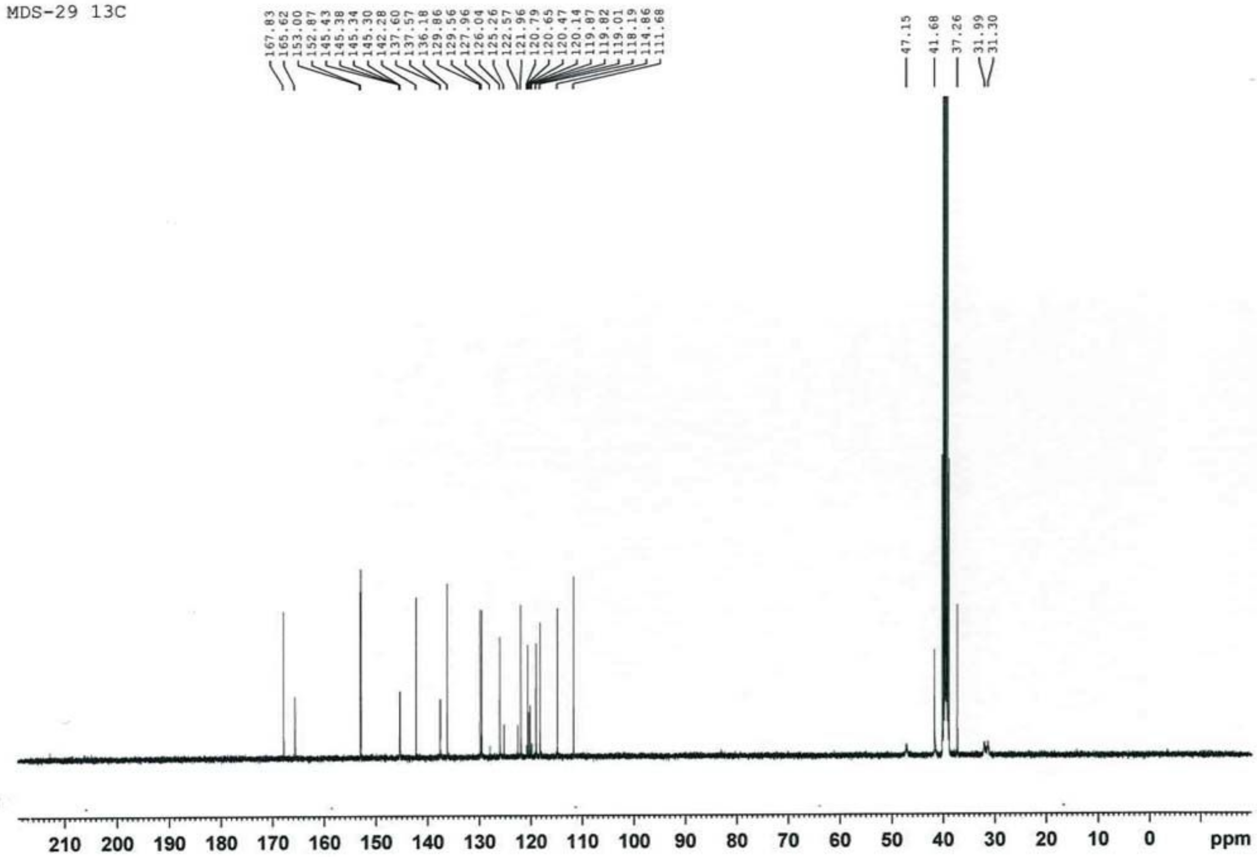

Figure S22. ^{1}H-NMR and ^{13}C-NMR of compound **10d**.

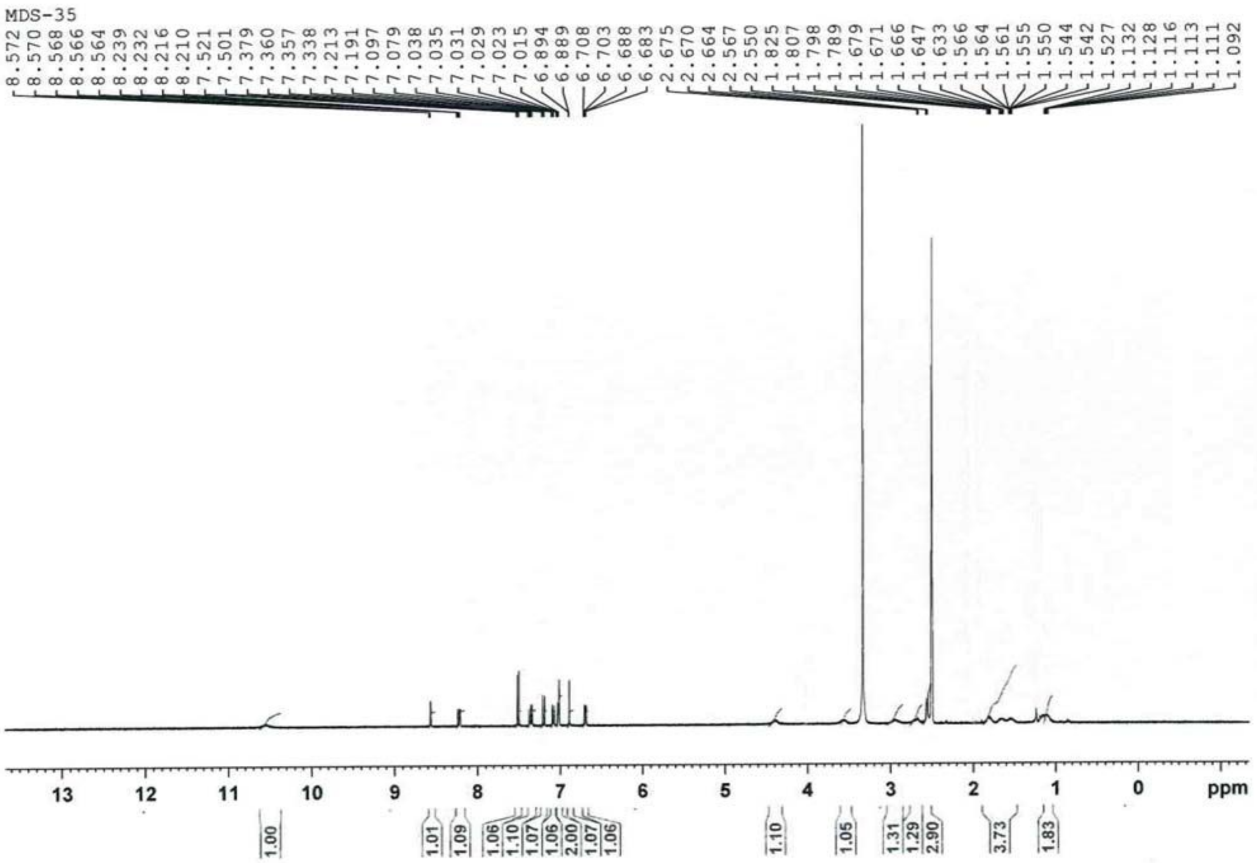

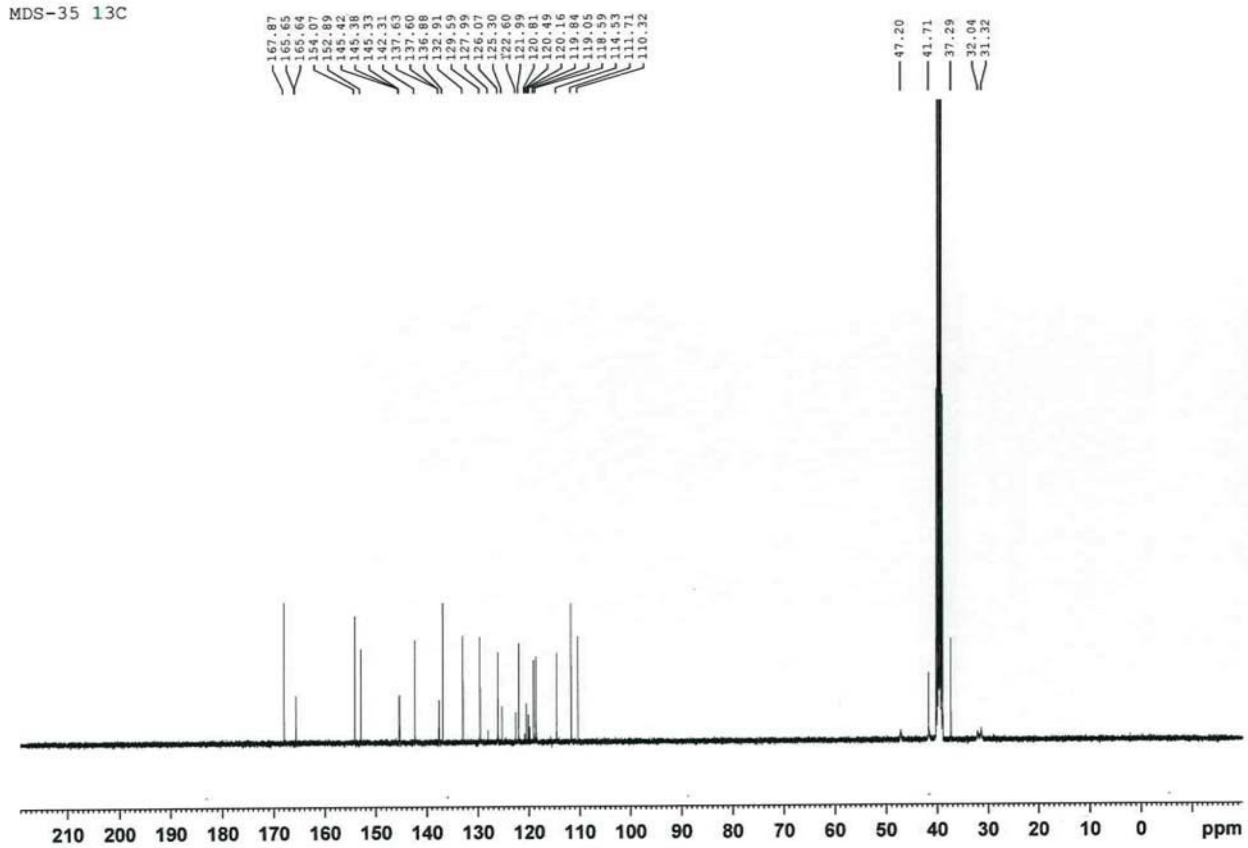

Figure S23. ^{1}H-NMR and ^{13}C-NMR of compound **10e**.

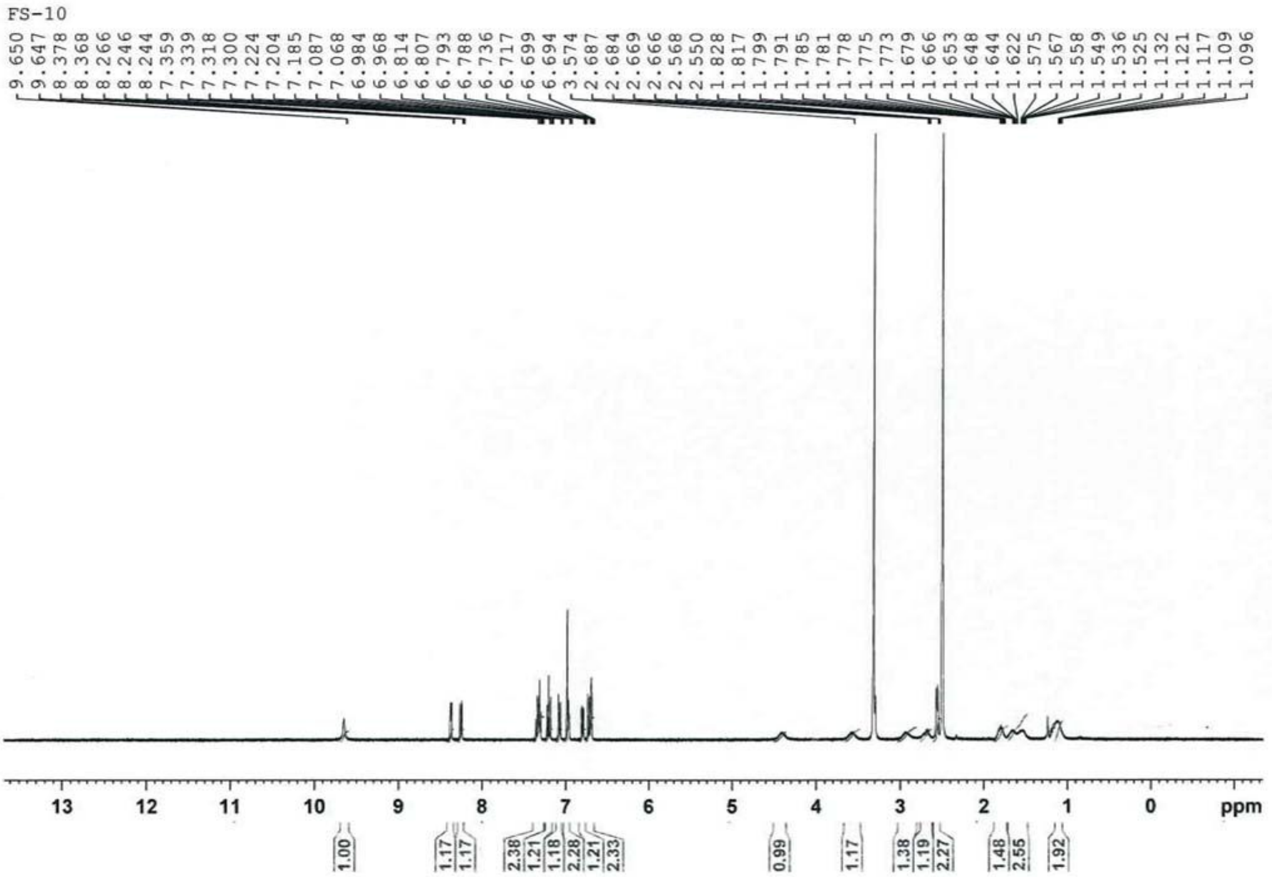

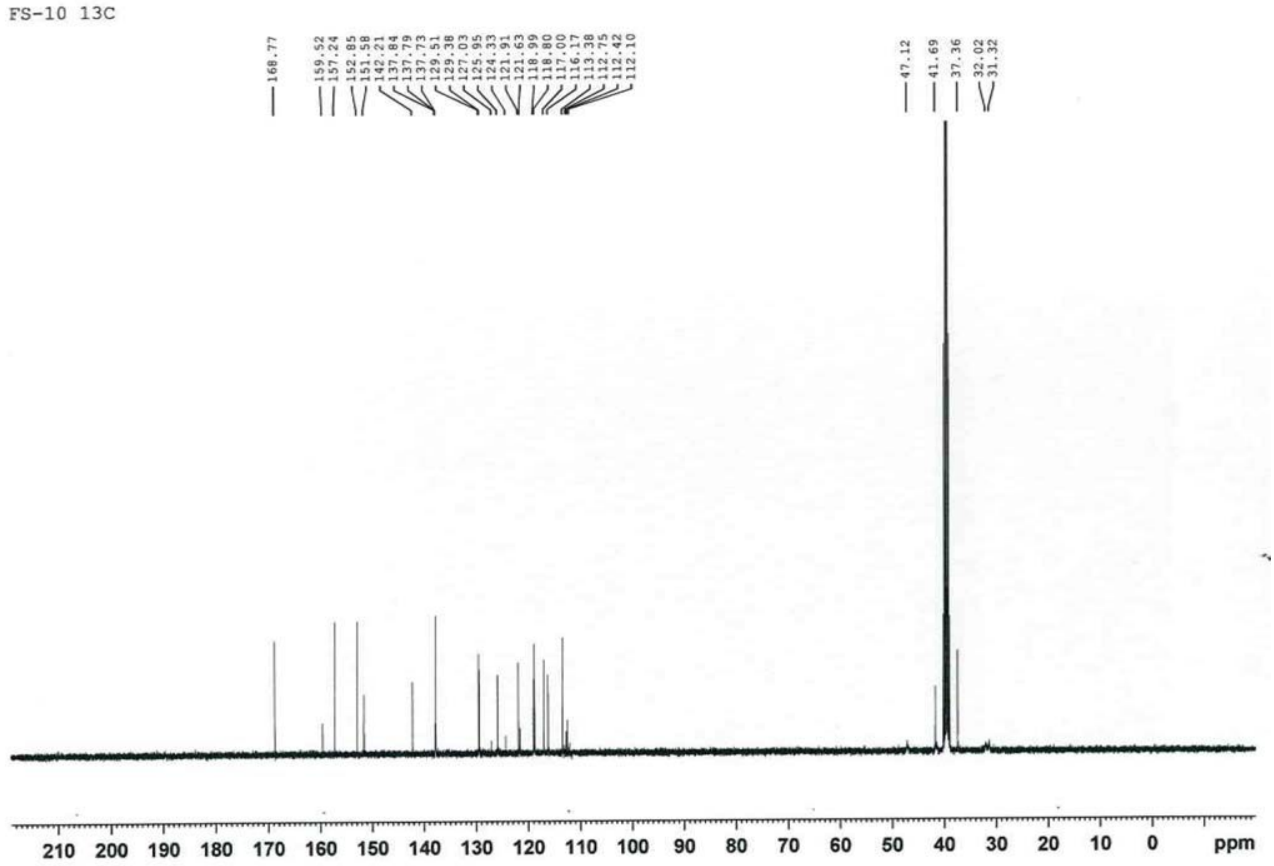

Figure S24. ^{1}H-NMR and ^{13}C-NMR of compound **11a**.

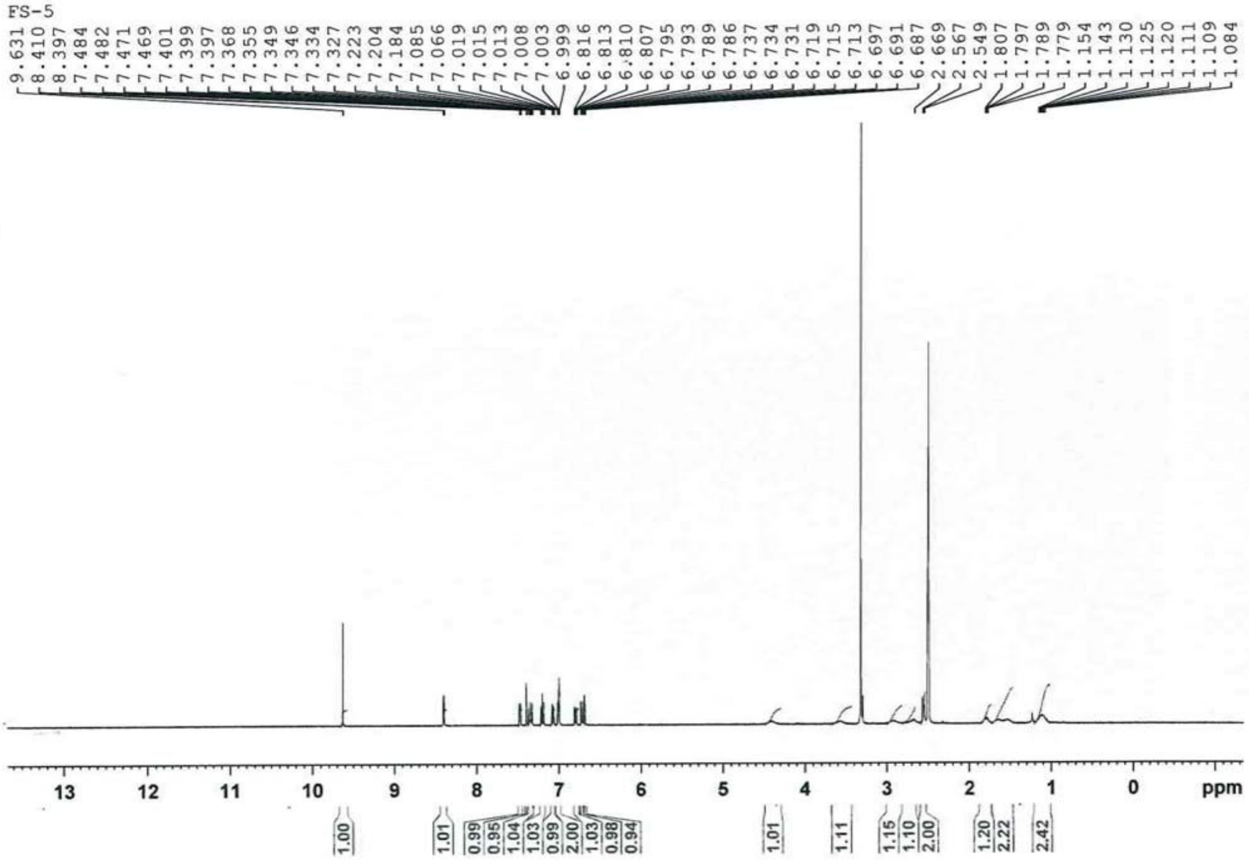

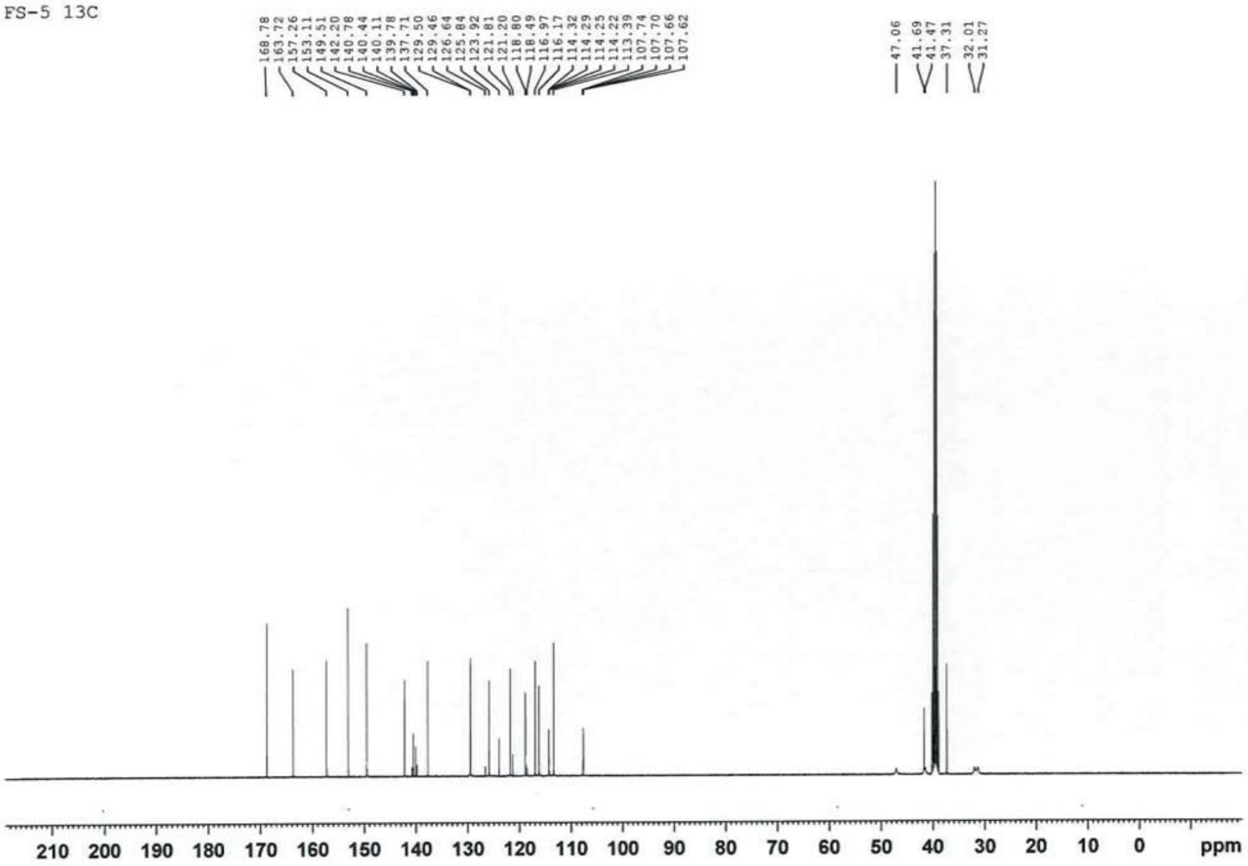

Figure S25. ^{1}H-NMR and ^{13}C-NMR of compound **11b**.

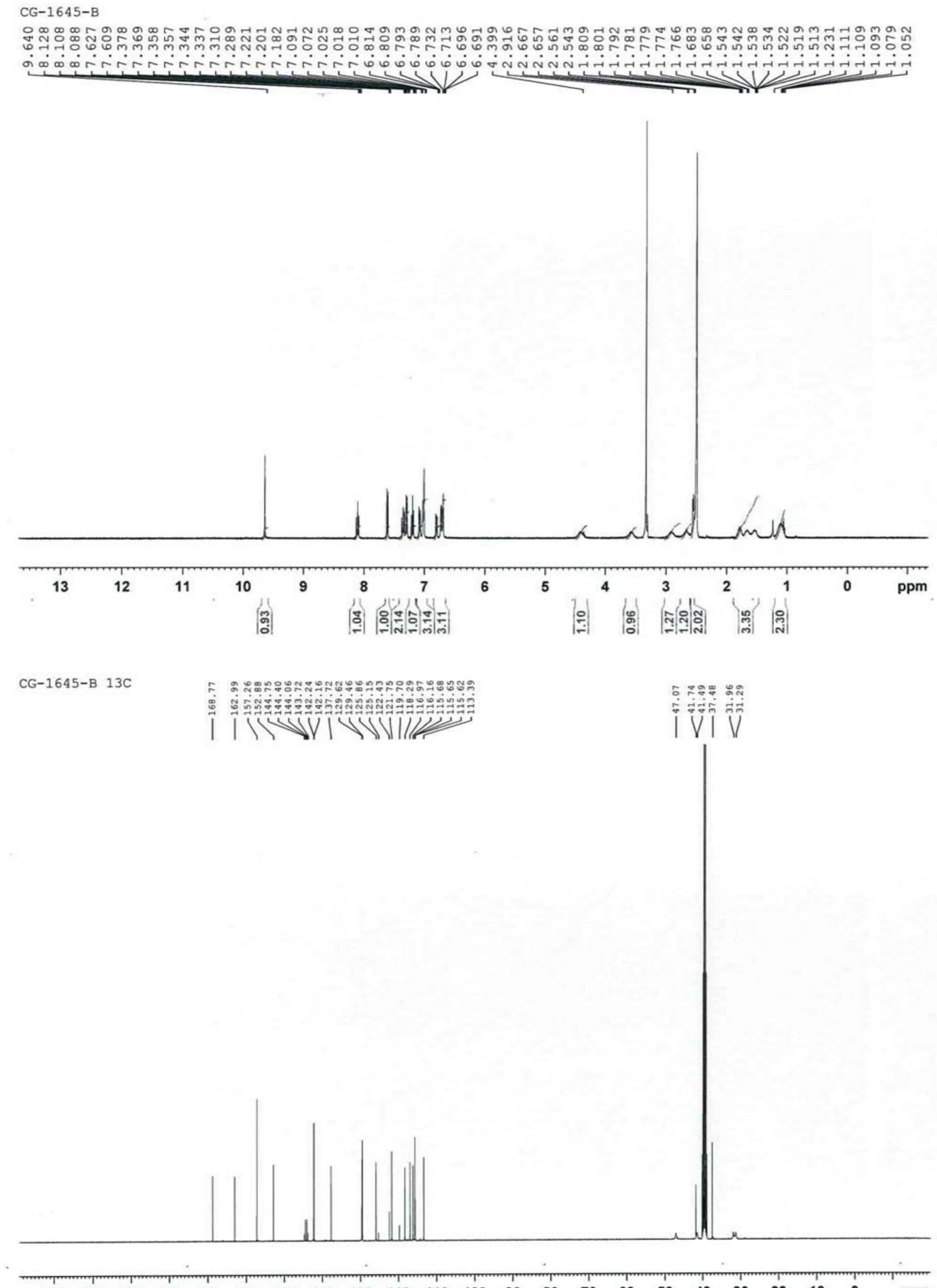

Figure S26. ^{1}H-NMR and ^{13}C-NMR of compound **11c**.

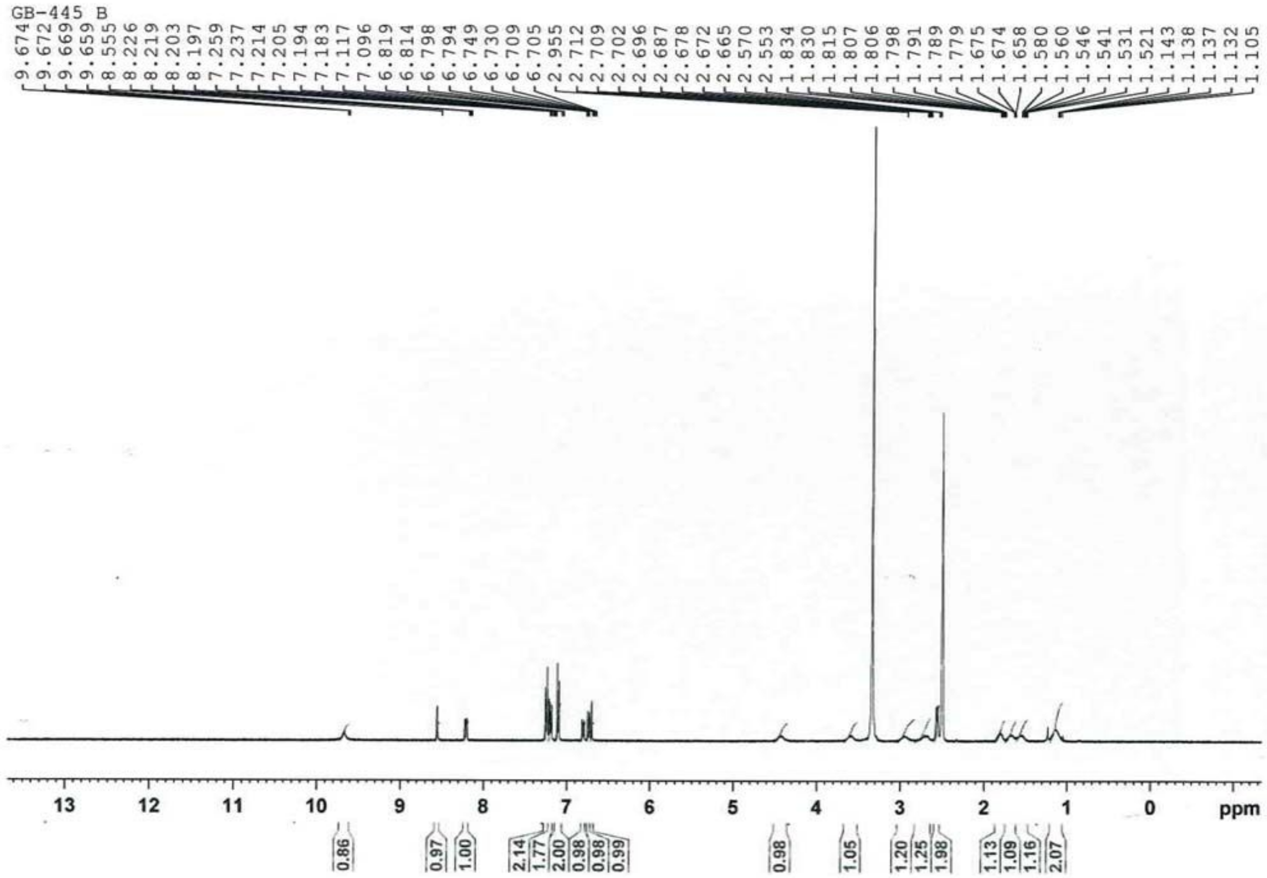

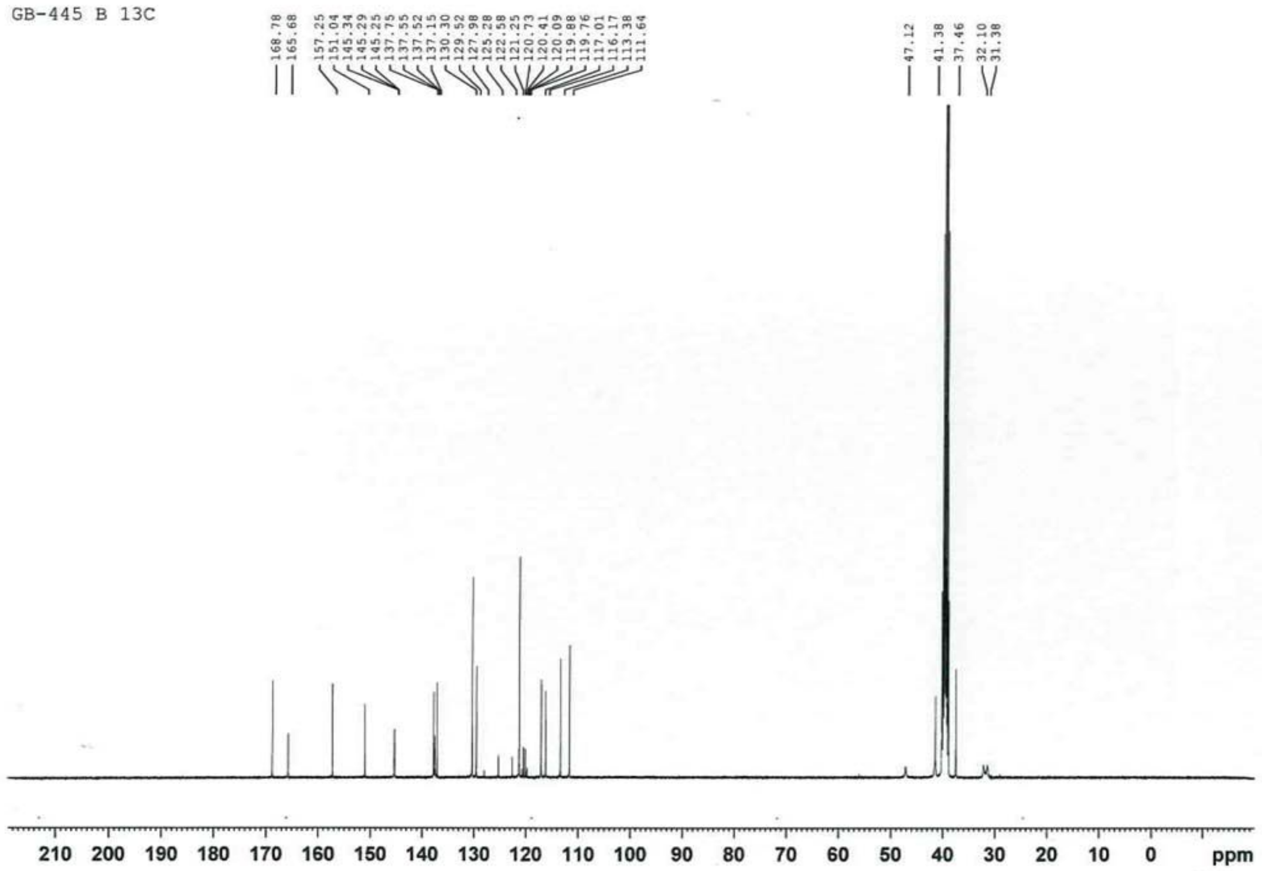

Figure S27. ^{1}H-NMR and ^{13}C-NMR of compound **12**.

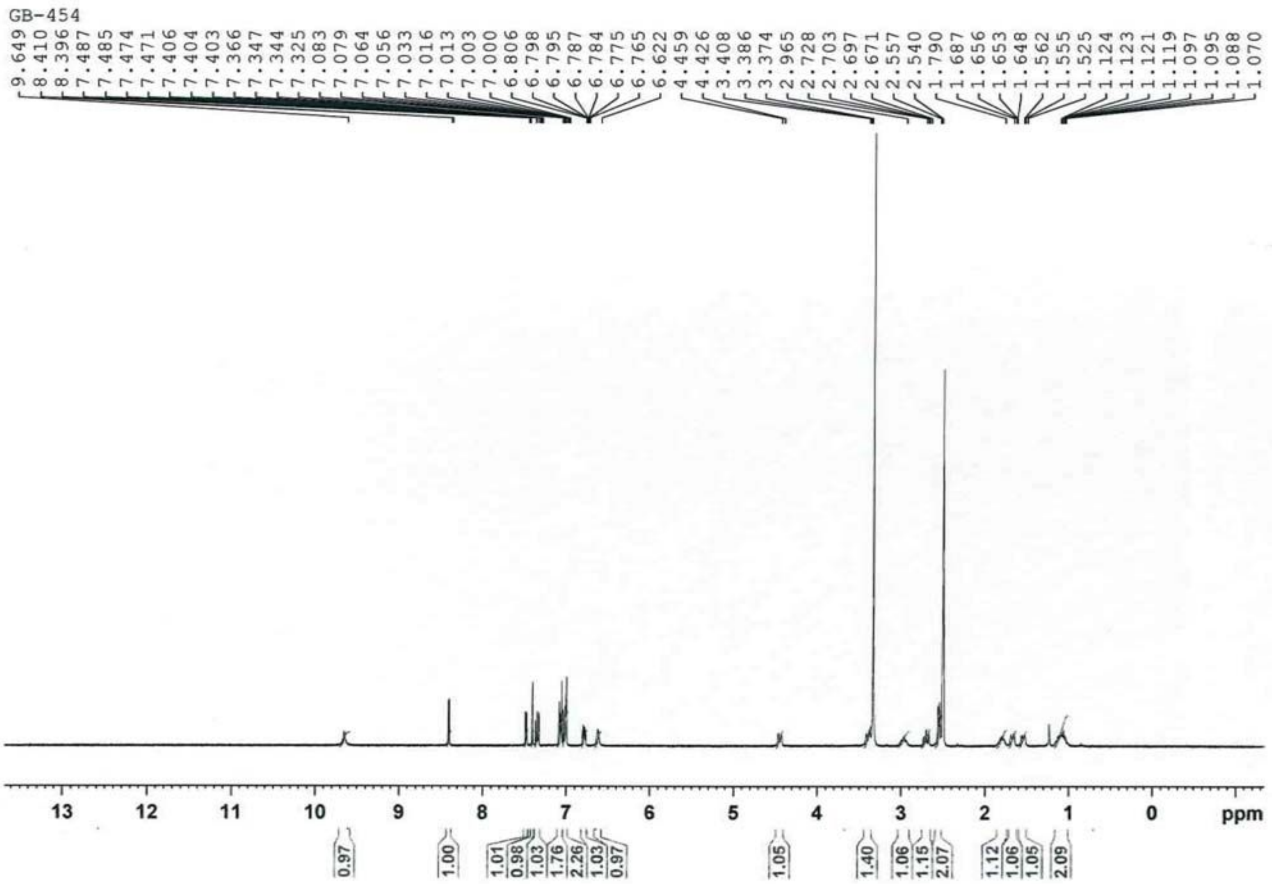

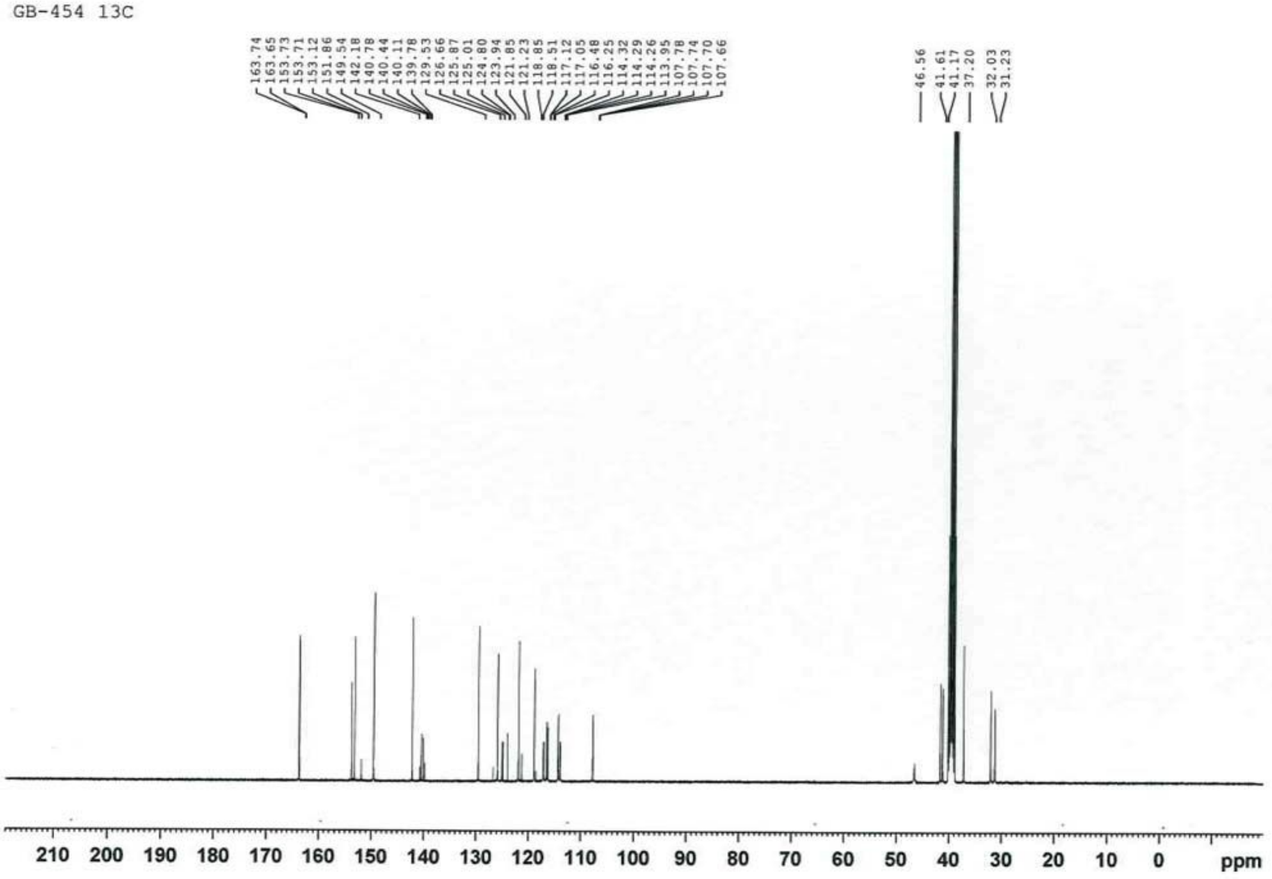

Figure S28. ^{1}H-NMR and ^{13}C-NMR of compound **13**.

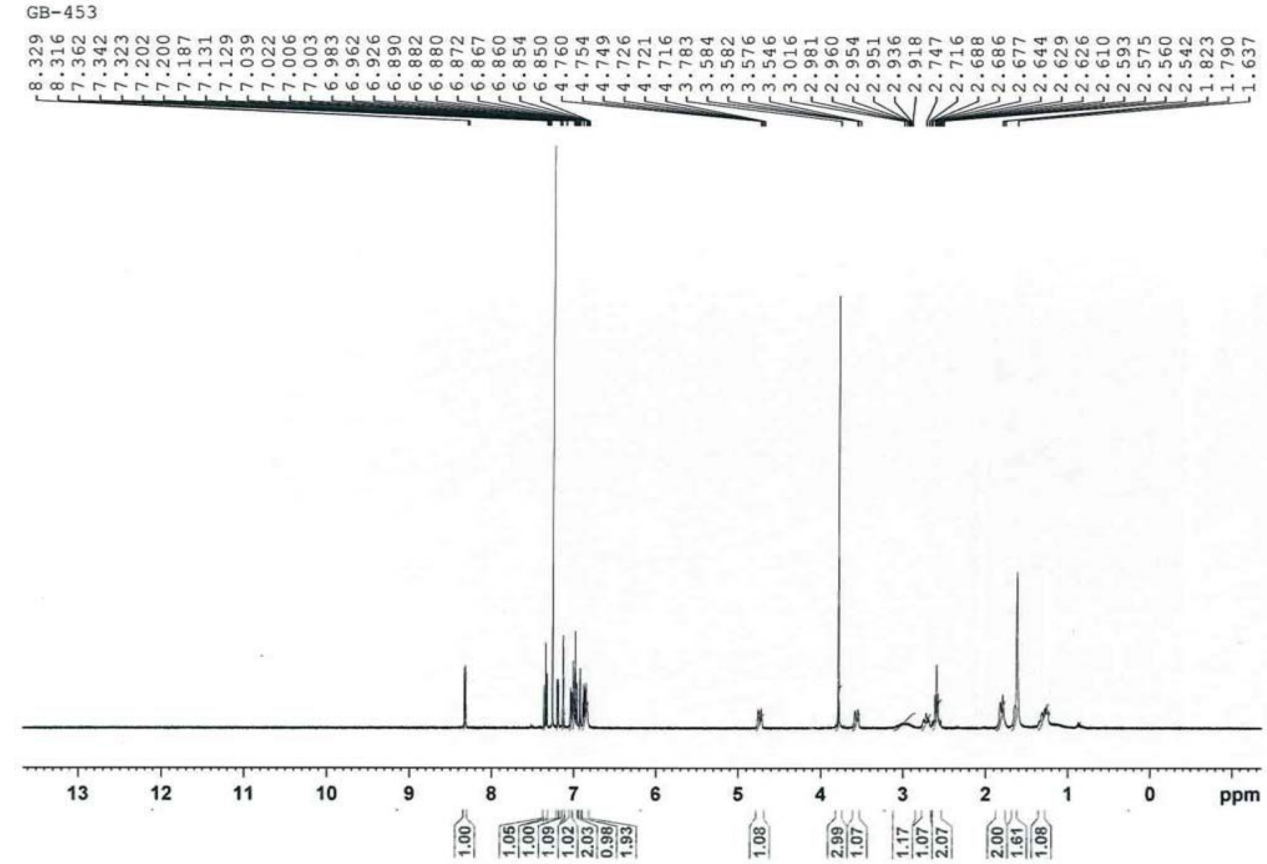

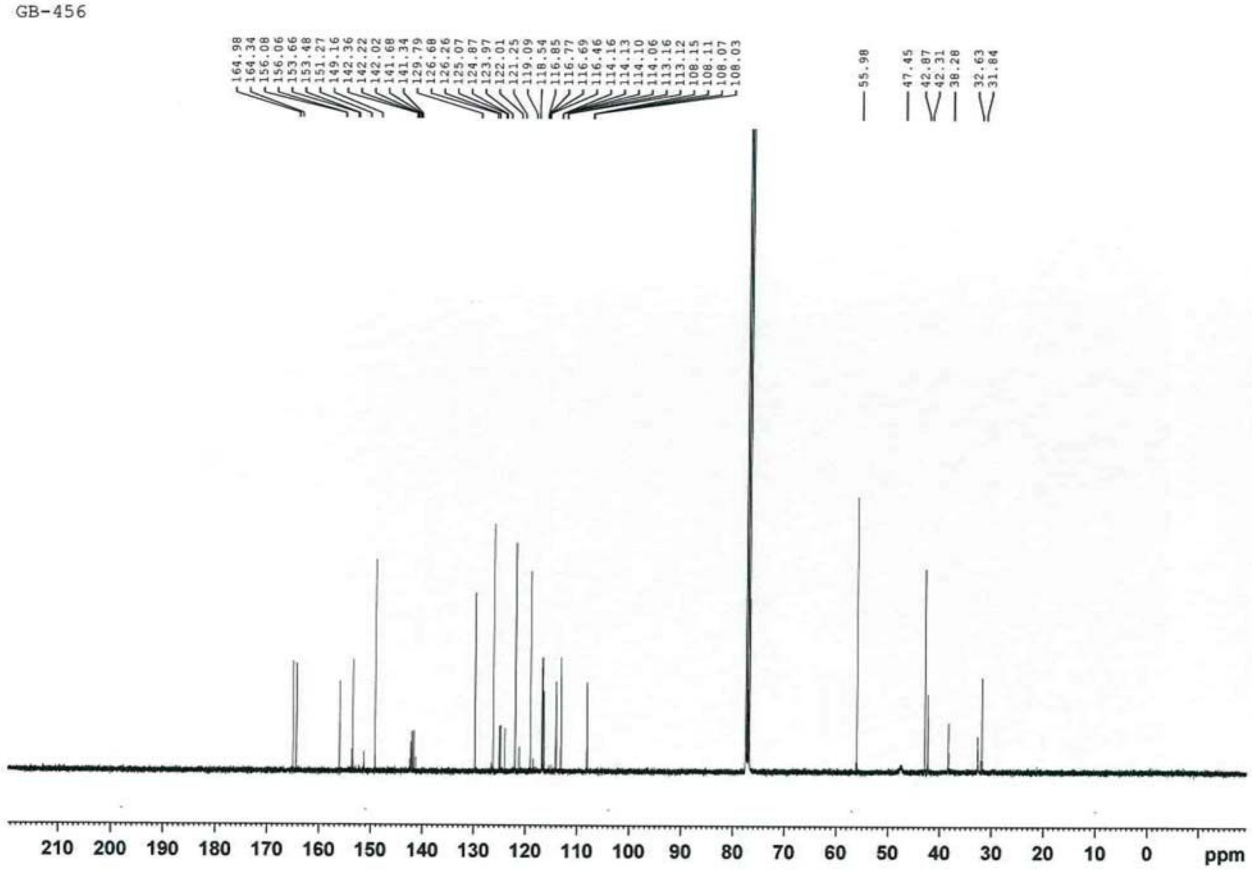

Figure S29. ^{1}H-NMR and ^{13}C-NMR of compound **40**.

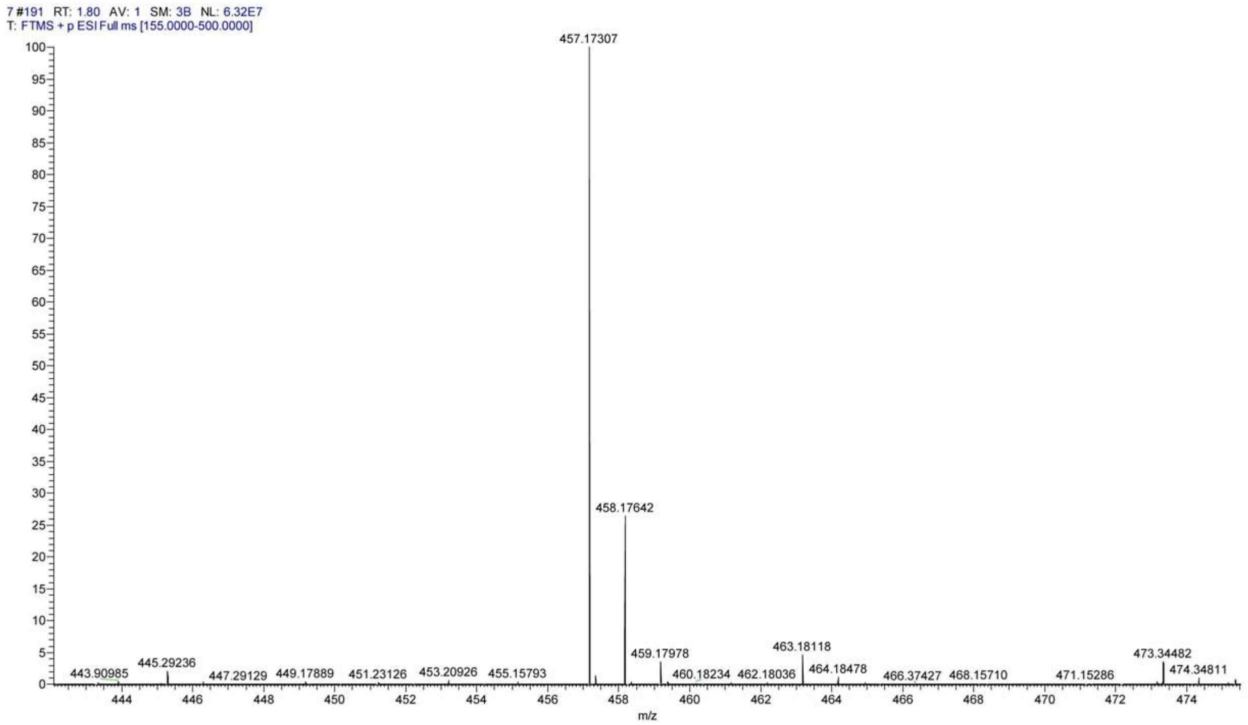

Figure S30. ESI-HRMS spectrum of compound **7**.

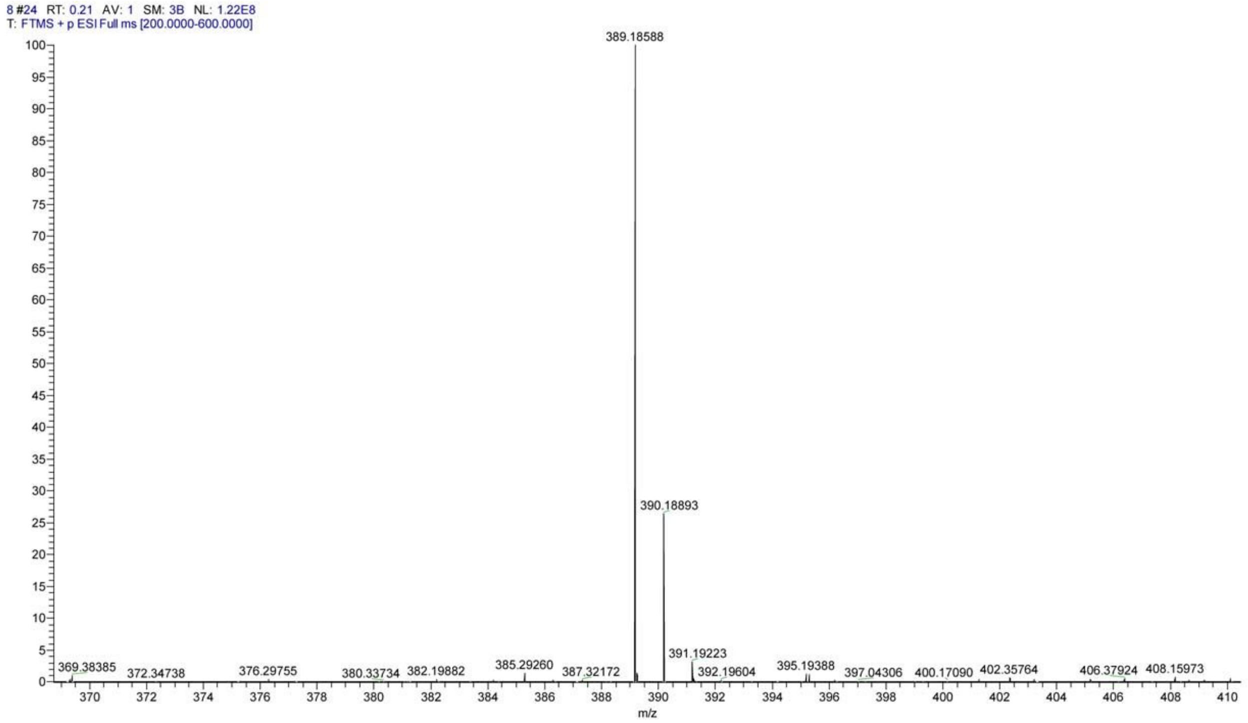

Figure S31. ESI-HRMS spectrum of compound **8**.

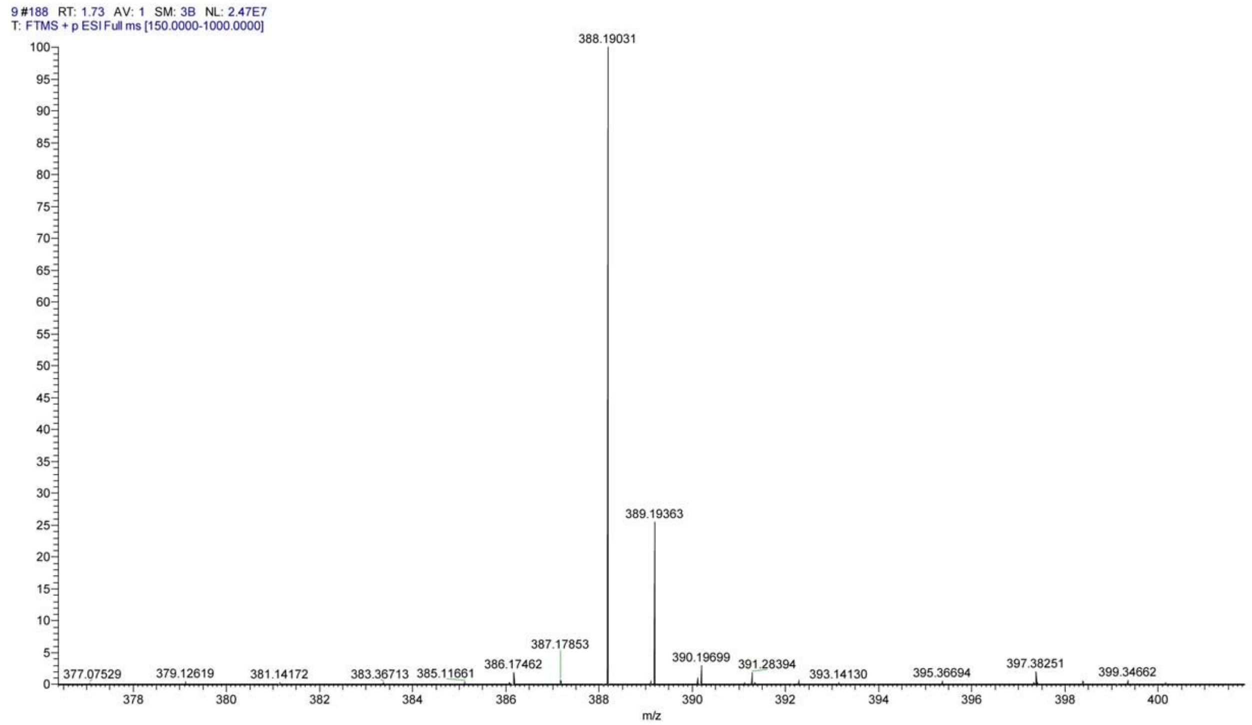

Figure S32. ESI-HRMS spectrum of compound **9**.

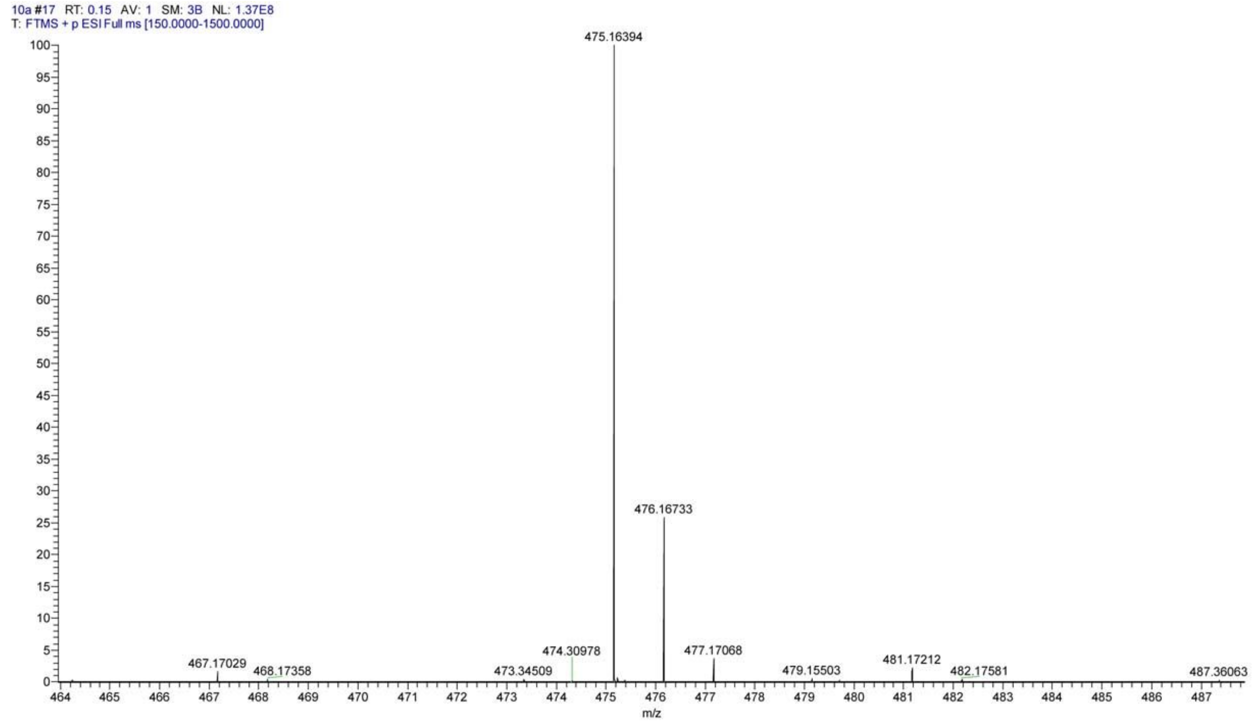

Figure S33. ESI-HRMS spectrum of compound **10a**.

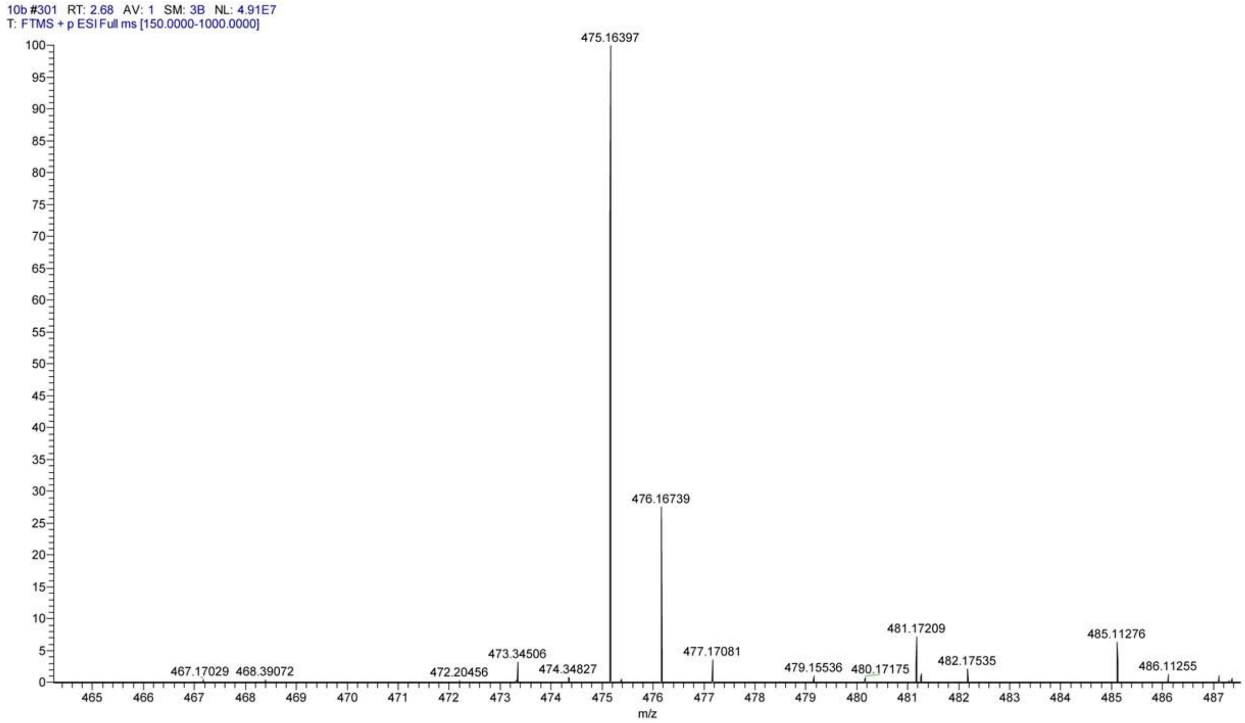

Figure S34. ESI-HRMS spectrum of compound **10b**.

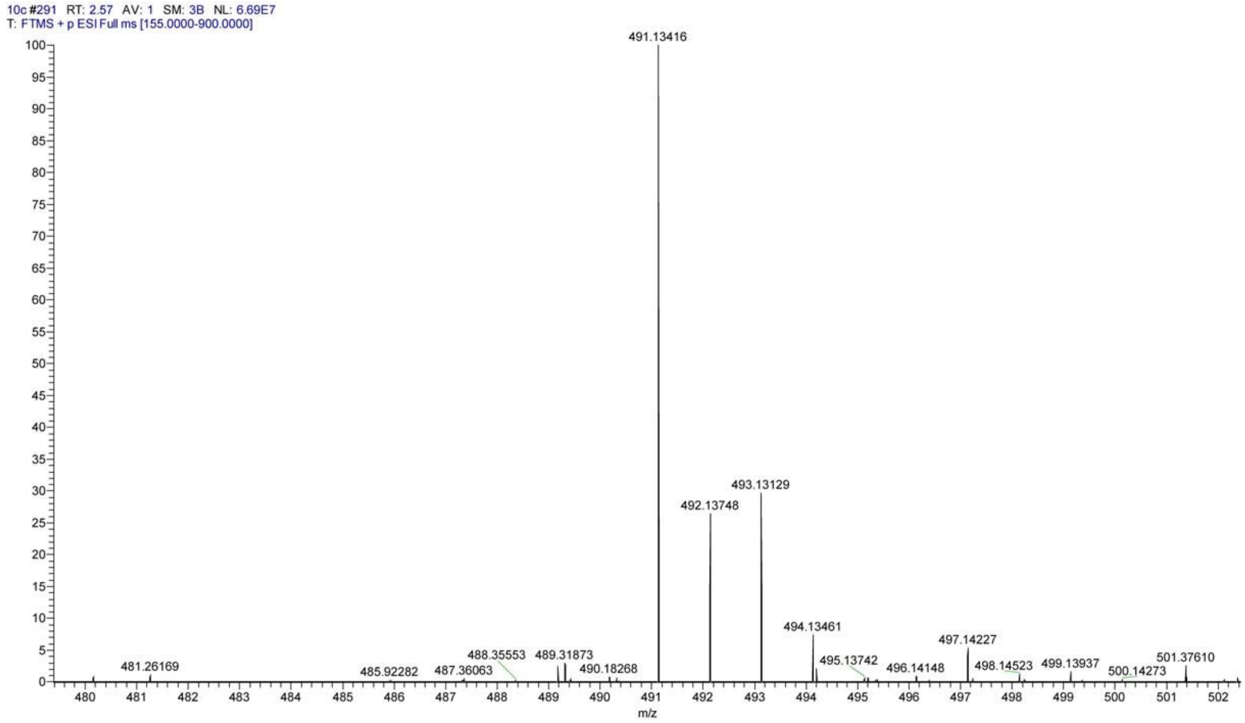

Figure S35. ESI-HRMS spectrum of compound **10c**.

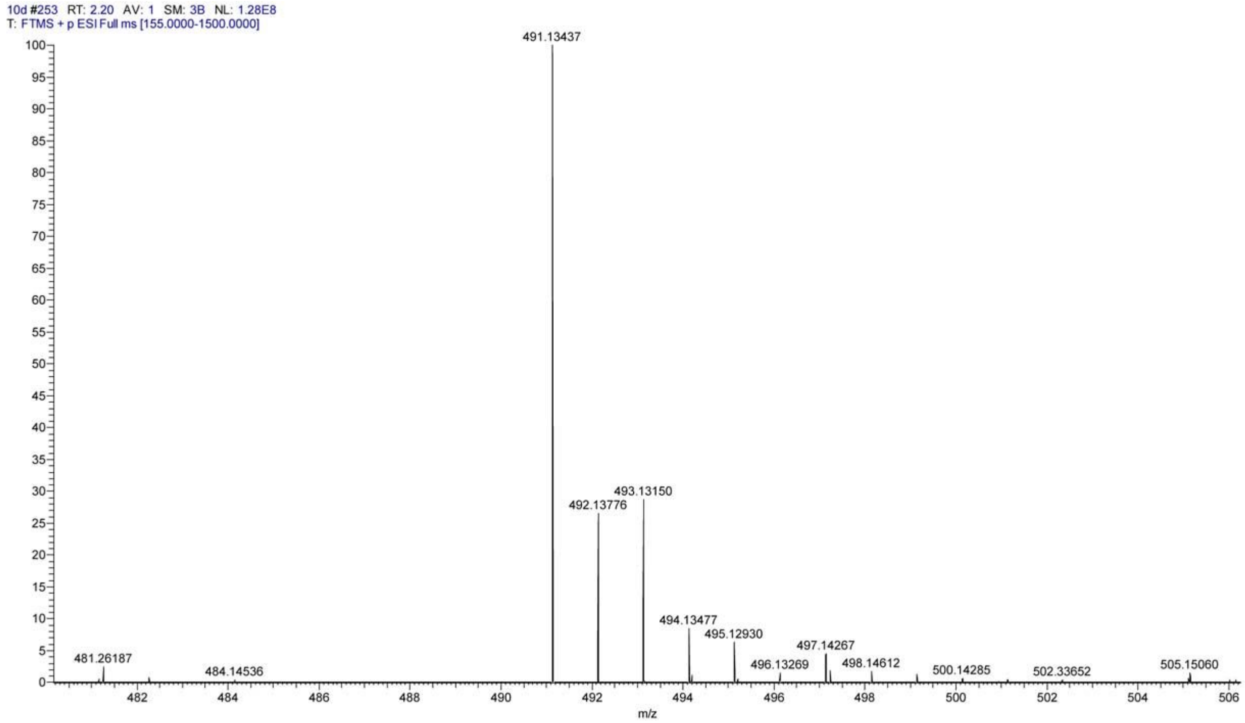

Figure S36. ESI-HRMS spectrum of compound **10d**.

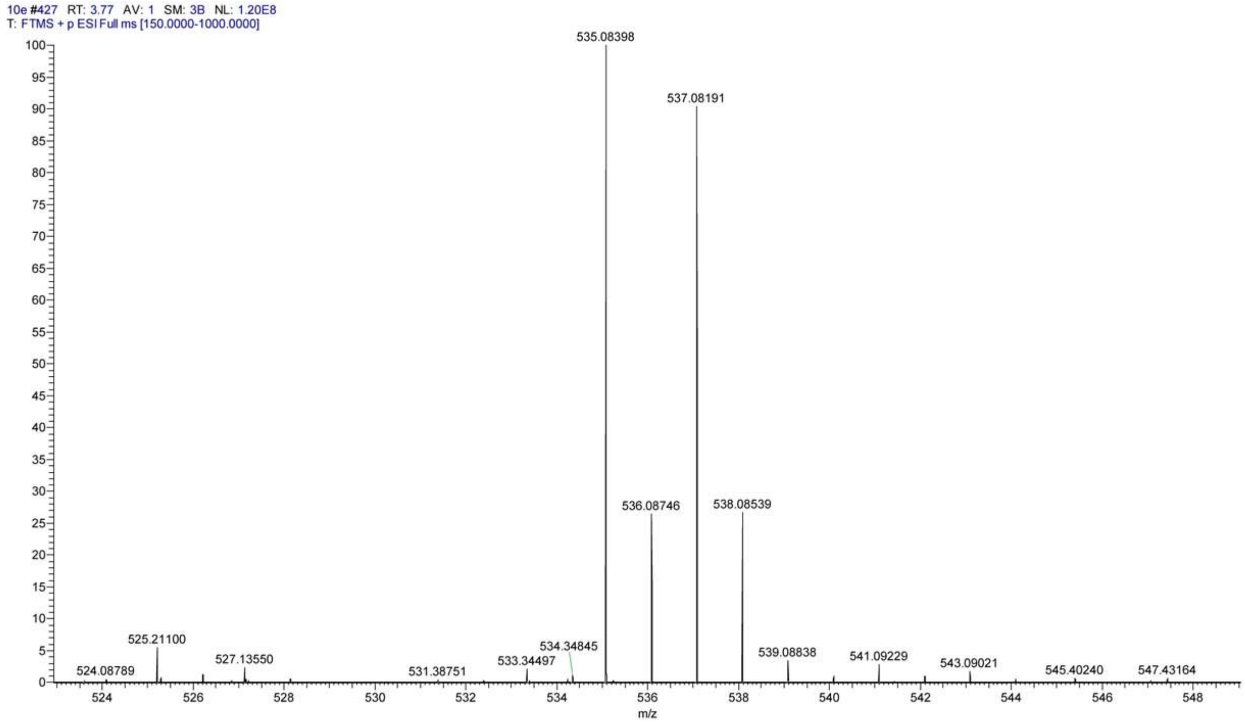

Figure S37. ESI-HRMS spectrum of compound **10e**.

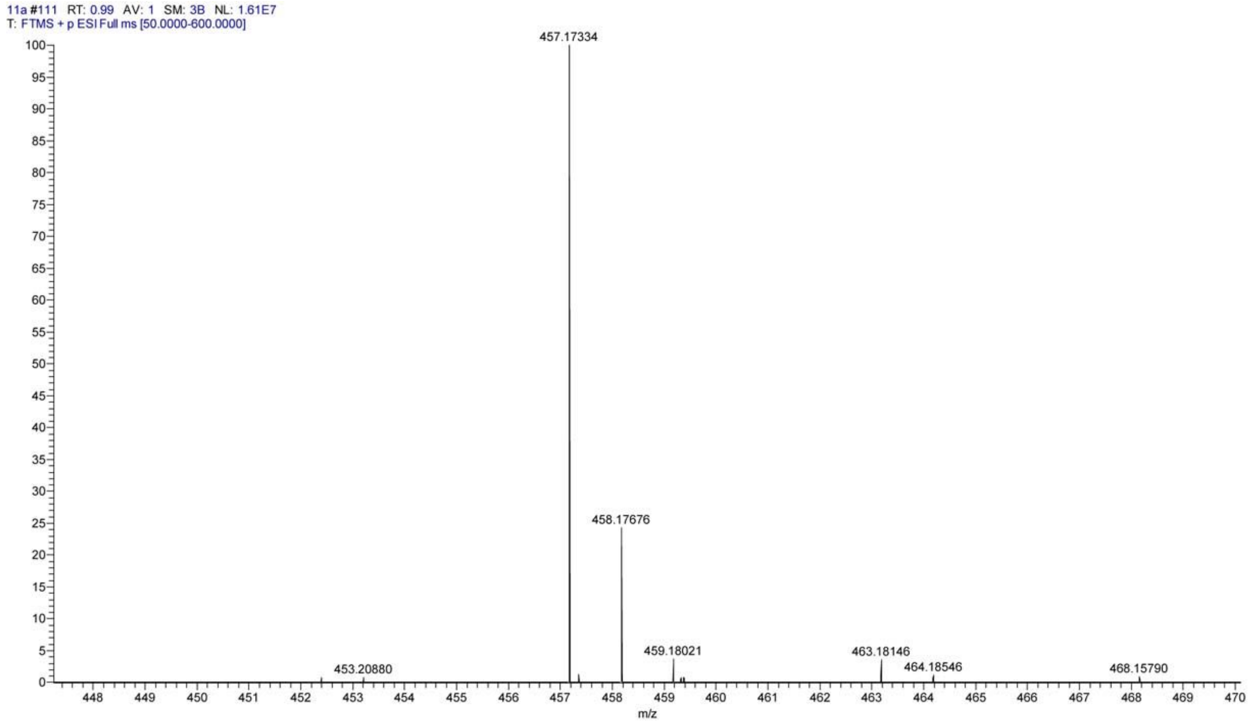

Figure S38. ESI-HRMS spectrum of compound **11a**.

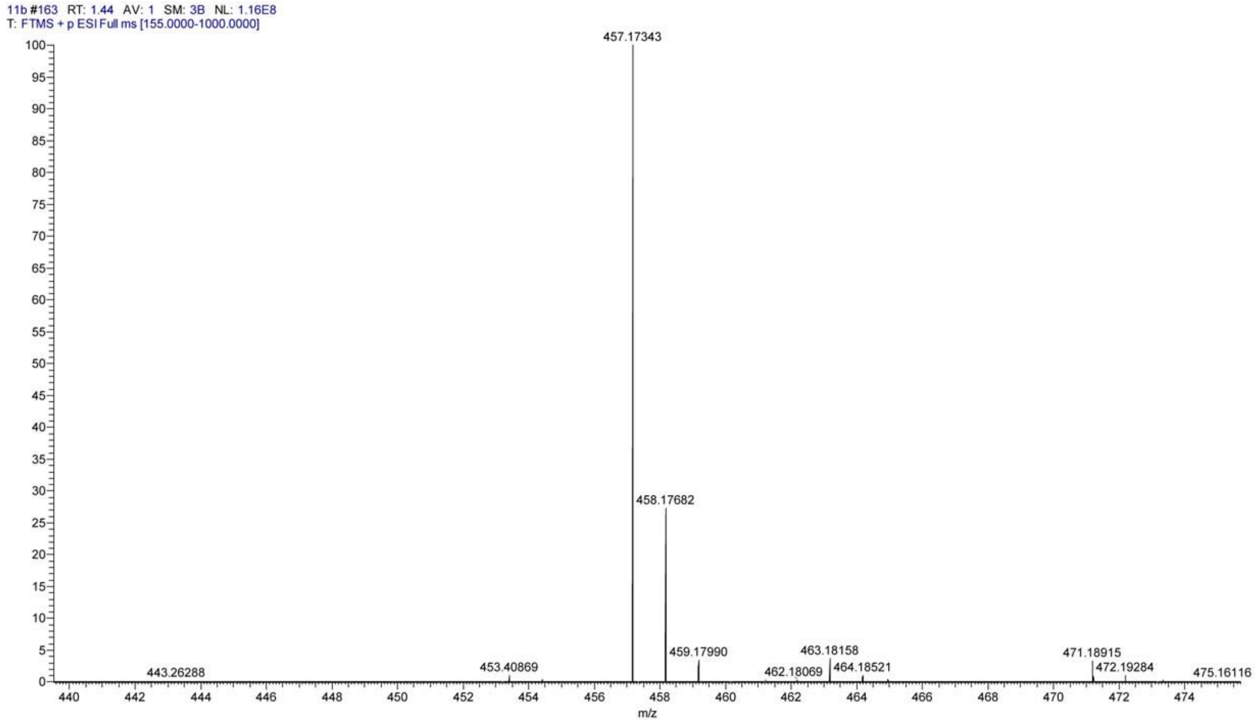

Figure S39. ESI-HRMS spectrum of compound **11b**.

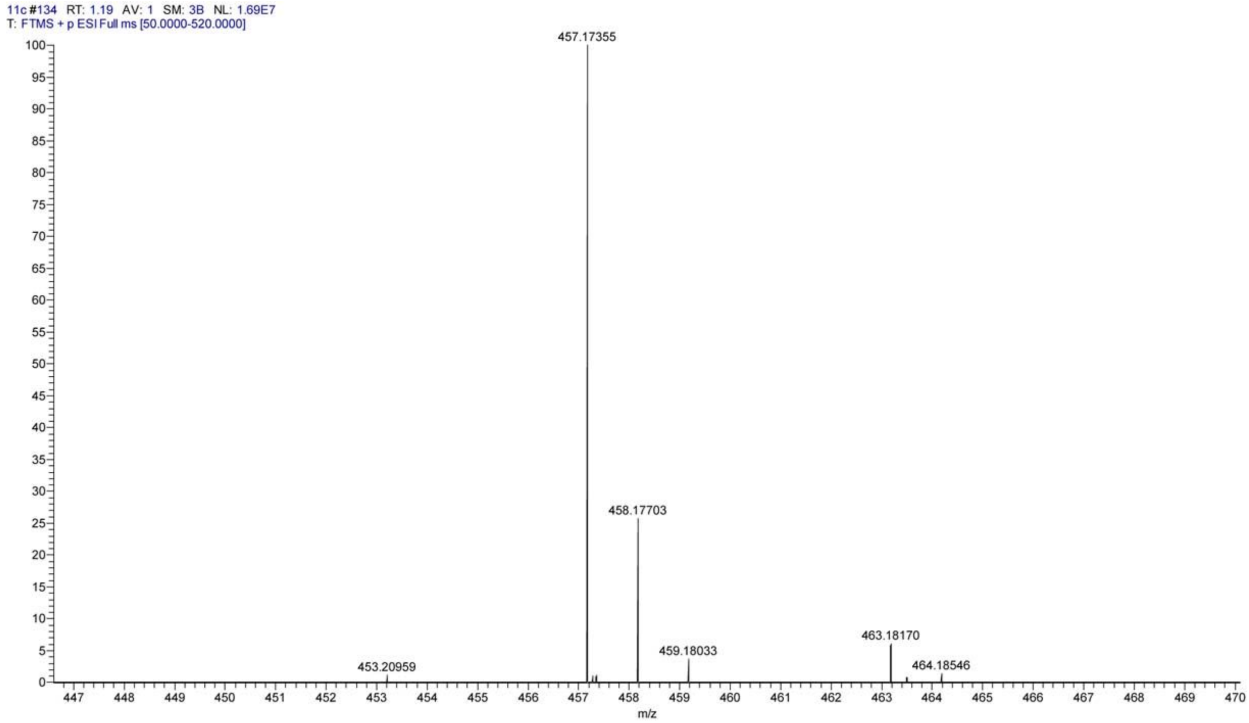

Figure S40. ESI-HRMS spectrum of compound **11c**.

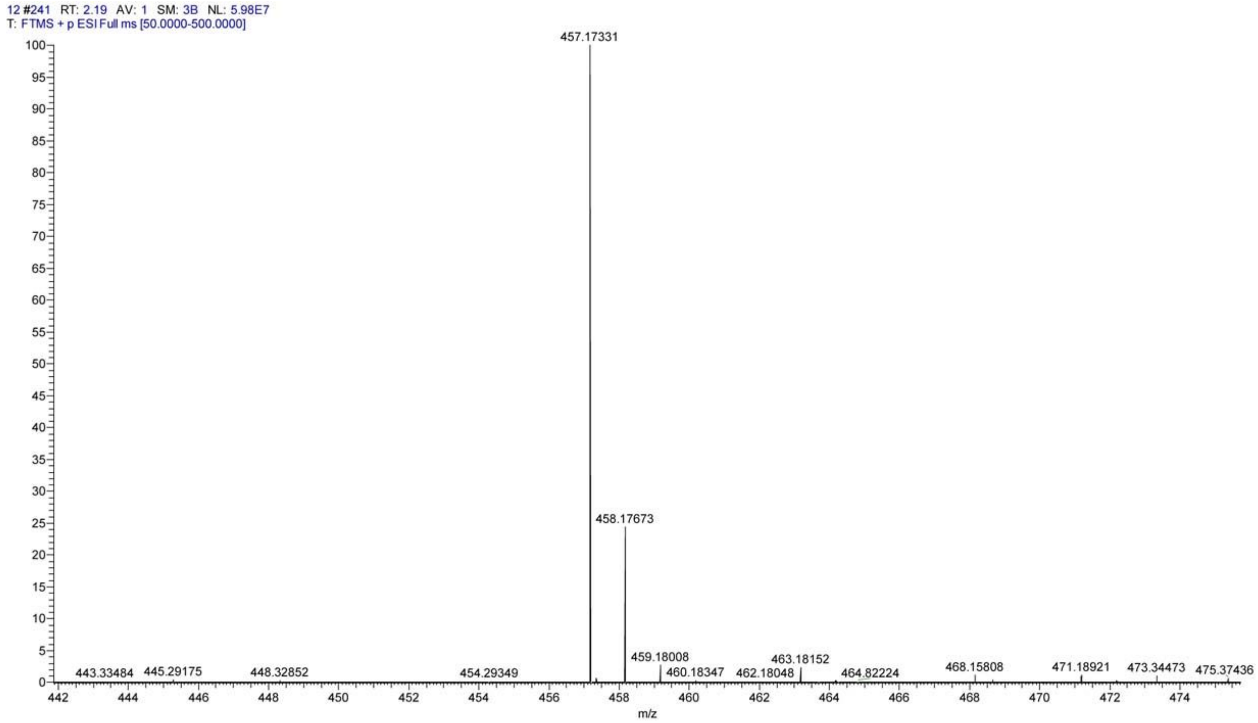

Figure S41. ESI-HRMS spectrum of compound **12**.

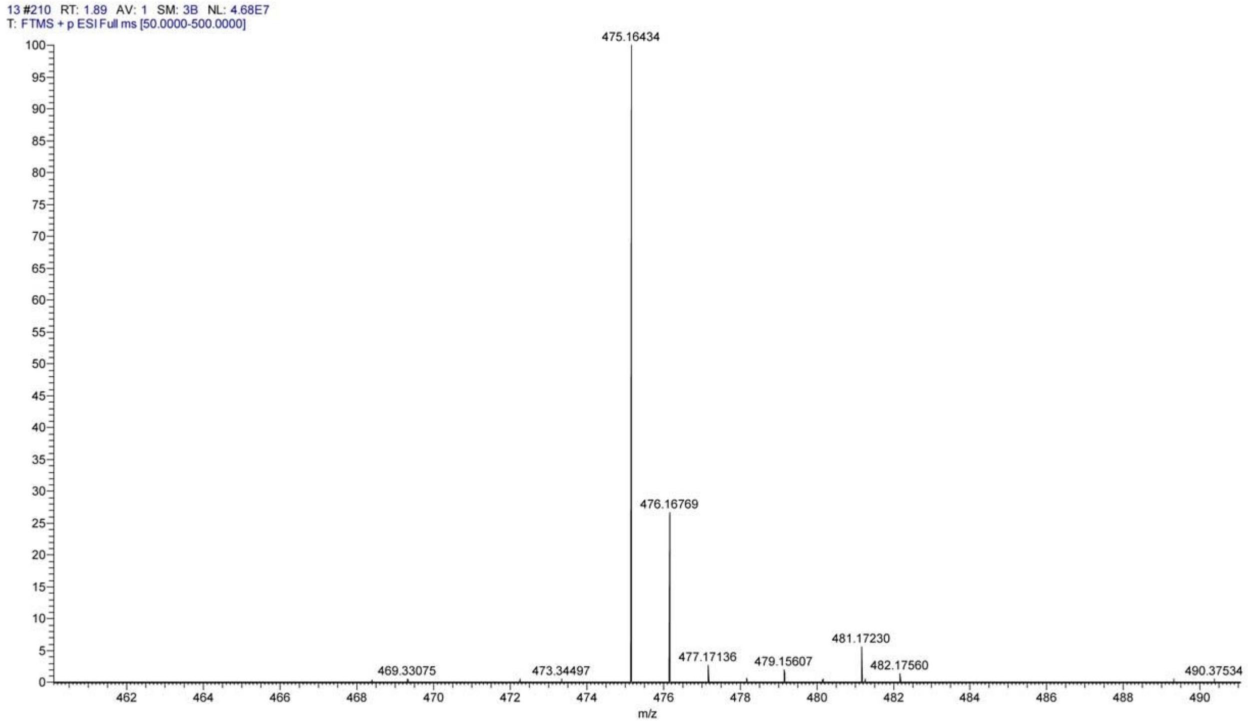

Figure S42. ESI-HRMS spectrum of compound **13**.

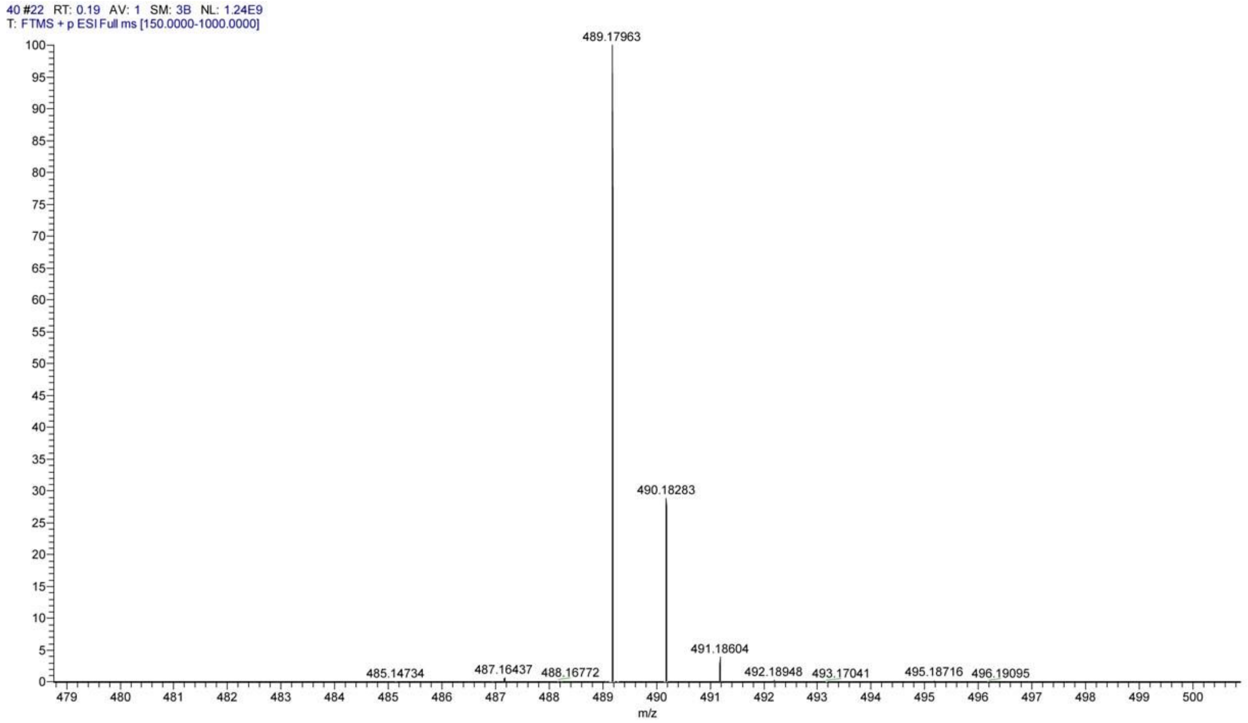

Figure S43. ESI-HRMS spectrum of compound **40**.

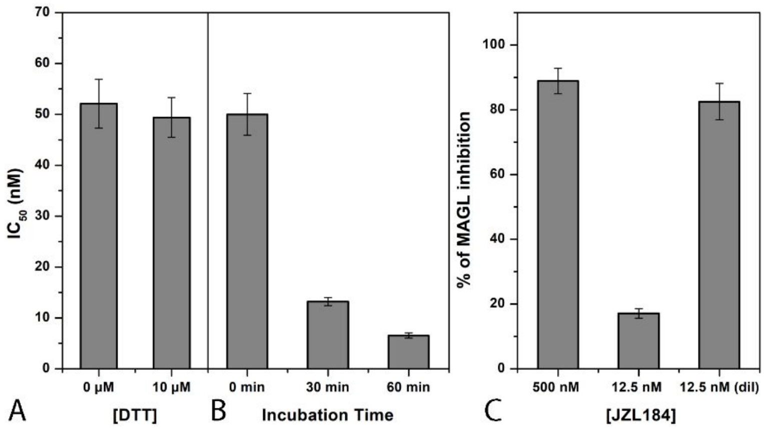

Figure S44. Analysis of the mechanism of MAGL inhibition of reference compound JZL-184. A) Effect of DTT on MAGL inhibition activity. B) IC_{50} (nM) values at different preincubation times with MAGL (0 min, 30 min and 60 min). C) Dilution assay: the first two columns indicate the inhibition percentage of the compound at a concentration of 500 nM and 12.5 nM. The third column indicates the inhibition percentage of the compound after dilution (final concentration = 12.5 nM).

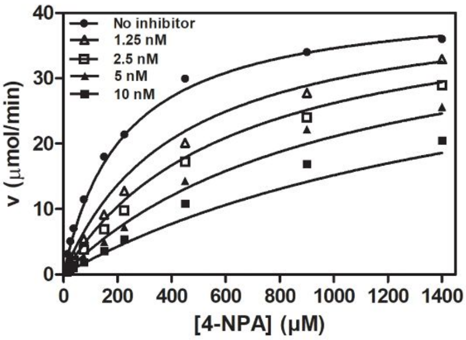

Figure S45. Inhibition of the activity of *h*MAGL and competitive binding of compound **13** (K_i = 1.42 ± 0.16 nM, α >10000).

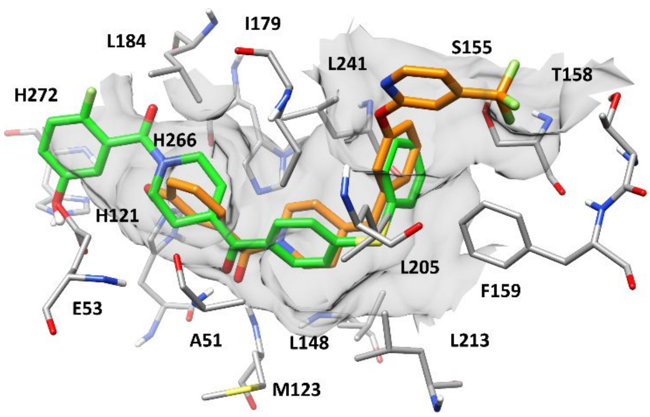

Figure S46. Minimized average structure of *h*MAGL in complex with **11b**, superimposed with the binding mode of **5b**. The inner surface of MAGL binding site is shown in gray.

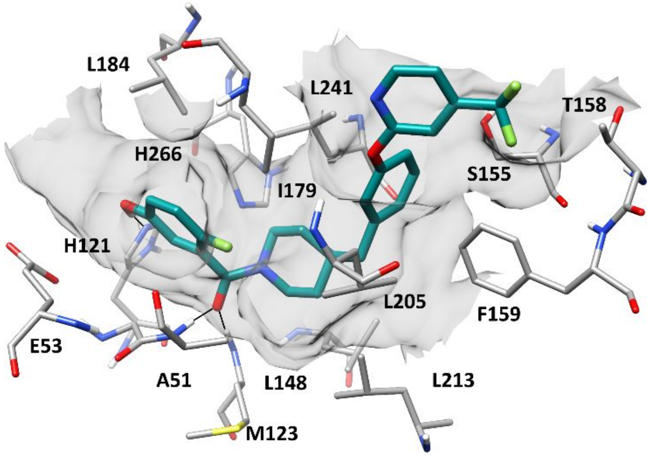

Figure S47. Minimized average structure of *h*MAGL in complex with compound **13.** The inner surface of MAGL binding site is shown in gray, while ligand-protein H-bonds are shown as black lines.
